# Dominance is common in mammals and is associated with trans-acting gene expression and alternative splicing

**DOI:** 10.1186/s13059-023-03060-2

**Published:** 2023-09-29

**Authors:** Leilei Cui, Bin Yang, Shijun Xiao, Jun Gao, Amelie Baud, Delyth Graham, Martin McBride, Anna Dominiczak, Sebastian Schafer, Regina Lopez Aumatell, Carme Mont, Albert Fernandez Teruel, Norbert Hübner, Jonathan Flint, Richard Mott, Lusheng Huang

**Affiliations:** 1 National Key Laboratory for Pig Genetic Improvement and Production Technology, Jiangxi Agricultural University, Nanchang, 330045 People’s Republic of China; 2 UCL Genetics Institute, University College London, London, WC1E 6BT UK; 3 Human Aging Research Institute and School of Life Science, Nanchang University, and Jiangxi Key Laboratory of Human Aging, Jiangxi, China; 4 School of Life Sciences, Nanchang University, Nanchang, China; 5 Centre for Genomic Regulation (CRG), The Barcelona Institute of Science and Technology, Barcelona, Spain; 6 BHF Glasgow Cardiovascular Research Centre, University of Glasgow, Glasgow, G12 8TA UK; 7 Cardiovascular and Metabolic Disorders Program, Duke-National University of Singapore Medical School, Singapore, Singapore; 8 Department of Medicine and Life Sciences, Universitat Pompeu Fabra, Barcelona, Spain; 9 Wellcome Trust Centre for Human Genetics, University of Oxford, Oxford, UK; 10 Departamento de Psiquiatría y Medicina Legal, Universitat Autonoma de Barcelona, Barcelona, Spain; 11 Genetics and Genomics of Cardiovascular Diseases Research Group, Max Delbrück Center (MDC) for Molecular Medicine in the Helmholtz Association, Berlin, Germany; 12 DZHK (German Center for Cardiovascular Research) Partner Site Berlin, Berlin, Germany; 13 Charité Universitätsmedizin Berlin, Berlin, Germany; 14 Department of Psychiatry and Behavioral Sciences, Brain Research Institute, University of California, Los Angeles, CA USA

## Abstract

**Background:**

Dominance and other non-additive genetic effects arise from the interaction between alleles, and historically these phenomena play a major role in quantitative genetics. However, most genome-wide association studies (GWAS) assume alleles act additively.

**Results:**

We systematically investigate both dominance—here representing any non-additive within-locus interaction—and additivity across 574 physiological and gene expression traits in three mammalian stocks: F2 intercross pigs, rat heterogeneous stock, and mice heterogeneous stock. Dominance accounts for about one quarter of heritable variance across all physiological traits in all species. Hematological and immunological traits exhibit the highest dominance variance, possibly reflecting balancing selection in response to pathogens. Although most quantitative trait loci (QTLs) are detectable as additive QTLs, we identify 154, 64, and 62 novel dominance QTLs in pigs, rats, and mice respectively that are undetectable as additive QTLs. Similarly, even though most cis-acting expression QTLs are additive, gene expression exhibits a large fraction of dominance variance, and trans-acting eQTLs are enriched for dominance. Genes causal for dominance physiological QTLs are less likely to be physically linked to their QTLs but instead act via trans-acting dominance eQTLs. In addition, thousands of eQTLs are associated with alternatively spliced isoforms with complex additive and dominant architectures in heterogeneous stock rats, suggesting a possible mechanism for dominance.

**Conclusions:**

Although heritability is predominantly additive, many mammalian genetic effects are dominant and likely arise through distinct mechanisms. It is therefore advantageous to consider both additive and dominance effects in GWAS to improve power and uncover causality.

**Supplementary Information:**

The online version contains supplementary material available at 10.1186/s13059-023-03060-2.

## Background

Dominance arises from non-additive interactions between different alleles within a locus. The pathways that cause dominance still remain to be clarified, despite intense scrutiny [1–3]; suggested explanations include haplo-sufficiency (when a single working copy of one gene is sufficient for normal function) [4, 5], antimorphs (when the mutant product of one gene interacts and interferes with the normal product) [6–8], hypomorphs (when one allele has a partial or complete loss-of-function) [9], and antagonistic pleiotropy (when one allele is beneficial to some traits while deleterious to others) [10–12].

In the quantitative genetics model proposed by Fisher in 1916 [13–15], the genetic variance, or heritability, of a quantitative trait is partitioned into additive, dominant, and epistatic components, each of which are aggregates of many smaller contributions within and between multiple causal loci. It follows that understanding dominance at the phenotypic level depends on understanding dominance within each causal locus, and on clarifying the causal molecular pathways.

A given biallelic locus is additive if the phenotypic effect of the heterozygote is the mean of that of the two homozygotes. In this study, we define dominance to mean any non-additive within-locus interaction, classified as partial (PD), complete (CD), or over-dominance (OD), according to whether the phenotypic effect of the heterozygote lies within the range spanned by the homozygotes but is unequal to their average, or is equal to one of the homozygote effects, or is outside their range [16–18] (Additional file 1: Figure S1.1). Any possible additive or dominance relationship between a trait and a biallelic locus can be modeled by a combination of additive and complete dominance effects, and the presence of dominance in this wider non-additive sense is therefore testable by comparing the fit of a purely additive model to a model with both additive and dominance effects. Computational methods to detect dominance in genome-wide association studies (GWAS) have been developed by our group [19] and others [20–27].

Although most quantitative genetics studies assume additivity, dominance effects—where investigated—have been observed in GWAS, heritability estimation, genomic selection, and prediction. Crosses between inbred strains often reveal the closely related phenomenon of heterosis. Dominance quantitative trait loci (QTLs) have been mapped in animals (cattle [28–34], pig [35–38], sheep [39], chicken [40, 41]), plants (maize [42–45], wheat [46], rice [47], sunflower [48], *Arabidopsis *[49], *Primulina *[50]) and in a few studies in humans [51–56]. In cattle, where dominance effects have been investigated most intensively, recessive QTLs are known for lactation, growth, and developmental traits [31, 33]. Similarly in pigs, dominance QTLs are associated with the number and weight of piglets born [38], number of teats [37], meat quality [36], and growth traits [35]. In plants, dominance QTLs are associated with disease resistance (shoot fly in maize [43] and stripe rust resistance of wheat [46]) and growth (leaf orientation in maize [45], flowering time in *Arabidopsis* [49] and sunflower hybrids [48], and hybrid male sterility of *Primulina* [50]). Consistent with these observations, a large fraction of dominance heritability frequently occurs in cattle (yearling weight [57], growth [58], milk production [59], and reproduction [60]) and pigs (sow longevity [61], daily gain [62, 63], number of teats [63], backfat [63, 64], and growth [64]).

In contrast with these findings, studies in humans have generally reported that both dominance variance components are small [65–68] and dominance-associated loci [54, 56, 69] are relatively rare. One potential explanation might be the prevalence of low-frequency alleles in human and other large random mating populations. In contrast, experimental and artificially bred populations exhibit limited haplotype diversity and higher allele frequencies. The power to detect dominance QTLs and to predict dominance phenotypes depends critically on the frequency of the rarer of the two homozygote genotypes and is consequently attenuated at lower allele frequencies, as shown in a recent study of recessive human disease [55].

There are also practical reasons why dominance is often ignored. First, modelling dominance requires extra degrees of freedom in fixed effects models, potentially reducing the power to detect purely additive effects. Second, if one is to model dominance effects using the mixed model framework, it follows that both additive and dominance variance components should be included in genetic background effects, which is computationally challenging. For these reasons, most GWAS in humans only consider additive variance components. However, where dominance heritability is large, this shortcut is potentially unsound and might reduce the power to detect genetic associations.

Another important reason to consider dominance is that understanding the relationship between dominance at the level of gene expression and at the level of physiological phenotype may be key to establishing causal mechanisms. In yeast [70, 71], plants [72], flies [73], fish [74], and mice [75] dominance gene expression is associated with trans-acting effects and with structural variations such as translocations that silence or otherwise modulate the expression [76]. In contrast, most cis-acting expression QTLs (eQTLs) are additive. These phenomena suggest how dominance might arise at the molecular level, but their prevalence in mammals is under-explored.

In this study, we systematically investigate dominance across physiological and gene expression traits in three mammalian species, namely pigs [77], rats [78], and mice [79]. These populations were chosen because of the wealth of genotypes and phenotypes available combined with gene expression measured on large subsets of the same animals. Within each population, we analyze their dominance and additive genetic architectures through variance decomposition, QTL and eQTL mapping. Additionally, in the rats, we use RNA-seq data to relate dominance to the expression of alternative isoforms, revealing a novel potential mechanism for dominance.

## Results

### Population characteristics

We integrated and analyzed published multi-phenotype and multi-omics data from three mammalian stocks: (1) F2 intercross pigs (hereafter F2 pigs) [77], containing 1005 progeny derived from 2 White Duroc boars mated with 17 Erhualian sows, with 253 complex traits measured related to growth, fatness, meat quality, and blood. In addition, we analyzed their digital gene expression data of liver and muscle [80]; (2) heterogeneous stock rats (HS rats) [78], encompassing 1407 individuals descended from eight inbred founder strains (ACI/N, BN/SsN, BUF/N, F344/N, M520/N, MR/N, WKY/N and WN/N), in which 220 physiological traits and previously unpublished RNA-seq data of amygdala and heart samples were measured; (3) heterogeneous stock mice [79] (HS mice), comprising 2002 individuals descended from eight inbred founder strains (A/J, AKR/J, BALBc/J, CBA/J, C3H/HeJ, C57BL/6 J, DBA/2 J, and LP/J), with measurements of 125 physiological traits and microarray gene expression data of hippocampus, liver, and lung [81]. The chromosomes of the HS rats and mice are fine-grained mosaics of their respective inbred founder strains. The F2 pigs are not descended from inbred founders because individuals from the same pig breed are not genetically identical. The populations and datasets used, including the traits mapped in each population are summarized in Additional file 2: Table S1 and Table S2.

Each phenotype was normalized, and the effects of covariates removed, as described previously [77–79]. All subsequent analyses used these normalized residuals. In each population, we removed SNPs with minor allele frequency (MAF) < 0.05, missing rate > 0.1 or if the rarest genotype occurred in fewer than 10 individuals. The numbers of SNPs passing these quality control steps was respectively 39,298 (pig), 244,786 (rat), and 9142 (mouse).

### Dominance accounts for about a quarter of genetic variance of organismal traits

**Fig. 1:**
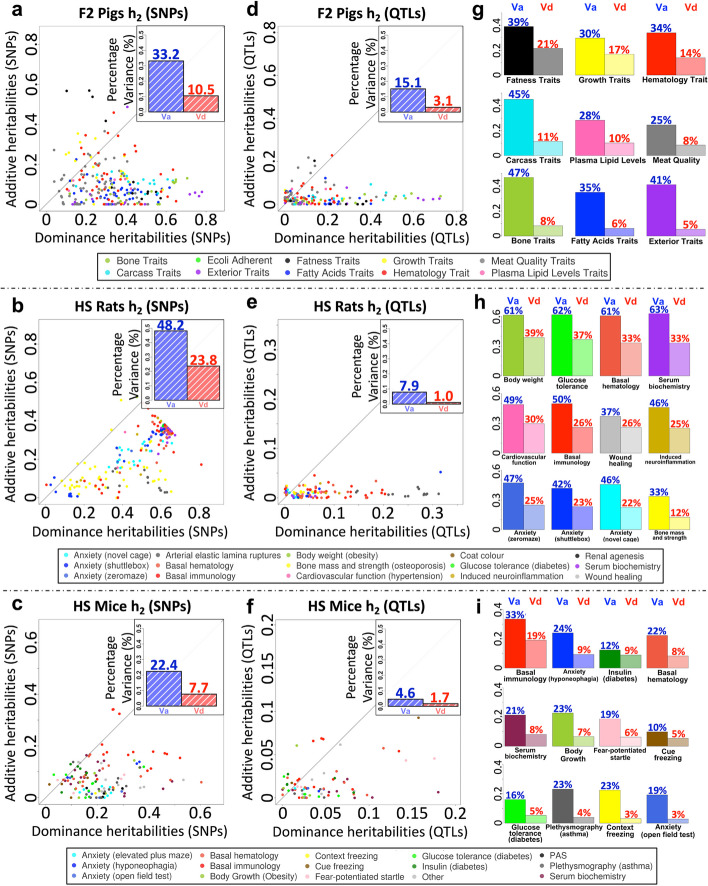
Additive and dominance heritabilities across organismal traits in three populations. **a**–**f** Scatter plots of Additive phenotypic variance component ${V}_{a}$ (*x*-axis) *vs* dominance component ${V}_{d}$ (*y*-axis), either estimated from genome-wide SNPs (**a**–**c**) or from accumulated significant QTLs (**d**–**f**) of 241 traits in F2 pigs, 206 traits in HS rats, and 124 traits in HS mice respectively. Within each scatter plot, each dot denotes a trait, scaled to have unit variance so that variance components are also heritabilities ${h}_{a}^{2}, {h}_{d}^{2}$. Dot colors show trait categories as tabulated in the left insets. The marginal histograms display distributions of ${V}_{a}$ (blue, top) and ${V}_{d}$ (red, right) of traits in each population, and their average values are indicated by upper-right inset bar plots. **g**–**i** Average ${V}_{a}$ (left) ${V}_{d}$ (right) for each population, classified by trait category

We used these SNP sets to construct additive and dominance genetic relationship matrices (GRMs) and performed quantitative genetic analysis. We dissected the contributions of dominance to the 584 organismal traits measured across the three populations, by simultaneously estimating both additive $\left({V}_{a}\right)$ and dominance $\left({V}_{d}\right)$ genetic variance components from the GRMs in each trait using GCTA [65]. Each phenotype was first adjusted to remove covariates and scaled to have unit variance, so these components also represent heritabilities (Additional file 3: Table S2). The relationships between ${V}_{a}$ and ${V}_{d}$ in each population are shown as scatter plots (Fig. 1a–c) and bar plots (Additional file 1: Fig. S1.2 a-c). Across 425 traits with nonzero dominance variance (${V}_{d}>0.05$), ${V}_{a}/{V}_{d}\approx 3$, i.e., dominance accounts for about one quarter of the genetic variance. In F2 pigs, ${\overline{V} }_{a}$:${\overline{V} }_{d}=0.33:0.11$ across $n=163$ traits. HS rats exhibit slightly higher average heritabilities (${\overline{V} }_{a}:{\overline{V} }_{d}=0.48$:$0.24$, $n=187$), while in the HS mice they are lower (${\overline{V} }_{a}: {\overline{V} }_{d}=0.22$:$0.08$, $n=75$).

In general, additive genetic effects explain more phenotypic variance than dominance effects across all three populations, in line with previous studies [77–79]. However, many traits have important dominance contributions. In pigs, rats and mice, respectively, there are 52, 143, and 15 traits (21.6%, 69.8%, 12.2%) where ${V}_{d}>0.15$, and 27, 8, and 13 (11.3%, 3.9%, 10.6%) where ${V}_{d}>{V}_{a}$ (Additional file 3: Table S2 and Additional file 1: Fig. S1.2). In F2 pigs, fatness, growth, and hematology-related traits exhibit the largest dominance effects (Fig. 1a, g), while in rats and mice, dominance is most noticeable in immunology, hematology, and serum biochemistry-related traits (Fig. 1b,c, h–j). We show below that dominance and over-dominance QTLs are more often seen in those traits with higher dominance variance components.

### Mapping dominance QTLs improves GWAS sensitivity

We used ADDO [19] to perform a mixed model GWAS for each organismal trait, modelling both additive and dominance fixed effects at each focal SNP and including additive, dominance, and environmental variance components simultaneously to model background effects. This Add-Dom (or “AD”) Model exhibits better calibration of *P*-values than a model solely including additive effects (named the Add or “A-Model”) [19]. At each SNP, we compared the AD Model to either a null model with neither additive nor dominance QTL or to a model with additive SNP effect only, but fitting both AD variance components. We applied two Bonferroni *P*-value significance thresholds, (i) approximate 5% genome-wide significance (0.05/N_SNP_) and (ii) suggestive significance (1/N_SNP_, corresponding to $-{\mathrm{log}}_{10}P=4.5, 4.8, 3.9$ in pigs, rats and mice respectively).

**Fig. 2:**
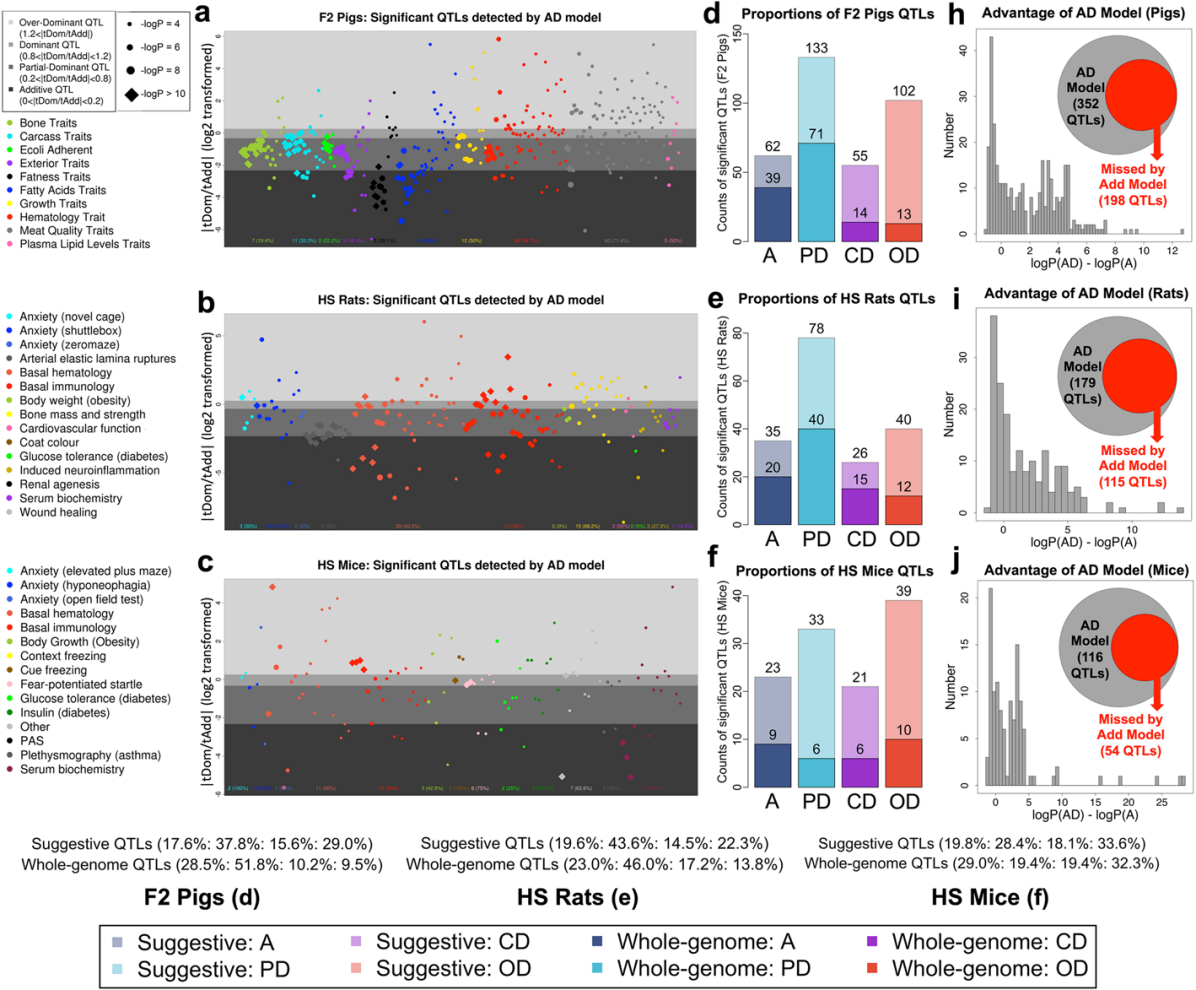
Classification of dominance QTLs and power to detect QTLs using the AD model. **a–c** Scatter plots showing QTLs detected by the AD Model at suggestive significant thresholds (one false positive expected per genome scan) in F2 pigs (**a**), HS rats (**b**), and HS mice (**c**). Each dot denotes a QTL, colors of dots represent trait categories, dot sizes represent the significance level (diamond points are those loci with − log_10_(*P*) values > 10) and vertical *y*-axis positions of the dots are their ${\mathrm{log}}_{2}\left|{t}_{Dom}/{t}_{Add}\right|$ values, with background gray shades representing their classification from the bottom up as additive, partial-dominant, complete-dominant, and over-dominant. **d**–**f** Bar plots of the counts of additive (blue), partial-dominant (sky blue), dominant (purple), and over-dominant (red) QTLs in each population. Light colors stand for the counts of suggestive significant QTLs and dark colors for whole genome significant QTLs. **h**–**j** Distribution of the difference between − log_10_(*P*) values of peak SNPs of suggestive significant QTLs detected by AD model compared to A model in each population. The gray and red inset circles show the numbers of QTLs detected by AD but missed by A models

We adapted a long-standing definition—the degree of dominance [16, 18]—to classify QTLs. We first computed the absolute values of the ratios of the T-statistics of the additive (${t}_{Add}$) and dominance (${t}_{Dom}$) effect estimates at each QTL, and then applied the following classification thresholds; additive (A-QTL): 0–0.2; partial-dominance (PD-QTL): 0.2–0.8; complete-dominance (CD-QTL): 0.8–1.2; over-dominance (OD-QTL): > 1.2. We recognize that since the ratio $\left|{t}_{Dom}/{t}_{Add}\right|$ is continuous, the classification boundaries are arbitrary. These thresholds are shown graphically in Fig. 2 and Additional file 1: Fig. S2.1. For a clearer visualization—but which does not change the classifications—we plot the log transformed ratios ${\mathrm{log}}_{2}\left|{t}_{Dom}/{t}_{Add}\right|$ in Fig. 2a–c. Un-transformed plots are shown in Additional file 1: Fig. S2.1 a-f.

Using suggestive genome-wide significance thresholds, the AD Model detected 352 QTLs for 182 F2 pig traits, 179 QTLs for 119 HS rat traits, and 116 QTLs for 73 HS mice traits (Fig. 2d–f and Additional file 4: Table S3), of which 137, 87, and 31 QTLs were genome-wide significant, with average logP-thresholds of 5.8, 6.1, and 5.2 for pig, rat, and mouse, respectively. We report results for the top SNP at each QTL in Additional file 5: Table S4. The ${\mathrm{log}}_{2}\left|{t}_{Dom}/{t}_{Add}\right|$ ratios for each QTL, categorized by population and class of phenotype, are plotted in Fig. 2a–c, and the genome-wide distributions of QTLs shown as porcupine plots in Additional file 1: Fig. S2.1 g-i. Similar proportions of QTL types occur in each population, and dominance QTLs are common throughout (Fig. 2d–f). On average, 16% of suggestive QTLs are complete-dominant and 28.3% over-dominant.

The AD Model has consistently greater power to detect QTLs (Additional file 1: Fig. S2.1 d-f) compared to the A Model (Additional file 1: Fig. S2.1 a-c), especially for CD and OD QTLs. Among all suggestive QTLs, 44.3% are detected by the AD Model but absent from the A Model (Fig. 2h–j, 43.8%, 35.8%, 53.4% for pig, rat, and mouse). These comprise 100 (98%) OD QTLs and 39 (70.9%) CD QTLs in F2 pigs, with similar counts of 15 (57.7%) and 35 (87.5%) in HS rats, and 16 (76.2%) and 34 (87.2%) in HS mice. In addition, the AD Model improved the − log_10_(*P*) values of 119 (33.8%) pig QTLs by more than 4 units compared to the A model, and similarly in for 52 (29.1%) rat QTLs and 40 (34.5%) mouse QTLs (Additional file 5: Table S4). Most newly detected or improved QTLs relate to hematology and immunology traits, consistent with the variance decomposition results.

We also investigated the power of the “D Model” which mapped purely dominant QTLs (Methods). Among suggestive SNPs detected by the AD, A, or D Models (Additional file 1: Fig. S2.2 a-c), there was a large increase in − log_10_(*P*) values of significant SNPs (either uniquely or concurrently) detected by AD compared to both A (Additional file 1: Fig. S2.2 d-f) and D Models (Additional file 1: Fig. S2.2 g-i). Thus, the AD model is uniformly more powerful than either simpler model and improves the detection and resolution of QTLs.

**Fig. 3:**
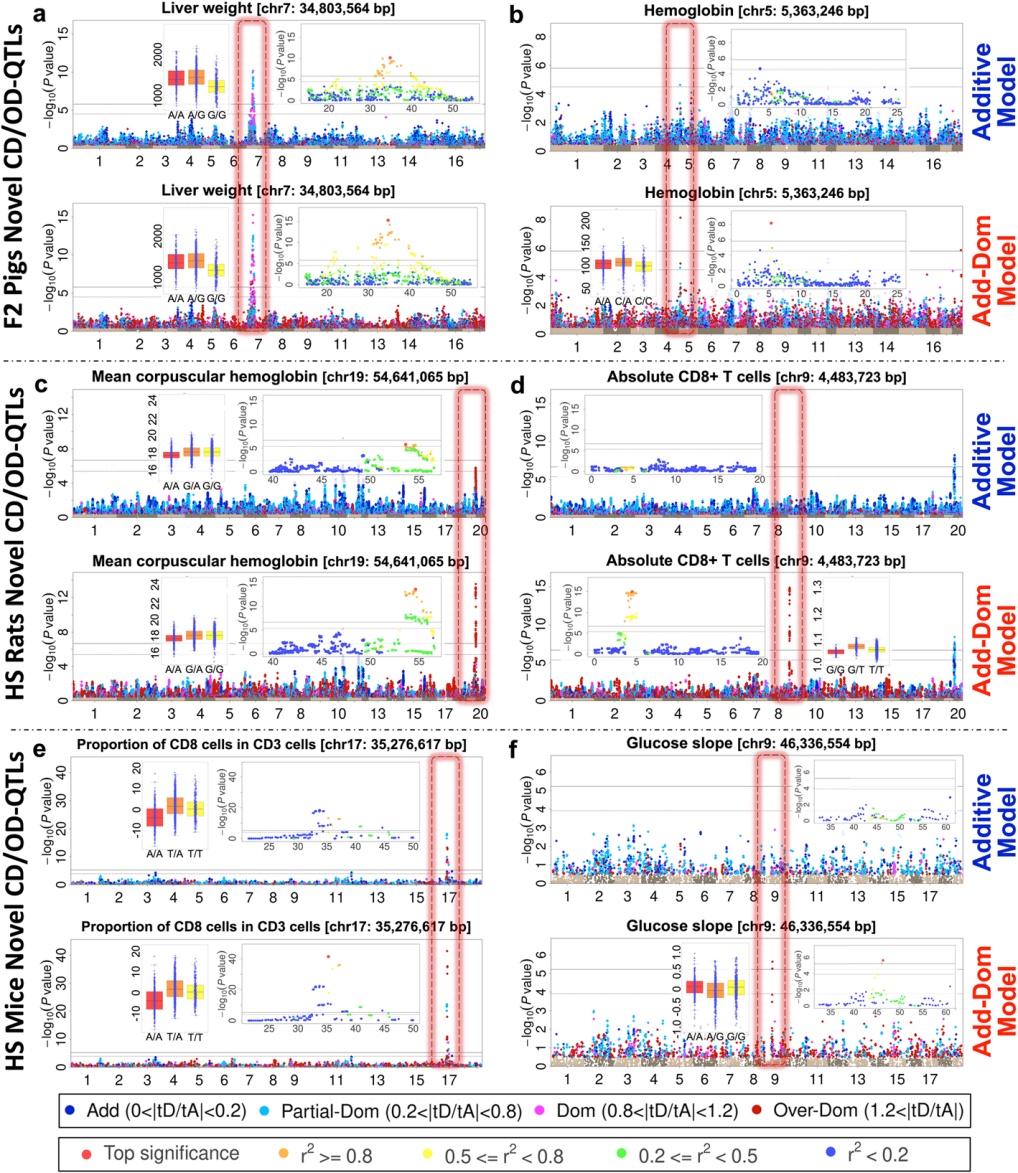
Examples of dominance QTLs. **a**, **b** Body weight and hemoglobin in F2 pigs. **c**, **d** Mean corpuscular hemoglobin and absolute CD8 + T cells in HS rats. **e**, **f** Proportion of CD3 + cells expressing CD8 + and glucose slope in HS mice. Within each part (**a**–**f**), the upper and lower panels show Manhattan plots for Add vs Add-Dom model respectively. Manhattan plot SNPs with − log_10_(*P*) > 0.5 are colored based on their ratios $\left|{t}_{Dom}/{t}_{Add}\right|$ to indicate their degree of dominance (Blue: additive, Sky blue: partial-dominant, Purple: complete-dominant, Red: over-dominant). Each panel includes insets representing the regional QTL plot and the phenotypic distribution of the peak SNP. Within each Manhattan plot, the QTL is marked by a red dotted rectangular frame, left column (**a**, **c**, **e**) for enhanced eQTLs and right column (**b**, **d**, **f**) for novel eQTLs. Color coding in regional Manhattan plots instead represents linkage disequilibrium (R^2^) with the top SNP

We show representative examples of six QTLs that are either significantly improved or only detectable by the AD model, for F2 pigs (Fig. 3a, b), HS rats (Fig. 3c, d), and HS mice (Fig. 3e, f). The dominance classifications (represented by colors in Fig. 3) of SNPs in linkage disequilibrium are generally similar. Further examples for F2 Pigs are shown in Additional file 1: Fig. S3.1 (meat quality traits), and for HS rats in Additional file 1: Fig. S3.2 (immune cell traits), and for HS mice in Additional file 1: Fig. S3.3 (immune cell traits). In the latter case, two dominance QTLs related to mouse T cell traits each localize to a potential causal gene *Bat3*, which is over 20 units of − log_10_(*P*) values more significant than the QTL found by the A-model in the neighboring gene *Myo1f*. Conditional QTL mapping analysis shows that *Bat3 and Myo1f* are associated with nearby but unlinked SNPs (Additional file 1: Fig. S3.4) and therefore represent independent effects.

For each trait, we aggregated the variances explained by the peak SNPs at all independent QTLs, partitioned into additive (${V}_{a\_QTL}$) and dominance (${V}_{d\_QTL}$) contributions (Fig. 1d–f), and compared them with the genome-wide variance components computed using GCTA (Fig. 1a–c). Each population showed the expected missing heritability, where less variance was explained by QTLs than by all SNPs.

We identified pleiotropic dominance QTL hotspots (18 in pig, 9 in rat, and 9 in mouse; Additional file 6: Table S5-1 to S5-3). In F2 pigs, chr7: 34.8 Mb-35.1 Mb is associated with many growth-related traits (e.g., ear weight, bone length, skin thickness, and carcass length), and the over-dominant hotspot chr11: 16.3–66.8 Mb is associated with many pork color traits, serum glucose level, and hematocrit. Similarly, in HS rats, the hotspot at chr9: 3.78–4.67 Mb—distinct from the rat major histocompatibility complex (MHC) locus—is associated with immunology traits. In HS mice, a hotspot in chr9: 72–111 Mb is associated with hematological traits while the MHC hotspot chr17: 33.7–37.2 Mb is associated with immunological traits.

Among traits measured on more than one species, there are six groups of potentially homologous QTLs across all three species (Additional file 7: Table S6), namely (i) ratio of CD4 + cells to CD8 + cells of rat and mouse, (ii) heart weight of pig and rat, (iii) body growth or body weight of three species, (iv) glucose tolerance of rat and mouse, (v) various serum biochemistry-related traits of rat and mouse (e.g., HDL, LDL, cholesterol, urea), and (vi) hematology traits in all three species (e.g., HCT, HGB, MCH, MCHC, MCV, PCT, RDW). The most significant examples are for MCV (Additional file 1: Fig. S4.1) and MCH (Additional file 1: Fig. S4.2). Many other examples replicate between two species (Additional file 1: Fig. S4.3). For example, within the syntenic rat and mouse MHC regions we observe dominance for many immunological traits. Dominance loci in pigs do not appear to be syntenic with those in rodents, as no F2 pig immune system traits were available for analysis. We annotated the peak SNPs of each QTL using the Variant Effect Predictor (VEP) tool [82], to predict functional consequences (Additional file 5: Table S4).

### Gene expression is strongly influenced by dominance effects

We next investigated the impact of dominance on gene expression. We evaluated seven tissues across the three populations: F2 pigs (liver and muscle), HS rats (amygdala and heart), and HS mice (hippocampus, liver, and lung), via variance decomposition with GCTA and eQTL mapping with ADDO. For rat RNA-seq data, we made separate analyses for gene and isoform expression, where a gene’s expression level is defined as the sum of all its constituent isoform levels.

**Fig. 4:**
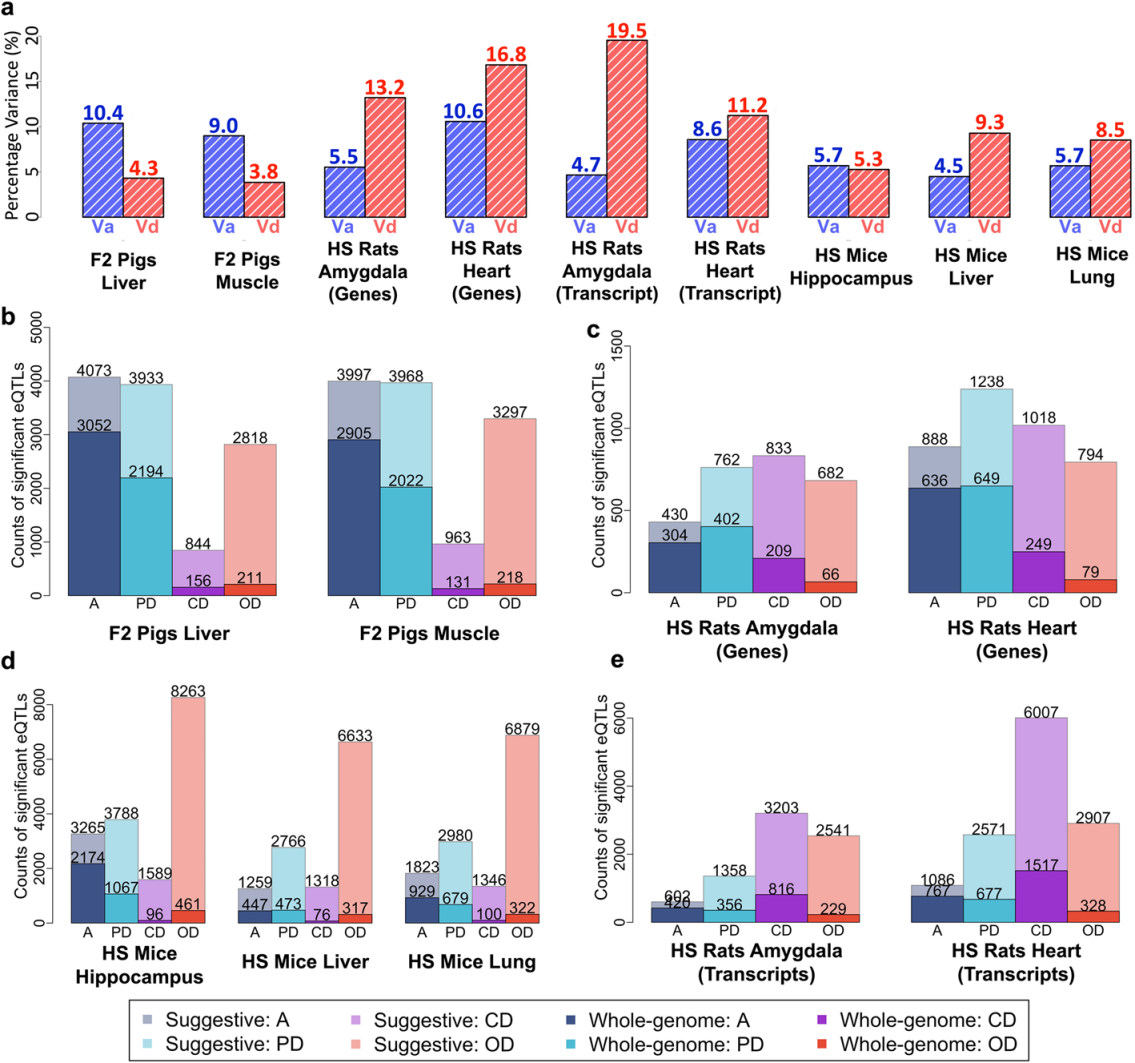
Dominance effects in gene expression. **a** Bar charts show the average heritabilities of gene expression variation for additive (${\mathrm{V}}_{\mathrm{a}}$:blue) and dominance (${\mathrm{V}}_{\mathrm{d}}:$ red) effects per species and tissue. **b**–**e** Bar charts of the numbers of additive (blue), partial-dominant (sky blue), dominant (purple), and over-dominant (red) eQTLs in different tissues in b F2 pigs (liver and muscle), **c** HS rats (gene expression of amygdala and heart), **e** HS rats (transcripts expression of amygdala and heart), and **d** HS mice (hippocampus, liver, and lung). Light shades: counts of suggestive significant eQTLs. Darker shaded bars with numbers indicate the whole genome significant eQTLs

The heritability of most expression traits was lower than for physiological traits, but surprisingly a larger fraction of the variance was accounted for by dominance, even though, as we describe below, most cis-eQTLs are additive. Figure 4a shows the averaged relative proportions of gene expression variances across species and tissues in comparison with the physiological phenotypes shown in Fig. 1d–f. In pigs, additive variance components ${V}_{a}$ were generally larger than dominance components ${V}_{d}$. Interestingly, the reverse is the case in rats and mice. Although the per-gene standard error of each estimated variance component is large, t-tests of the mean differences between ${V}_{a}$ and ${V}_{d}$ across genes are significant (*P*-value < 10^−4^).

To eliminate instabilities when both variance components are very small, we repeated the analysis restricted to genes where both ${V}_{a}>0.05$ and ${V}_{d}>0.05$ (each expression trait was first standardized to have unit variance). We observed ${V}_{d}>{V}_{a}$ in 33.6% pig, 67.8% rat, and 56.3% mouse genes (Additional file 8: Table S7). Numerous genes also exhibited very high dominance (${V}_{d}>0.15$), including 405 liver and 295 muscle genes in pig; 1651 amygdala and 9491 heart genes in rat; 887 hippocampus, 2540 liver and 2482 lung genes in mouse.

We mapped thousands of eQTLs (Additional file 9: Table S8-1, Additional file 10: Table S9-1 to S9-9 and Fig. 4b–e). We applied thresholds ranging from lax (− log_10_(*P*) = 5.8, 6.3, 5.1 for pig, rat and mouse) to stringent (− log_10_(*P*) = 8.5) to characterize the variable impact of dominance on cis and trans-eQTLs. The proportions of dominant eQTLs at different thresholds are presented in Additional file 9: Table S8-2. At lax thresholds, many trans-eQTLs are dominant; 42.3%, 81.1%, and 71.1% in pig, rat, and mouse. At more stringent thresholds—and therefore among fewer eQTLs—dominant trans-eQTLs are less common but still abundant (5.7, 61.5, and 44.7% respectively).

### Trans-acting enrichment among dominance eQTLs

We cross-tabulated additivity vs dominance against trans vs cis-eQTLs using Fisher exact tests. To simplify results, we grouped additive and partial-dominant eQTLs as “generalized additive eQTLs” (G-Add eQTL) and complete- and over-dominant eQTLs as “generalized dominance eQTLs” (G-Dom eQTL). Overall, and consistent with other studies, most cis-eQTLs are additive while trans-eQTLs are enriched for dominance effects, although it is not the case that most trans-eQTLs are dominant (Additional file 11: Table S10-1 and S10-2). Across a range of thresholds (from (− log_10_(*P*) = 5.5 to 8.5), there are statistically significant enrichments. For example, at threshold 5.5 all the *P*-values across all seven tissues are < 0.0001.

**Fig. 5:**
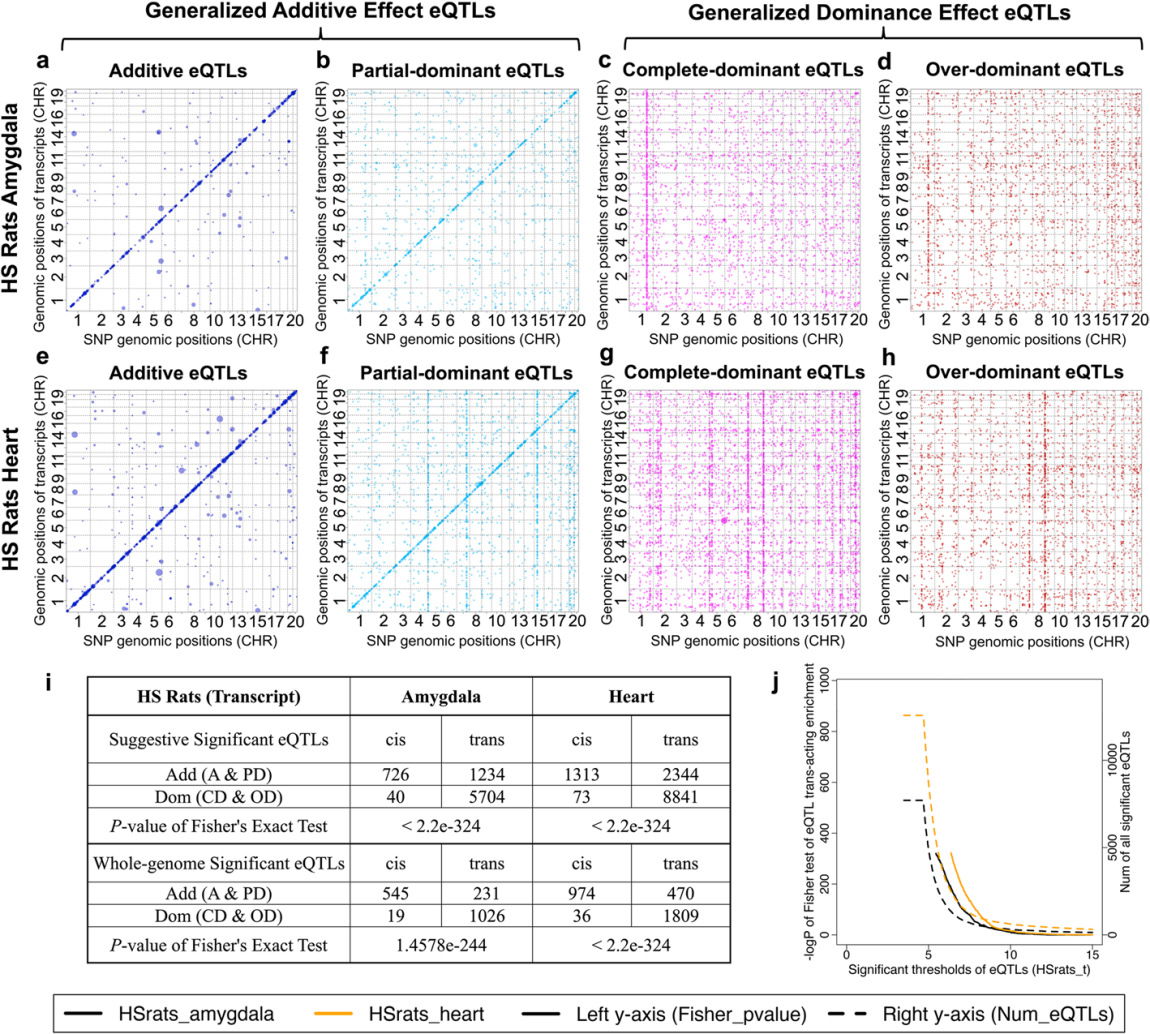
Trans-acting enrichment among dominant isoform-level eQTLs in HS rats. **a**–**h** eQTL locations of isoform-level eQTLs, filtered by dominance type. Each dot represents an eQTL significant at suggestive level (i.e., one false positive eQTL expected per isoform). *x*-axis: eQTL position, *y*-axis: physical gene location. First row **a**–**d** amygdala; Second row (**e**, **h**): heart. The four columns represent dominance types (blue: additive A, sky blue: partial-dominant PD, purple: complete-dominant CD, red: over-dominant OD). **i** Fisher’s exact test for the enrichment of trans-acting eQTLs among dominance eQTLs based on suggestive significant threshold (upper) or whole genome significant threshold (lower). **j** Dominance trans-acting enrichment (left *y*-axis, solid lines) and the counts of significant eQTLs (right *y*-axis, dashed lines) under different − log_10_(*P*) eQTL significance thresholds (*x*-axis). Enrichment is quantified by the − log_10_(*P*) values of Fisher exact test between dominance (Add/Dom-eQTLs) and regulation types (cis/trans-acting) of significant eQTLs within rat amygdala (black) and heart (orange), respectively

The genomic distributions of isoform-level HS rat eQTLs are shown in Fig. 5, and similar results for gene-level eQTLs in rats, pigs, and mice are in Additional file 1: Fig. S5.1-S5.3. We show the positions of eQTLs SNPs vs their associated isoforms (Fig. 5a–j), filtered by dominance type in HS rat amygdala and heart. There is a strong diagonal band of G-Add cis-eQTLs whereas G-Dom eQTLs are evenly distributed, not-withstanding the presence of several vertical hotspots. The phenomenon is most noticeable using lax thresholds (− log_10_(*P*) = 4.7) where eQTLs are more numerous, but it persists at more stringent thresholds (Fig. 5k, Additional file 11: Table S10-1 and S10-2). Overall, at lax thresholds, 94.8% (amygdala) and 94.7% (heart) of isoform cis-eQTLs are G-Add. In contrast, 99.3 and 99.2% dominant isoform eQTLs are trans-acting.

### eQTL hotspots are enriched for dominance effects

**Fig. 6:**
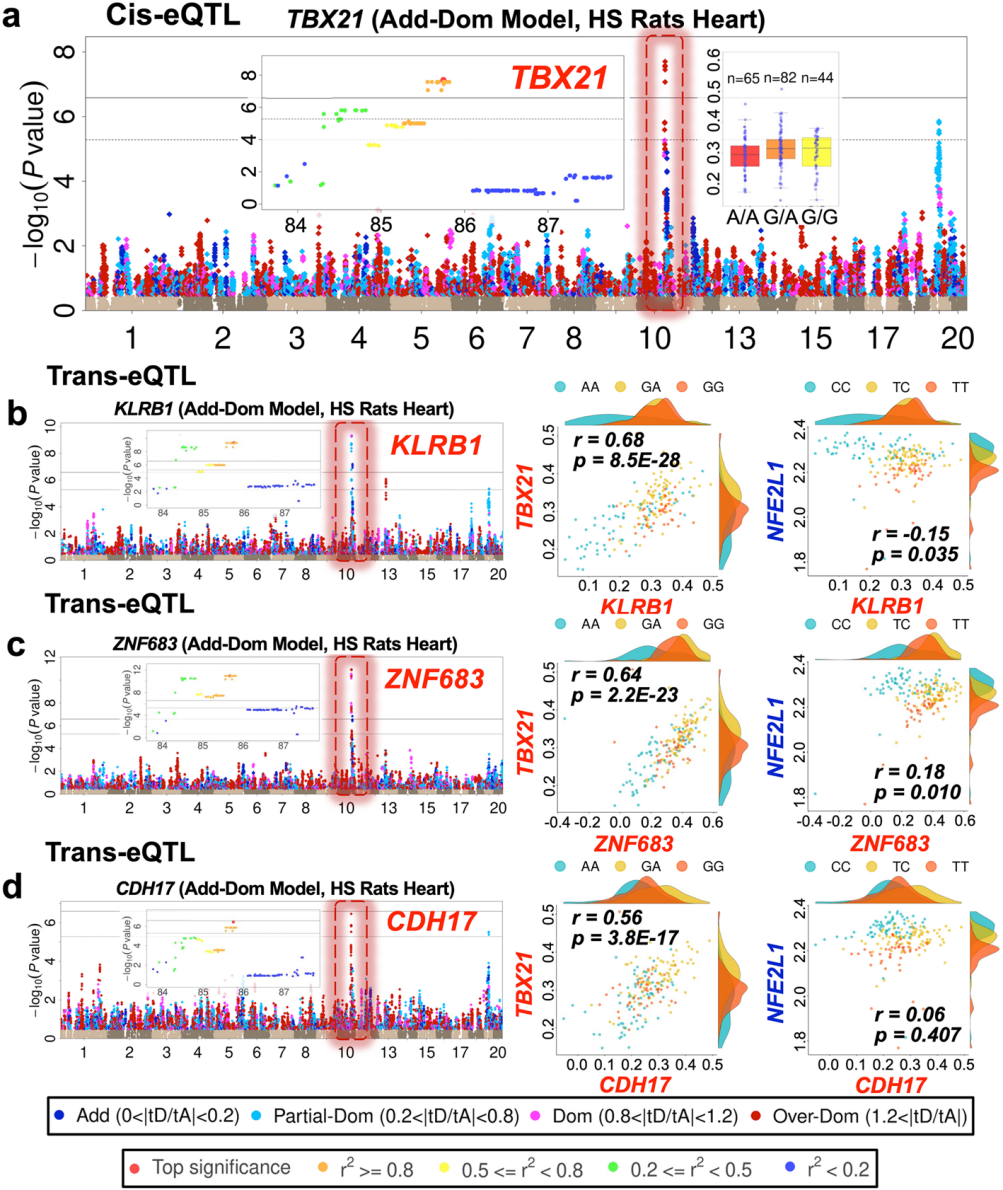
Dominant cis and trans-eQTLs at the hotspot chr10:85 Mb-86 Mb in HS rat heart. **a** Independent over-dominant cis-eQTLs within the hotspot, *Tbx21*. **b**–**d** Manhattan plots for genes *Klrb1*, *Znf683*, and *Cdh17* with over-dominant trans-eQTL mapping to *Tbx21*. Within each Manhattan plot, the eQTL is marked by a dotted rectangular frame, with the same color as the peak SNP dot (blue—additive; sky blue—partial-dominant; purple—complete-dominant; red—over-dominant), and all linked SNPs with − log10(*P*) > 0.5 are colored likewise. The regional Manhattan plots of the peak signal of each eQTL and the scatter plots of two cis-eQTLs are also shown as insets. The pairs of scatter plots to the right of each Manhattan plot compare the expression of each gene with *Tbx21* (nearby additive cis-eQTL), showing how these nearby genes are correlated with independent sets of trans-eQTLs. Each dot represents one animal, color-coded by the genotype of the peak SNP

**Fig. 7:**
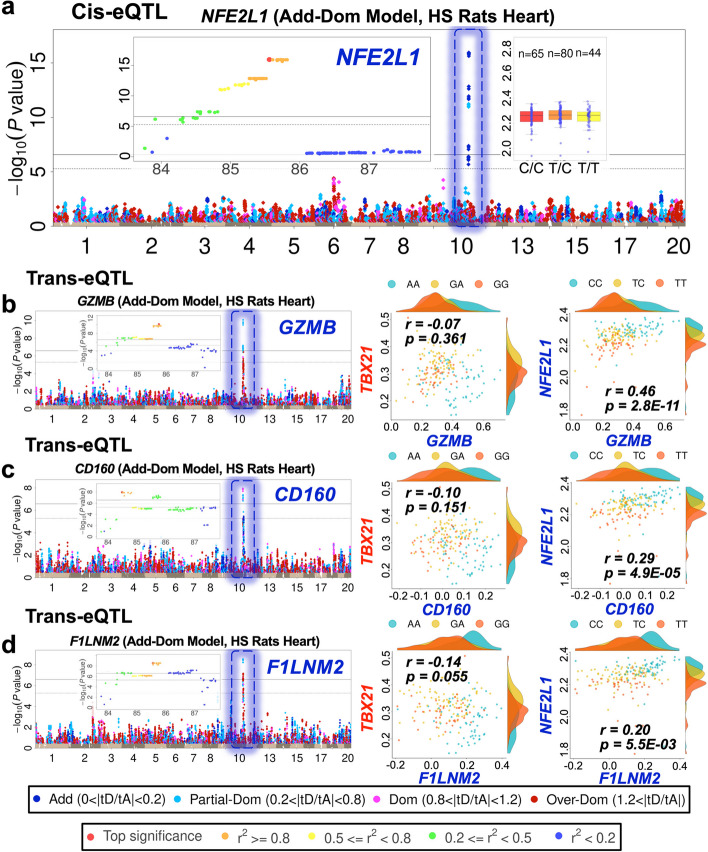
Additive cis and trans-eQTLs at the hotspot chr10:85 Mb-86 Mb in HS rat heart. **a** Independent additive cis-eQTLs within the hotspot, *Nfe2l1*. **b**–**d** Manhattan plots for genes *Gzmb*, *Cd160*, and *F1lnm2* with partial-dominant trans-eQTL mapping to *Nfe2l1*. Within each Manhattan plot, the eQTL is marked by a dotted rectangular frame, with the same color as the peak SNP dot (blue—additive; sky blue—partial-dominant; purple—complete-dominant; red—over-dominant), and all linked SNPs with − log10(*P*) > 0.5 are colored likewise. The regional Manhattan plots of the peak signal of each eQTL and the scatter plots of two cis-eQTLs are also shown as insets. The pairs of scatter plots to the right of each Manhattan plot compare the expression of each gene with *Nfe2l1* (nearby over-dominant cis-eQTL), showing how these nearby genes are correlated with independent sets of trans-eQTLs*.* Each dot represents one animal, color-coded by the genotype of the peak SNP

Trans-eQTL hotspots (i.e., where many trans-eQTL confidence intervals overlap) are ubiquitous and strongly enriched for dominance effects. For example, among suggestive significant pig eQTLs, there are 11,596 and 12,157 overlapping eQTLs that localize to just 594 and 606 separate regions in liver and muscle respectively (overlaps between eQTLs employed the 2-LOD drop method to define eQTL confidence intervals). Hotspots are summarized in Additional file 12: Table S11-1 to S11-9, where we report only high-significance eQTLs (− log_10_(*P*) > 10) but which tag most of the hotspots (2333 liver and 2088 muscle pig eQTLs from 537 and 479 hotspots). We detected a representative dominance gene-level hotspot at chr10: 85–86 Mb in HS rat heart (Figs. 6 and 7). This hotspot is complex: it has two cis-eQTLs for the transcription factors (a) *Tbx21* (over-dominant, Fig. 6) and (e) *Nfe2l1* (additive, Fig. 7) that link to six trans-eQTLs. The correlation between *Tbx21* and *Nfe2l1* expression levels is low (only − 0.11 and − 0.16 at gene and isoform level, respectively) suggesting these genes act independently. Interestingly, scatter plots of the expression of the genes underlying these trans and cis-eQTLs (Figs. 6b–d and 7b-d) suggest each cis-eQTL is associated with distinct trans-eQTLs (*Klrb1*, *Znf683*, *Cdh17* with *Tbx21, Gzmb*, *Cd160*, *F1lnm2*, with *Nfe2l1*). Additional file 1: Fig. S6.1 shows the corresponding transcript-level hotspot and Additional file 1: Fig. S6.3-S6.6 show further examples.

In addition, we detected hundreds of colocalized dominance eQTLs that regulate the same gene in different tissues within each population (tissue-consistent eQTLs, tc-eQTLs) in contrast to others that regulate different genes in different tissues (tissue-specific eQTLs, ts-eQTLs), tabulated in Additional file 13: Table S12-1 to S12-6 and summarized in Additional file 13: Table S12-7. Many hotspot-related eQTL are also present in these lists. Representative examples of cross and within-tissue eQTLs are shown in Additional file 1: Fig. S6.7.

### Dominance enrichment among genes with multiple isoforms

HS rat gene expression was measured by RNA-seq, which made it possible to distinguish expression levels of alternative isoforms, and to investigate the dominance enrichment of isoform-based eQTLs. Using the Rn4 reference annotations of 34,721 transcripts in 24,688 genes, 74.5% of genes express only one known isoform, while 22.2% (5489 genes) express two or three isoforms (Additional file 14: Table S13-2). In amygdala, we detected a higher proportion of G-Dom eQTLs among genes with multiple isoforms (ratio of G-Dom: G-Add eQTLs = 3.23, Additional file 14: Table S13-1) compared to genes with only one isoform (G-Dom: G-Add = 2.16). Chi-squared tests of enrichment were significant in both amygdala (*P* = 7.9 × 10^−11^) and heart (*P* = 5.1 × 10^−3^). Additional file 14: Tables S13-3 to S13-8 list the QTL positions for each gene with their corresponding isoforms, including 521 amygdala and 828 heart gene-based eQTLs with two or three isoforms. Additional file 14: Tables S13-9 and S13-10 show instances of antagonistic and synergistic isoform expression in amygdala and heart.

**Fig. 8:**
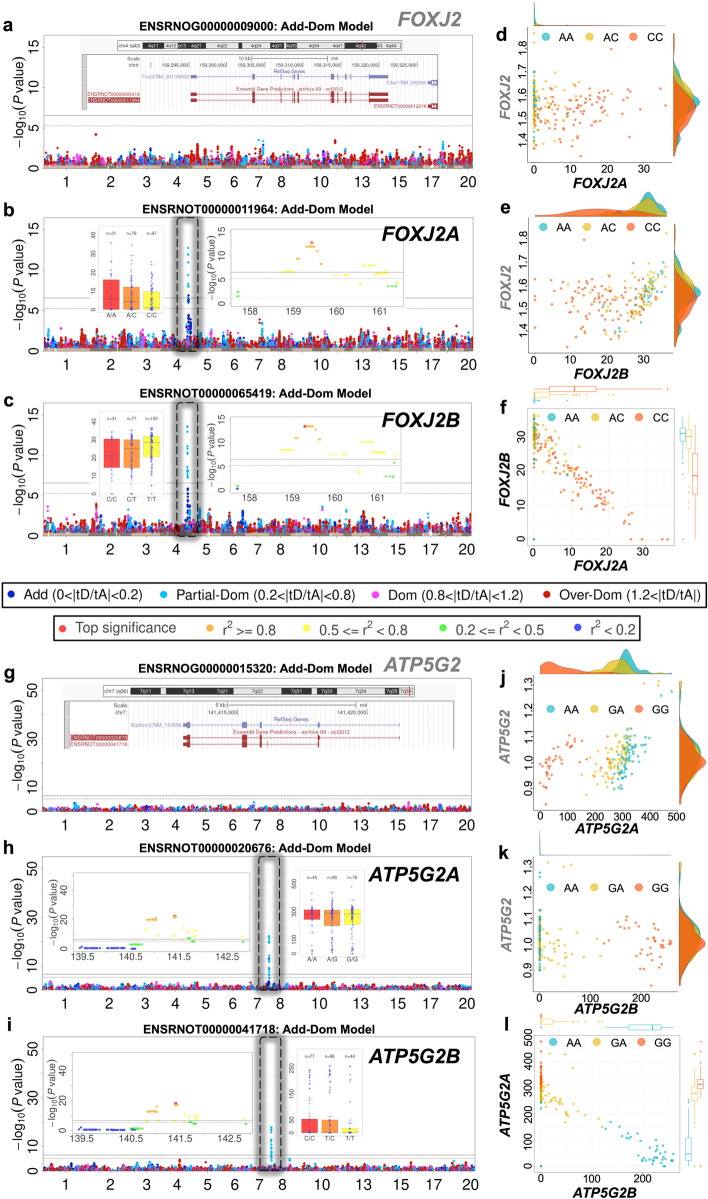
Isoform-specific antagonistic dominant eQTLs. Manhattan plots of *Foxj2* (**a–c**) and *Atp5g2* (**g–i**), based on their overall gene expression levels in HS rat amygdala, and showing no genome-wide significant eQTLs; **a** *Foxj2* (g): *Atp5g2*, with their associated isoforms’ expression levels **b** *Foxj2A*, **c** *Foxj2B*, **h** *Atp5g2A*, **i** *Atp5g2B*, and showing isoform-specific cis-eQTLs. Plot layouts are as for Fig. 3, showing Manhattan plots color-coded by dominance classification, regional QTL plots, and phenotype-genotype distribution at peak SNPs. The isoform structures for *Foxj2*, *Atp5g2* from UCSC Genome Browser are inset. d–f Scatter plots of the correlations of expression levels between *Foxj2A vs. Foxj2* (**d**), *Foxj2B vs. Foxj2* (**e**), and *Foxj2A vs. Foxj2B* (**f**). **j–l** Scatter plots of the correlations of expression levels between *Atp5g2A vs. Atp5g2* (**j**), *Atp5g2B vs. Atp5g2* (**k**), and *Atp5g2A vs. Atp5g2B* (**l**). Within each scatter plot, one dot represents one sample and the dot colors indicate the genotypes at the corresponding peak SNPs

Different isoforms of the same gene are frequently associated with different SNPs; across the 4086 rat genes with exactly two isoforms, 1257 amygdala and 1785 heart genes contain isoform-based eQTLs, but only 34 amygdala and 94 heart genes share eQTLs for both isoforms. The relative expression of different isoforms for the same gene can be either antagonistic (Fig. 8 and Additional file 1: Fig. S7.1) or synergistic (Additional file 1: Fig. S7.2). We show examples from rat amygdala of antagonistic isoforms (*Foxj2*, Fig. 8b–c; *Atp5g2*, Additional file 1: Fig S7 h-i). In both cases, overall gene expression is not heritable while each constituent isoform is under strong genetic control. Scatter plots of corresponding isoform levels (color-coded by genotype) illustrate the antagonistic effects of a SNP on different isoform from the same gene (Fig. 8d–f, j–l). We show two further antagonistic examples for *Rpl14* and *LFI44* in Additional file 1: Fig. S7.1 replicated in both rat amygdala and heart. Four examples of synergistic isoform pairs are shown in Additional file 1: Fig. S7.2, namely *Crot* and *Slc39a12* in amygdala and *Sppl2a* and *Rt1-m6-2* in heart.

Remarkably 3942 amygdala and 5659 heart genes have no gene-level eQTL but exhibit transcript-level eQTLs, and this trend increases in genes with more isoforms; across gene sets with 1/2/3 isoforms we observed respectively 13.1, 25.9, and 33.6% cases in amygdala and 20.2, 34.1, and 39.8% in heart. G-Dom eQTLs explain 82.6 and 78.8% of these cases in amygdala and heart respectively. Overall, we observed statistically significant enrichments of dominance effects in genes with multiple isoforms (*P* = 7.9 × 10^−11^ in amygdala and *P* = 0.0051 in heart; Additional file 14: Table S13-1), thereby suggesting the expression of multiple isoforms is one route by which dominance gene expression might arise.

## Discussion

Understanding the prevalence, causes, and consequences of dominance not only improves power to detect associations but also clarifies the genetic architecture of complex traits [49, 53, 83]. In this study, across a range of mammals, and phenotype classes, we have shown that dominance is common; about a quarter of the heritability of diverse physiological traits is attributable to dominance, and there are non-additive effects of various types at over half of the QTLs we mapped.

There is also an enrichment of trans-acting effects among dominance eQTLs, which has been previously reported in yeast [70, 71], plant [72], fly [73], fish [74], and mouse [75]. Further, there is enrichment for dominance among genes that express multiple isoforms. To our knowledge, this is a novel finding, although the complex genetic architecture of isoform control has also been reported in humans [84].

Dominance analysis methodologies, such as that implemented in the ADDO toolkit [19] used here, are generally applicable to the selective breeding of animals [85, 86] and plants [87, 88] and to the study of certain human diseases. For example, many Mendelian blood disorders are dominant or recessive [89]. Although dominance studies of human traits have discovered few novel associations—partly due to the ubiquity of rare alleles—there some exceptions, e.g., for age-related [53] and eye diseases [52] and for blood corpuscle measurements [56].

We suggest there are three advantages to modelling both additive and dominance genetic effects as done here. First, the strategy detects more genetic associations (Additional file 1: Fig. S2.1 and Fig. S2.2). We mapped 44.3% more associations than additive modelling alone, despite the burden of fitting additional parameters. Most of these novel QTLs are complete (68%) or over (90.9%) dominant (Additional file 1: Fig. 2). This effect is greatest in immunological and hematological traits, where some QTLs have negligible additive signal.

Second, it reveals insights into the genetic architecture of complex traits. For example, the excess of partial-dominant and over-dominant QTLs compared to complete-dominant QTLs suggests that heterosis may be caused by polygenic heterozygote advantage (the over-dominance hypothesis) rather than being driven by the superiority of a few dominant alleles over deleterious recessive alleles (the dominance hypothesis). Heterosis—which is closely related to dominance [90, 91]—is of great importance in animal improvement and was the motivation behind breeding the F2 pig population used here.

Third, integrated modelling of dominance across organismal and gene and isoform expression traits may suggest causal mechanisms. We conjecture that physiological dominance in some cases arises from the complex genetic control of alternative isoforms, as evidenced by many instances where the relationship between phenotype and genotype appears to be mediated via the expression of a particular isoform rather than by overall gene expression (Fig. 8 and Fig. S7.1; Additional file 14: Table S13). We show examples of potential causal links between gene expression and physiological traits, based on co-localisation of dominance eQTLs and QTLs, in each species (Additional file 1: Fig. S8.1 to Fig. S8.3).

More generally, what mechanisms convert genotypes into additive or dominant phenotypes? Assuming a phenotype only depends on a given genotype via expression of a gene—i.e., it does not “see” the underlying genotype—must additive/dominance gene expression necessarily cause additive/dominance phenotypes?

Considering first additive gene expression, the resulting phenotype could be either additive—if that component of the phenotype variation attributable to the expression of the gene in question is proportional to that gene’s expression level—or potentially complete-dominant if the relationship is nonlinear, for example modelling a saturation or thresholding effect. On the other hand, if gene expression is dominant in any of the senses considered here, then there is a loss of information which makes it hard or impossible to invert the nonlinear dominance relationship between genotype and expression necessary to recover additive dependence of phenotype on genotype. For example, if the gene expression exhibits complete dominance, then there is no longer any distinction between the heterozygote and one of the homozygotes.

Thus, we argue that additive gene expression eQTLs could produce either additive or dominant physiological QTLs but that dominance eQTLs should only produce dominance physiological QTLs. This seems to be an important distinction, and moreover should to all types of dominance expression, including isoform. While we cannot prove causality in the examples presented in this study, prioritizing genes by dominance allows us to exclude, for example, dominance eQTLs underlying additive physiological QTLs, which are less likely to be causal. We have shown dominance eQTLs are abundant, and hence there are many opportunities for dominance physiological QTLs to arise.

Related to this question of mechanism, we observed a clear linkage between cis vs trans-eQTLs and additive vs dominant eQTLs. Specifically, most dominant eQTLs are trans-acting, and most cis-eQTLs are additive. A potential explanation is that cis-acting causal variants tend to lie in the regulatory elements of target genes, and the degree of binding is controlled by local sequence variation, thereby causing additive changes in transcriptional levels. In contrast, causal trans-acting variants are likely to be in or near distant transcription factors (TFs) that regulate the target gene. This could lead to non-additive relationships between TF concentrations and gene expression [92]. If the two chromosomes compete for a limited supply of the TF, then non-additive expression may emerge [93]. In contrast, where a trans-eQTL behaves additively, it may control the expression of a TF which binds to both chromosomes with equal efficiency, so that expression of the target gene is proportional to the amount of TF produced, e.g., nuclear factor-κB [94]. Antagonistic pleiotropy might also explain over-dominant trans-acting eQTLs, where heterozygotes express more transcriptional outputs compared to either homozygote [95]. Additionally, long-range physical interactions between promotors and enhancers [96], and the silencing effects of some trans-eQTLs [76] could also produce transcriptional nonlinearity.

## Conclusions

In summary, incorporating dominance into quantitative genetic analysis uncovers important, complex, and interesting biology. We only have space here to highlight a few examples of dominance phenomena, but many more can be found in the supplementary tables and figures. Dominance analysis requires only a slightly different workflow compared to additive analysis. Hence, even though the greater part of trait heritability is typically additive, there are few if any disadvantages to searching for dominance and we recommend its routine use.

## Methods

### Data processing of genotypes, physiological and expression traits in F2 pigs

The F2 pigs were established by crossing Chinese Erhualian and White Duroc pigs in 1998, as described [97]. Genotypes of 1005 F2 pigs were measured using the porcine SNP60 Beadchip (Illumina) at 62,613 SNP sites, which were filtered by minor allele frequency (MAF) < 0.05, missing rate > 0.1, and minimum frequency of the rarest genotype at each locus > 10, to leave 39,298 SNPs for downstream analysis. A total of 253 complex traits were measured for growth, fatness, meat quality, basal hematology, and serum biochemistry. For each trait, we controlled for outliers by removing values more than 5 s.d. from the mean. Gene expression from 493 liver and 583 muscle samples of the same pigs was measured using digital gene expression (DGE), processed into transcript per million (TPM) values for 15,684 and 17,822 transcripts in liver and muscle respectively. The TPM values were quantile normalized and also adjusted for sex, batch, and the first ten principal components of expression data.

### Data processing of genotypes, organismal, and expressional traits in HS rats

The HS rats were descended from eight inbred strains (ACI/N, BN/SsN, BUF/N, F344/N, M520/N, MR/N, WKY/N, and WN/N) [98], by rotational breeding over many generations, such that each HS rat chromosome is a mosaic of the founder genomes.

In total, the genotypes of 1407 individuals [78] were measured by a custom Affymetrix array for 257,868 SNPs, as well as a comprehensive measurement of 220 complex traits, including various complex traits related to psychology, basal hematology, basal immunology, and serum biochemistry. Transcriptome RNA sequencing (RNA-seq) for 205 amygdala and 192 heart previously unpublished samples from the same animals were analyzed in this study. We used different pipelines to quantify gene and transcript expression levels, using the same reference (*Rattus norvegicus* genome, Ensembl RGSC3.4) for consistency with the version used for the array genotypes. For the gene expression levels, we first aligned clean reads to the rat RGSC3.4 reference genome using STAR (v.2.5.3a), and removed duplicated reads by Picard (v.2.5.0). Next, we estimated the raw read counts of each gene using featureCounts (v.1.5.2), and normalized the counts using the Trimmed Mean of M-value (TMM) method, implemented in edgeR (v.3.20.9). For the transcript levels, we used Kallisto (v.0.43.1) to estimate the transcript per million (TPM) of all transcripts in rat RGSC3.4 genome, followed by the quantile normal transformation before GWAS analysis.

### Data processing of genotypes, organismal, and expressional traits in HS mice

The HS mice originated from eight inbred progenitors (A/J, AKR/J, BALBc/J, CBA/J, C3H/HeJ, C57BL/6 J, DBA/2 J, and LP/J) [99] with a similar design as for HS rats. In total, the genotypes of 2002 individuals were measured by a custom Illumina assay for 10,168 SNPs [79]. In total, 125 traits were measured as described in [100], including basal immunological, basal hematology, and models of human disease related to anxiety, asthma, diabetes, and obesity. Gene expression data (371 hippocampus samples, 227 liver samples, and 197 lung samples) were also measured using Illumina microarray-based assays [81].

### Estimation of additive and dominance variance components

We estimated additive and dominance variance components and heritabilities using GCTA [65] in a standard workflow: We first corrected the raw phenotypes regressing out covariates using lm() function in R and then standardized the residuals. We generated ${{\varvec{K}}}_{{\varvec{a}}},\boldsymbol{ }{{\varvec{K}}}_{{\varvec{d}}}$ the additive and dominant genetic relationship matrices (GRMs) of all individuals by GCTA. Pairs of individuals with absolute additive genetic correlation > 0.7 were randomly downsampled to single individuals, and the GRMs rebuilt using the remaining individuals. Finally, we calculated the variance components for additive and dominance effects using GCTA.

### Detection and classification of QTLs and eQTLs

All QTL and eQTL mapping was done using the ADDO [19] toolkit. We fitted three mixed models, namely the Add-Dom (AD), Add (A), and Dom (D) models to detect, classify, and compare additive and dominance QTLs. Mixed models correct for unequal relatedness between individuals and to avoid false positive QTL calls. In brief, we model the phenotypic variance covariance matrix ${\varvec{V}}={{\varvec{K}}}_{{\varvec{a}}}{\sigma }_{a}^{2}+{{\varvec{K}}}_{{\varvec{d}}}{\sigma }_{d}^{2}+{\varvec{I}}{\sigma }_{e}^{2}$ where ${\sigma }_{a}^{2},{\sigma }_{d}^{2},{\sigma }_{e}^{2}$ are the additive, dominant, and environmental variance components estimated by GCTA. We multiply the phenotype vector and fixed effects design matrix by the matrix ${{\varvec{V}}}^{-0.5}$ to convert the mixed model to ordinary least squares with iid errors. To test association as a specific SNP with genotypes AA/AB/BB, we consider the following linear model fixed effect design matrices:

1. “Add (A) Model”: The three genotypes are recoded as 0/1/2 within each locus, i.e., as additive genotype dosages, in order to model additive genetic effects. The design matrix is thus a column of ones (for the intercept) and a column of genotype dosages. Except that the variance matrix incorporates dominance effects, the A model is equivalent to the usual additive model used in mixed model GWAS.
2. “Dom (D) Model”: Genotypes AA/AB/BB are coded as heterozygote dosages 0/1/0, to detect loci where the effect of heterozygote AB is from the mean effect of two homozygotes AA and BB. The design matrix is a column of ones and a column of heterozygote dosages.
3. “Add-Dom (AD) Model”: Genotypes are coded as two columns 0/1/2 (additive dosage) and 0/1/0 (heterozygote dosage). The design matrix is a column of ones and both of these columns, and which can model any type of dominance effect by suitable choice of the regression coefficients ${\beta }_{a},{\beta }_{d}$ corresponding to the additive and dominance columns respectively. We computed the T-statistics ${t}_{Add},{t}_{Dom}$ for these coefficients by dividing each by their estimated standard errors.
4. “AvsAD Model”: We used ANOVA to compare the A Model and AD Model, to detect loci with significant non-additive effect. Statistical significance was reported as the negative base 10 log *p*-value of the ANOVA comparison of the models.

We used two *p*-value thresholds to report significant QTLs: (1) a suggestive threshold (1/N_SNP_), where N_SNP_ is the number of SNPs. Under the null hypothesis where no SNPs are associated, one false positive is expected per genome scan; (2) whole genome-wide significance (0.05/N_SNP_), in order to control for false positives caused by multiple tests, and where a false positive should occur once in every 20 GWAS. The width of each QTL was determined using 2 point LOD drop from the peak SNP at the QTL.

We classified each QTL into a dominance type (A: additive/PD: partial-dominant/CD: complete-dominant/OD: over-dominant), based on the log ratio of T-statistics from the Add-Dom Model, i.e., $r={\mathrm{log}}_{2}|{t}_{Dom}/{t}_{Add}|$, then all the significant QTLs could be classified into four groups using the rules A: r < 0.2, PD: 0.2 < r < 0.8, CD: 0.8 < r < 1.2 or OD: r > 1.2. For greater clarity we plotted ratios as log_2_(r).

eQTLs were classified as cis-acting if the eQTL localized to the same chromosome with its target gene and the minimum of left and right boundary distances between “Peak SNP” and “gene physical range” < 2 Mb; otherwise, it was classified as trans-acting.

### Supplementary Information

**Additional file 1: Fig. S1.1.** Examples of dominance classifications visualized with different coordinate systems for QTL category comparison. **Fig. S1.2.** Additive (blue bars, ) and Dominance (red bars, ) Variance Components of all traits across three populations. **Fig. S2.1.** QTLs detected by AD model. **Fig. S2.2.** Comparison of QTL detection among A, D and AD models. **Fig. S3.1.** Over-dominant QTLs in F2 pigs. **Fig. S3.2.** Over-dominant HS rat QTLs. **Fig. S3.3.** Over-dominant QTLs implicate a novel causal gene regulating the proportions of CD4+ and CD8+ T cells in HS mice, compared to additive QTL modelling. **Fig. S3.4.** Conditional GWAS of the proportion of CD4+ cells in CD3+ cells in HS mice. **Fig. S4.1.** Homologous QTLs for mean corpuscular volume (MCV). **Fig. S4.2.** Homologous QTLs for mean corpuscular hemoglobin (MCV). **Fig. S4.3.** Homologous QTLs. **Fig. S5.1.** Trans-acting enrichment among dominant eQTLs in liver and muscle in F2 pigs. **Fig. S5.2.** Trans-acting enrichment among dominant eQTLs of amygdala and heart tissues in HS rats. **Fig. S5.3.** Trans-acting enrichment among dominant eQTLs of hippocampus, liver and lung tissues in HS mice. **Fig. S6.1.** Dominant cis and trans transcript eQTLs at the hotspot chr10:85Mb-86Mb in HS rat heart. **Fig. S6.2.** Interplay of immunology-related QTLs in HS rats. **Fig. S6.3.** Dominant eQTLs detected by AD model from two trans-acting hotspots (a1-a7, chr3: 147Mb-149Mb; c1-c7, chr7: 107Mb-108Mb) in HS rat heart. **Fig. S6.4.** Dominant eQTLs from a trans-acting hotspot in HS mice hippocampus. **Fig. S6-5.** Dominant eQTLs from a trans-acting hotspot located at chromosome 7 in HS mice lung. **Fig. S6.6.** Dominant eQTLs from a trans-acting hotspot located at chromosome 9 in HS mice lung. **Fig. S6.7.** Tissue-conserved and tissue-specific dominant eQTLs. **Fig. S7.1.** Transcript-specific antagonistic dominant eQTLs of *RPL14* and *LFI44* in HS rat amygdala and heart. **Fig. S7.2.** Transcript-specific synergistic eQTLs in HS rats.**Fig. S8.1.** A pleiotropic over-dominant pig QTL for kidney weight and small intestinal length colocalized with *MTCH1* eQTL in F2 pigs. **Fig. S8.2.** Dominant QTLs for three rat hematology traits colocalized with cis/trans eQTLs in rat amygdala and heart (chr19: 53 Mb-55 Mb). **Fig. S8.3.** An over-dominant QTL for mouse glucose slope colocalized with eQTLs in mouse amygdala, liver and lung.

**Additional file 2: Table S1-1.** Summary of datasets in F2 pigs, HS rats and HS mice, together with phenotype and genotype quality control methods applied in QTL/eQTL mapping; **Table S1-2.** Trait classes and total numbers.

**Additional file 3: Table S2. **Phenotypic variances with standard errors, due to additive and dominance effects (calculated by genome-wide SNP heritability or from accumulated significant QTLs) of 242 physical traits in F2 pigs (Table S2-1), 206 physical traits of HS rats (Table S2-2) and 124 physical traits of HS mice (Table S2-3), together with average values across each trait class.

**Additional file 4: Table S3. **Counts and proportions of significant QTLs under dominance types within each population, and the corresponding missing rate compared to the additive GWAS, under suggestive thresholds and whole genome thresholds. 

**Additional file 5: Table S4.** Descriptive statistics, effect classification and consequence annotation of all significant QTL detected by AD Model in F2 pigs (Table S4-1), HS rats (Table S4-2) and HS mice (Table S4-3). (Rows with -log_10_(*P*)values of ADvsA Model > 5 are in red, rows where -log_10_(*P*)values of AD Model > 6 are in blue.).

**Additional file 6: Table S5.** Summary of novel pleiotropic QTLs detected by AD model within each population. (Different shared QTL regions are separated by graey background and the rows with red numbers indicate novel QTLs where -log_10_(*P*)values of ADvsA Model > 5).

**Additional file 7: Table S6.** Summary of homologous QTLs of common physical traits across three stocks. (Different trait groups are separated by frame and QTL rows of different stocks are colored differently).

**Additional file 8: Table S7.** Gene expression heritabilities and standard errors explained by additive and dominance effects estimated by genome-wide SNPs within each tissue of F2 pigs (Table S7-1), HS rats (Table S7-2) and HS mice (Table S7-3), summary of average additive and dominance variance components of each tissue across three populations (Table S7-4).

**Additional file 9: Table S8.** Counts and proportions of significant eQTLs classified by dominance effect type within each tissue and each population under different significant thresholds (Table S8-1); Proportions of novel eQTLs detected by AD model compared to A model in cis/trans groups (Table S8-2).

**Additional file 10: Table S9.** Classification of eQTLs detected by AD Model (Table S9-1, F2 pigs liver tissue; Table S9-2, F2 pigs muscle tissue; Table S9-3, HS rats amygdala genes expression; Table S9-4, HS rats heart genes expression; Table S9-5, amygdala transcripts expression; Table S9-6, heart transcripts expression; Table S9-7, HS mice hippocampus gene expression; Table S9-8, HS mice liver gene expression; Table S9-9, HS mice lung gene expression). Rows with -log_10_(*P*)values of ADvsA Model > 6 are in red, remaining rows with -log_10_(*P*)values of AD Model> 10 are in blue.

**Additional file 11: Table S10.** Counts of significant cis-eQTLs and trans-eQTLs under four different effect types within each tissue of three populations under different significant thresholds, together with the correlation test between different genetic modes and different regulatory types (Table S10-1, A/PD/CD/OD vs cis/trans; Table S10-2, A & PD/CD & OD vs cis/trans, using Add-eQTL represents A- & PD-eQTL and Dom-eQTL represents CD- & OD-eQTL).

**Additional file 12: Table S11.** Summary of pleiotropic eQTLs (Table S11-1, F2 pigs liver genes expression; Table S11-2, F2 pigs muscle genes expression; Table S11-3, HS rats amygdala genes expression; Table S11-4, HS rats heart genes expression; Table S11-5, amygdala transcripts expression; Table S11-6, heart transcripts expression; Table S11-7, HS mice hippocampus gene expression; Table S11-8, HS mice liver gene expression; Table S11-9, HS mice lung gene expression). Lines in bold indicate cis-eQTLs.

**Additional file 13: Table S12.** Summary of eQTLs shared across tissues within each population (Table S12-1, liver-muscle in F2 pigs; Table S12-2, amygdala-heart gene expression levels in HS rats; Table S12-3, amygdala-heart transcript expression levels in HS rats; Table S12-4, hippocampus-liver in HS mice; Table S12-5, hippocampus-lung in HS mice; Table S12-6, liver-lung in HS mice). All rows are ordered by (1) whether from the same gene (2) the -log_10_(*P*)values of ADvsA Model. Table S12-7, counts of shared eQTLs between different tissues within each specie.

**Additional file 14: Table S13.** Counts of significant eQTLs classified by transcript compositions (one transcript or multi transcripts) and by dominance type, in HS rats heart and amygdala, together with the correlation test between dominance type (A/PD/CD/OD) and transcript compositions.

**Additional file 15.** Review history.

## Data Availability

The rat RNA-seq data have been deposited at ENA Biostudies with the accession number PRJEB60349 [101] and PRJEB60407 [102]. All other data used in this study are from previously published studies (pig [81, 103], rat [78], mouse [79, 81, 104]) and available as described. All the codes used for variance decomposition, QTL mapping, RNA-seq, eQTL mapping, and variant annotation as well as the respective quality control of raw phenotype and genotype, results summary, comparison, and visualization scripts are available under the GPL-3.0 license on Github [105] and on Zenodo [106]. The authors declare all scripts and software used in this paper are mentioned in the Methods section.
