# Supplementary material for: Dominance is common in mammals and is associated with trans-acting gene expression and alternative splicing
Source: Genome Biol. 2023 Sep 29;24:215. doi: 10.1186/s13059-023-03060-2 (PMC10540365; doi:10.1186/s13059-023-03060-2)
Supplement: Supplementary file 1 — Additional file 1: Fig. S1.1. Examples of dominance classifications visualized with different coordinate systems for QTL category comparison. Fig. S1.2. Additive (blue bars, ) and Dominance (red bars, ) Variance Components of all traits across three populations. Fig. S2.1. QTLs detected by AD model. Fig. S2.2. Comparison of QTL detection among A, D and AD models. Fig. S3.1. Over-dominant QTLs in F2 pigs. Fig. S3.2. Over-dominant HS rat QTLs. Fig. S3.3. Over-dominant QTLs implicate a novel causal gene regulating the proportions of CD4+ and CD8+ T cells in HS mice, compared to additive QTL modelling. Fig. S3.4. Conditional GWAS of the proportion of CD4+ cells in CD3+ cells in HS mice. Fig. S4.1. Homologous QTLs for mean corpuscular volume (MCV). Fig. S4.2. Homologous QTLs for mean corpuscular hemoglobin (MCV). Fig. S4.3. Homologous QTLs. Fig. S5.1. Trans-acting enrichment among dominant eQTLs in liver and muscle in F2 pigs. Fig. S5.2. Trans-acting enrichment among dominant eQTLs of amygdala and heart tissues in HS rats. Fig. S5.3. Trans-acting enrichment among dominant eQTLs of hippocampus, liver and lung tissues in HS mice. Fig. S6.1. Dominant cis and trans transcript eQTLs at the hotspot chr10:85Mb-86Mb in HS rat heart. Fig. S6.2. Interplay of immunology-related QTLs in HS rats. Fig. S6.3. Dominant eQTLs detected by AD model from two trans-acting hotspots (a1-a7, chr3: 147Mb-149Mb; c1-c7, chr7: 107Mb-108Mb) in HS rat heart. Fig. S6.4. Dominant eQTLs from a trans-acting hotspot in HS mice hippocampus. Fig. S6-5. Dominant eQTLs from a trans-acting hotspot located at chromosome 7 in HS mice lung. Fig. S6.6. Dominant eQTLs from a trans-acting hotspot located at chromosome 9 in HS mice lung. Fig. S6.7. Tissue-conserved and tissue-specific dominant eQTLs. Fig. S7.1. Transcript-specific antagonistic dominant eQTLs of RPL14 and LFI44 in HS rat amygdala and heart. Fig. S7.2. Transcript-specific synergistic eQTLs in HS rats.Fig. S8.1. A pleiotropic over-dominant pig QTL for k [file 13059_2023_3060_MOESM1_ESM.pdf]

Supplementary Materials for

Dominance is common in mammals and is associated with trans-acting gene expression and alternative splicing

Leilei Cui[1,2,3,4†], Bin Yang[1†], Shijun Xiao[1], Jun Gao[1], Amelie Baud[5], Delyth Graham[6], Martin McBride[6], Anna Dominiczak[6], Sebastian Schafer[7], Regina Lopez Aumatell[8], Carme Mont[9], Albert Fernandez Teruel[10], Norbert Hübner[11,12,13], Jonathan Flint[14], Richard Mott[2*] and Lusheng Huang[1*]

[1]National Key Laboratory for Pig Genetic Improvement and Production Technology, Jiangxi Agricultural University, Nanchang, China. [2]UCL Genetics Institute, University College London, London, UK. [3]Human Aging Research Institute and School of Life Science, Nanchang University, and Jiangxi Key Laboratory of Human Aging, Jiangxi, China. [4]School of Life Sciences, Nanchang University, Nanchang, China. [5]Centre for Genomic Regulation (CRG), The Barcelona Institute of Science and Technology, Barcelona, Spain. [6]BHF Glasgow Cardiovascular Research Centre, University of Glasgow, Glasgow G12 8TA UK. [7]Cardiovascular and Metabolic Disorders Program, Duke-National University of Singapore Medical School, Singapore, [8]Department of Medicine and Life Sciences, Universitat Pompeu Fabra, Barcelona, Spain. [9]Wellcome Trust Centre for Human Genetics, University of Oxford, Oxford UK. [10] Departamento de Psiquiatría y Medicina Legal, Universitat Autonoma de Barcelona, Spain. [11]Genetics and Genomics of Cardiovascular Diseases Research Group, Max Delbrück Center (MDC) for Molecular Medicine in the Helmholtz Association, Berlin, Germany. [12]DZHK (German Center for Cardiovascular Research) partner site Berlin, Berlin, Germany. [13]Charité Universitätsmedizin Berlin, Berlin, Germany. [14]Department of Psychiatry and Behavioral Sciences, Brain Research Institute, University of California, Los Angeles, Los Angeles, CA, USA.

Address for correspondence

Lusheng Huang: Lushenghuang@hotmail.com,

National Key Laboratory for Pig Genetic Improvement and Production Technology, Jiangxi Agricultural University, Nanchang, 330045, P. R. China

Richard Mott: r.mott@ucl.ac.uk

UCL Genetics Institute, University College London, London, WC1E 6BT, UK

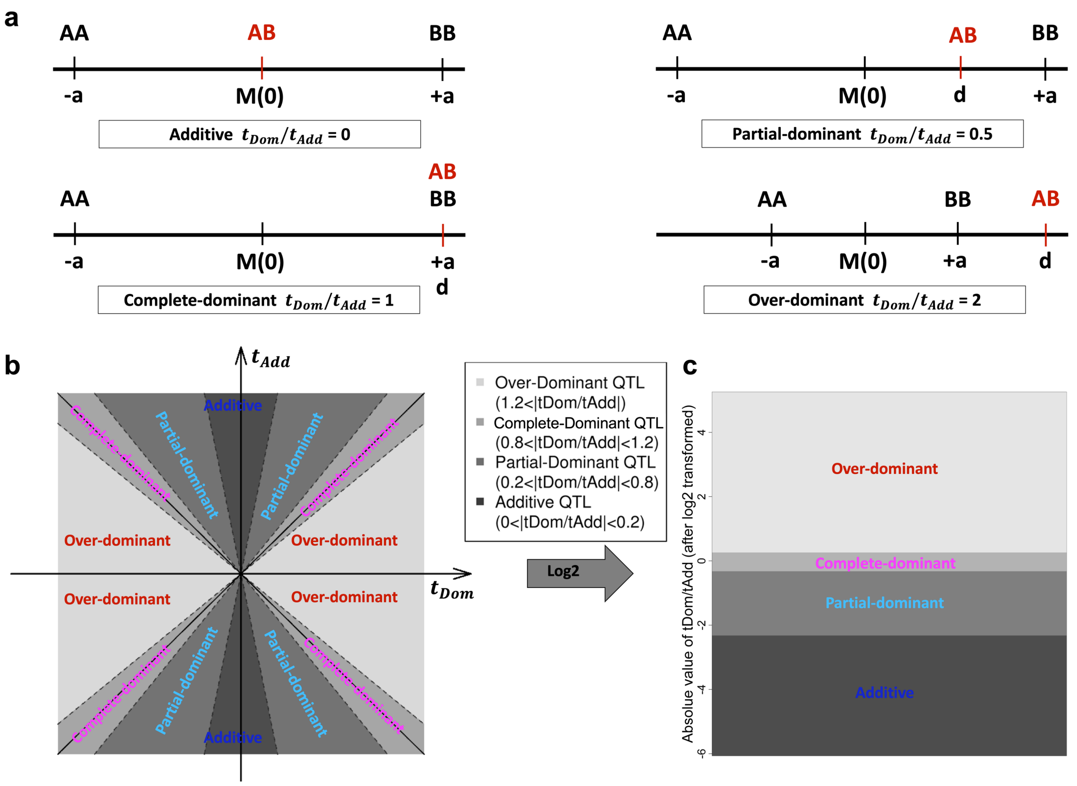

Fig. S1.1 Examples of dominance classifications visualized with different coordinate systems for QTL category comparison. (a) Diagrams illustrating dominance classifications in terms of the phenotypic values associated with each genotype class (AA, AB, BB) and in terms of the additive (a) and dominance (d) deviations from the mid-parent value M(0). (b) and (c) The radiated and longitudinal coordinate systems for QTL display ((c) is log2 of (b)).

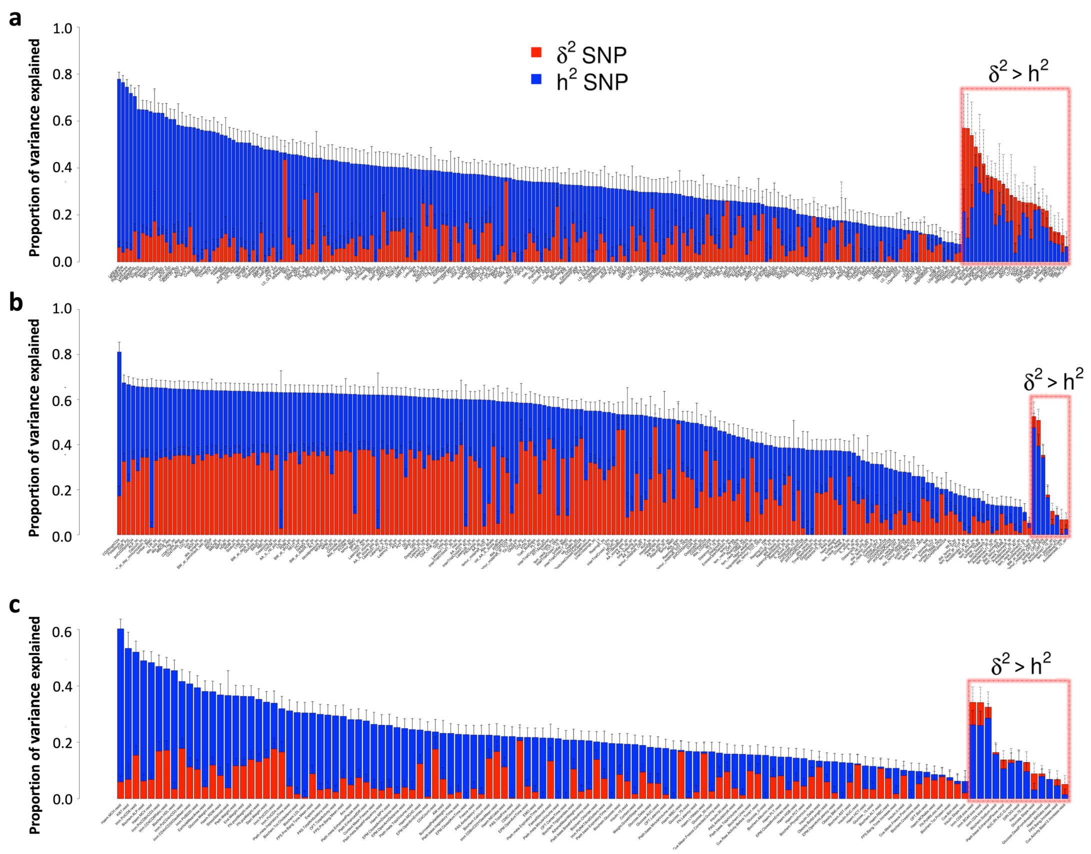

Fig. S1.2 Additive (blue bars, h^2_{SNP}) and Dominance (red bars, δ^2_{SNP}) Variance Components of all traits across three populations. (a): F2 pigs, (b): HS rats, (c): HS mice. Each vertical bar represents one trait, labelled on x-axis. Height of each bar represents the total heritability of the corresponding trait, partitioned into additive (blue) and dominance (red) components, as estimated by GCTA. The solid and dashed error bars denote the standard errors of additive and dominance effects in each trait, respectively. Red dashed boxes highlight traits with higher δ^2_{SNP} than h^2_{SNP}, for which are sorted by δ^2_{SNP} in the red boxes, and other traits are sorted by h^2_{SNP} from left to right.

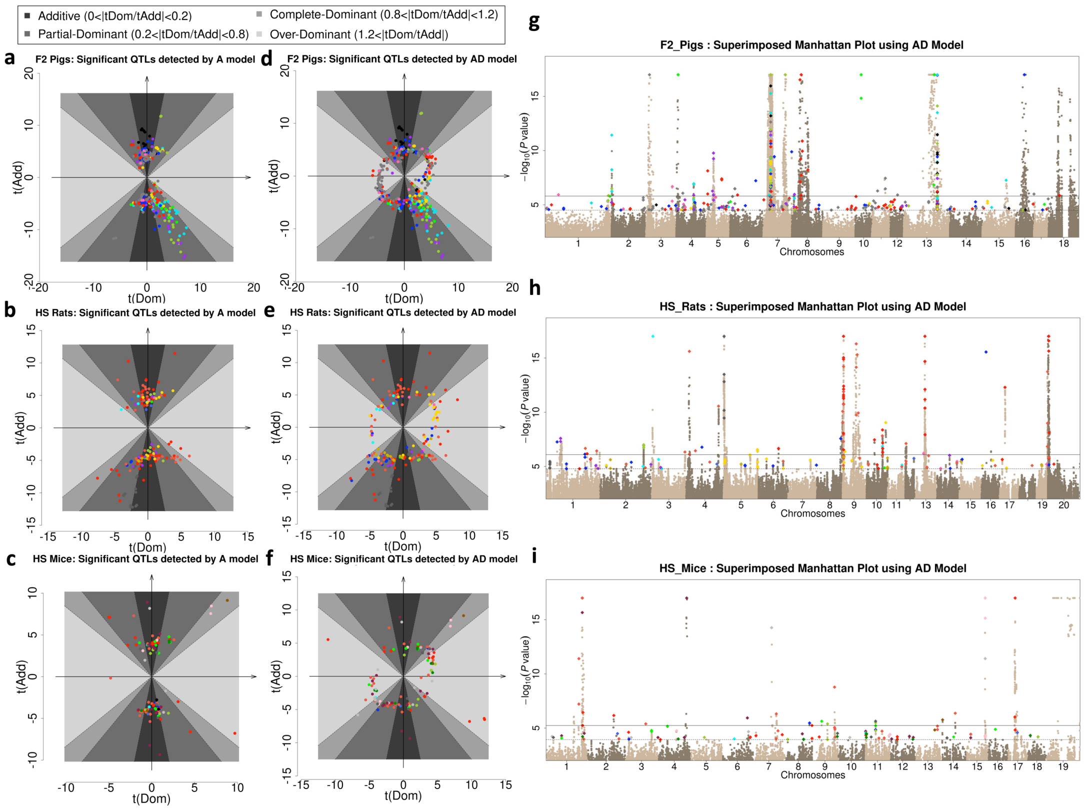

Fig. S2.1 QTLs detected by AD model. (a-f) show the same data as in Fig 2 where QTLs are classified according to the ratios of their T-statistics from the AD-model, except that in this figure the ratios have not been log-transformed. Each row is one population (a,b: F2 pigs; c,d: HS rats; e,f: HS mice). (a-c): QTLs detected by A-model. (d-f): QTLs detected by AD-model. Within each panel, the x-axis and y-axis represent t_{Dom} and t_{Add}, the standardized dominance (β_{Dom}) and additive effects (β_{Add}), respectively. Each dot is one trait, colour-coded by trait type using the same coding as in Fig 2. The dot localizations represent different inheritance models (from deep grey to light grey are additive, partial-dominant, complete-dominant and over-dominant). (g-i) Aggregate Manhattan plots of the AD GWAS of all physiological traits in pig (g), rat (h) and mouse (i). Associations with -log(P) > 18 are truncated and data points with -log(P) < 2 are not shown. The same coloring scheme is used for the peak QTL of each trait, the solid and dashed horizontal line represents the average suggestive and whole-genome significant thresholds across all traits.

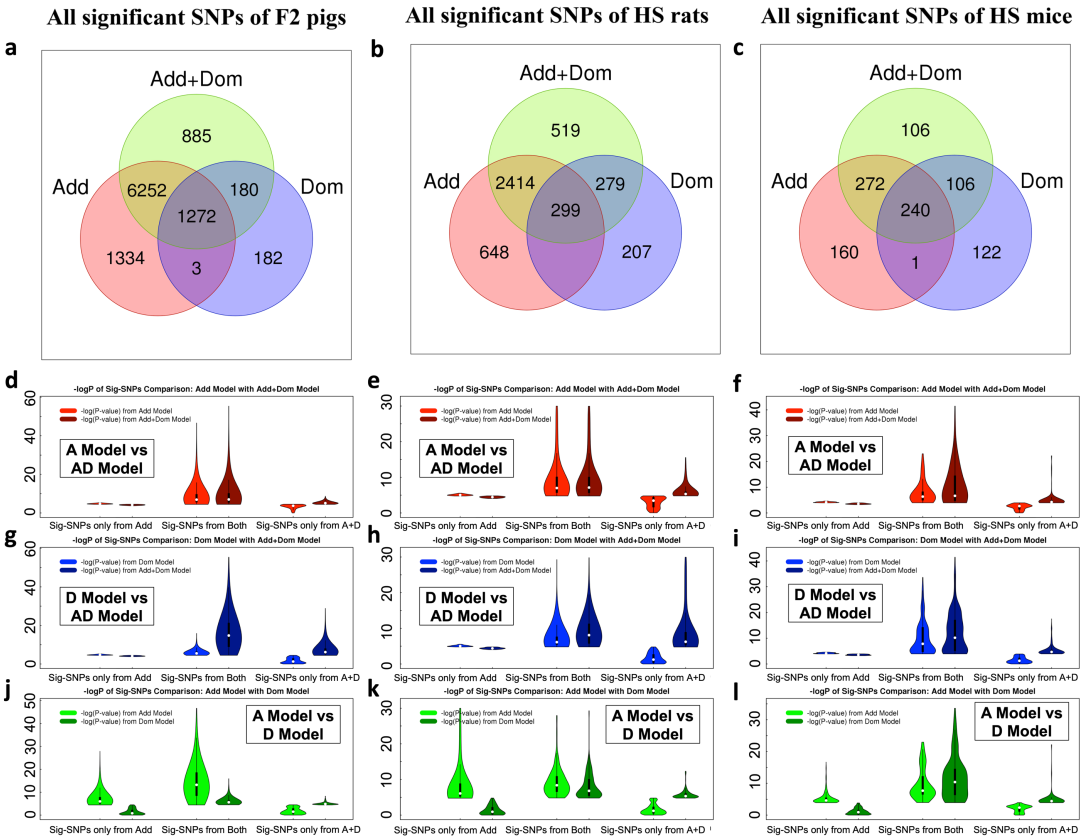

Fig. S2.2 Comparison of QTL detection among A, D and AD models. (a-c) Venn diagrams showing overlapping significant SNPs detected by A model (red), D model (blue) and AD model (green) across 247 traits of F2 pigs (a), 207 traits of HS rats (b) and 124 traits of HS mice (c). (d-f) Violin plots showing the distributions of -log(*P*) values of A model and AD model only using SNPs detected by A model (left), SNPs detected by both A and AD models (middle) and SNPs only detected by D model (right) in pig (d), rat (e) and mouse (f), respectively. (g-i) Similar violin plots comparing SNPs detected by D and AD models in pig (g), rat (h) and mouse (i). (j-l) Similar violin plots comparing SNPs detected by A and D models in pig (j), rat (k) and mouse (l).

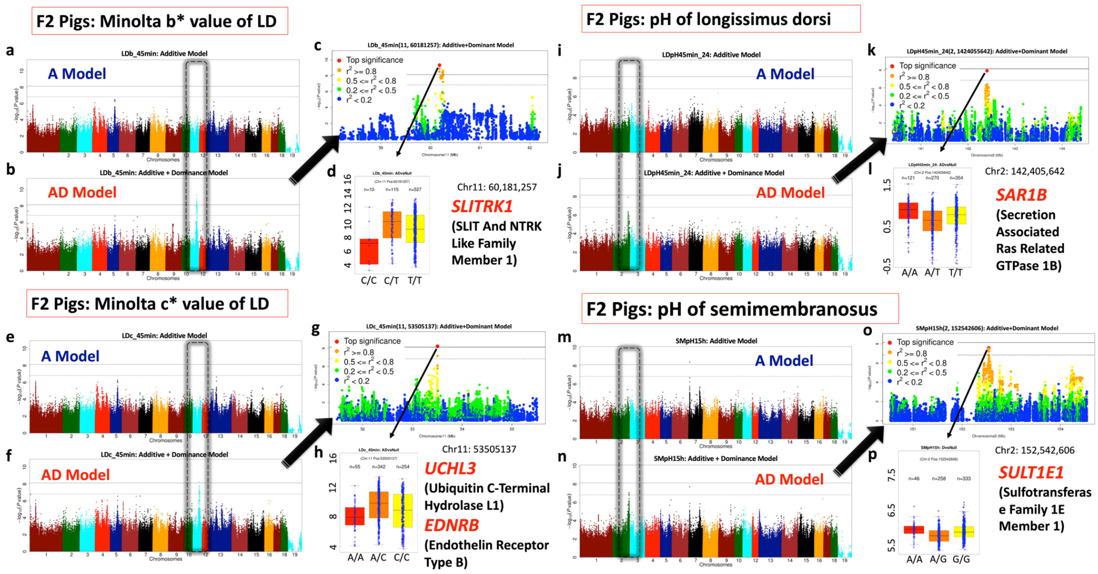

Fig. S3.1 Over-dominant QTLs in F2 pigs. (a-d) Manhattan plots of the Minolta b* value of longissimus dorsi muscle based on (a) A model and (b) AD model. (c) regional plot and (d) genotype boxplot showing phenotypic distribution of the peak SNP of AD model. Within each Manhattan plot, the solid and dashed horizontal line show suggestive and whole-genome significant thresholds and the black dashed box indicates the QTL which was only detected by AD model. Also shown are similarly structured plots for other three traits: (e-h) Minolta c* value of longissimus dorsi muscle, (i-l) the pH value of longissimus dorsi muscle, (m-p) pH value of semimembranosus (m-p).

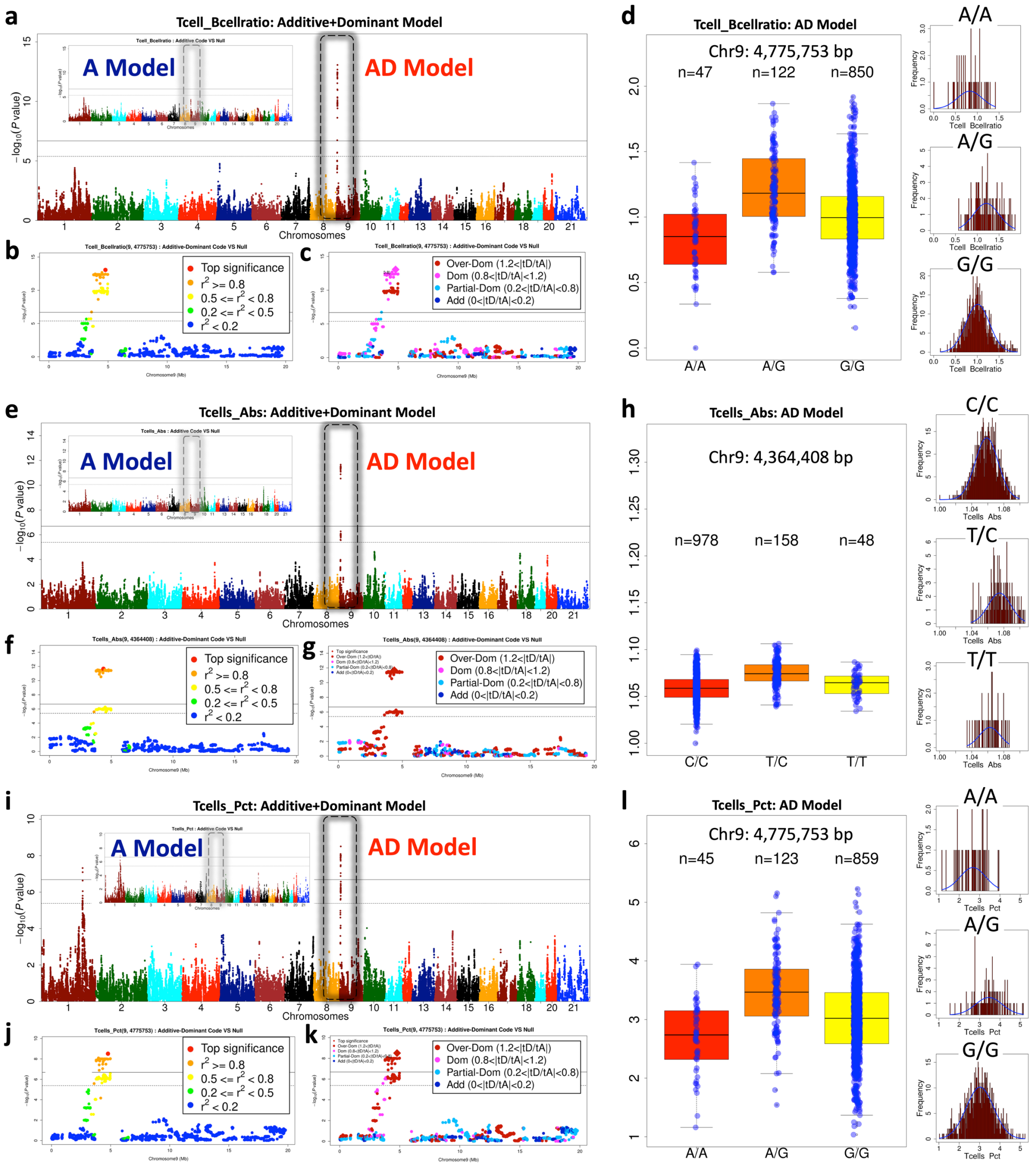

Fig. S3.2 Over-dominant HS rat QTLs. (a-d) Manhattan plots of the ratio of T cells to B cells based on A and AD models. Regional Manhattan plots of the peak SNP of AD model showing (b) the LD (r^2) with the peak SNP and (c) the different dominance types within the locus. Within each Manhattan plot, the solid and dashed horizontal lines represents suggestive and whole-genome significant thresholds, and the black dashed box highlights the QTL. (d) Boxplot and histogram of phenotypic distributions classified by the peak SNP genotypes. Similar plots shown in (e-h) for absolute T cells counts and (i-l) for proportion of T cells in WBC.

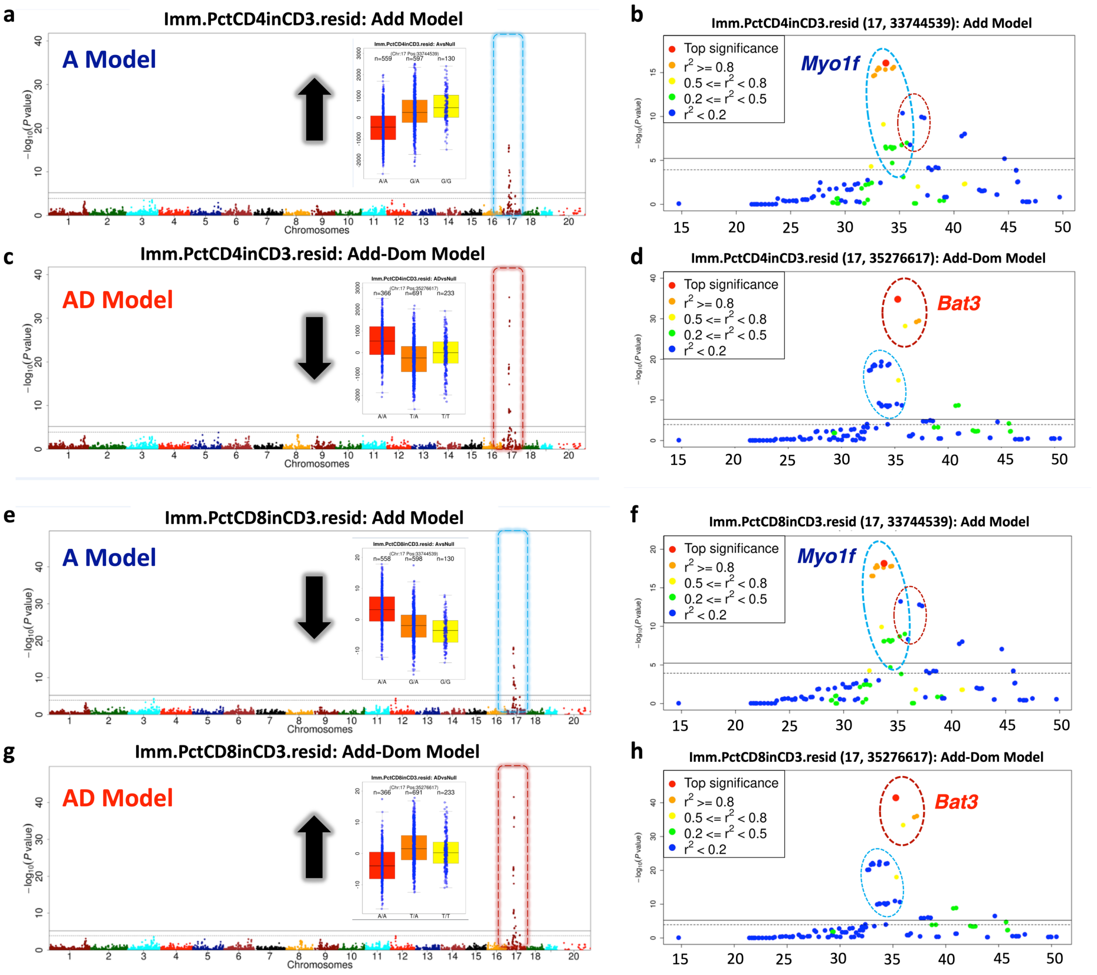

Fig. S3.3 Over-dominant QTLs implicate a novel causal gene regulating the proportions of CD4+ and CD8+ T cells in HS mice, compared to additive QTL modelling. (a-d) Manhattan plots and regional Manhattan plots of the proportion of CD4+ T cells in CD3+ T cells based on A model (a, b) and AD model (c, d), respectively. (e-h) Manhattan plots and regional Manhattan plots of the proportion of CD8+ T cells in CD3+ T cells based on A model (e, f) and AD model (g, h). Within each Manhattan plot, the blue and red dashed boxes highlight the QTLs detected by Add model and AD model separately, along with the boxplots of phenotypic distribution of the peak SNPs and black arrows are used to indicate the effect directions of peak SNPs. The blue and red dashed ellipses are used to highlight the linked SNP groups for each model in the regional plots, labelled by the closest annotated genes (*Bat3*: AD Model, *Myo1f*: A Model).

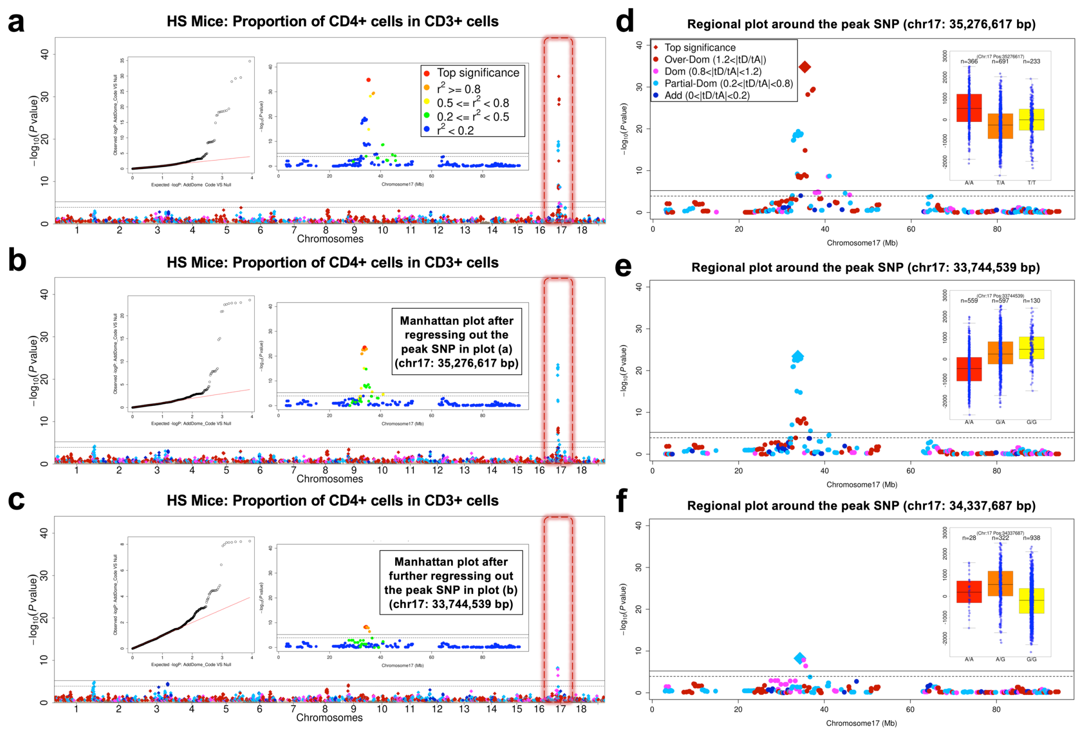

Fig. S3.4 Conditional GWAS of the proportion of CD4+ cells in CD3+ cells in HS mice. (a) Initial Manhattan plot after only regressing out regular covariates (gender, experimenter, month). (b) Manhattan plot after regressing out the peak SNP from (a) at chr17: 35,276,617 bp. (c) The Manhattan plot after additionally regressing out the peak SNP in (b) at chr17: 33,744,539 bp. Within each Manhattan plot (a-c), SNPs are colour coded by dominance classification, and insets show Q-Q plots and regional QTL plots around peak SNPs colour coded by R2 LD. (d-f) Three corresponding regional Manhattan plots and phenotype-genotype distributions around the peak SNPs of plots (a-c) in chromosome 17. SNPs coloured based on their dominance types.

Mean corpuscular volume

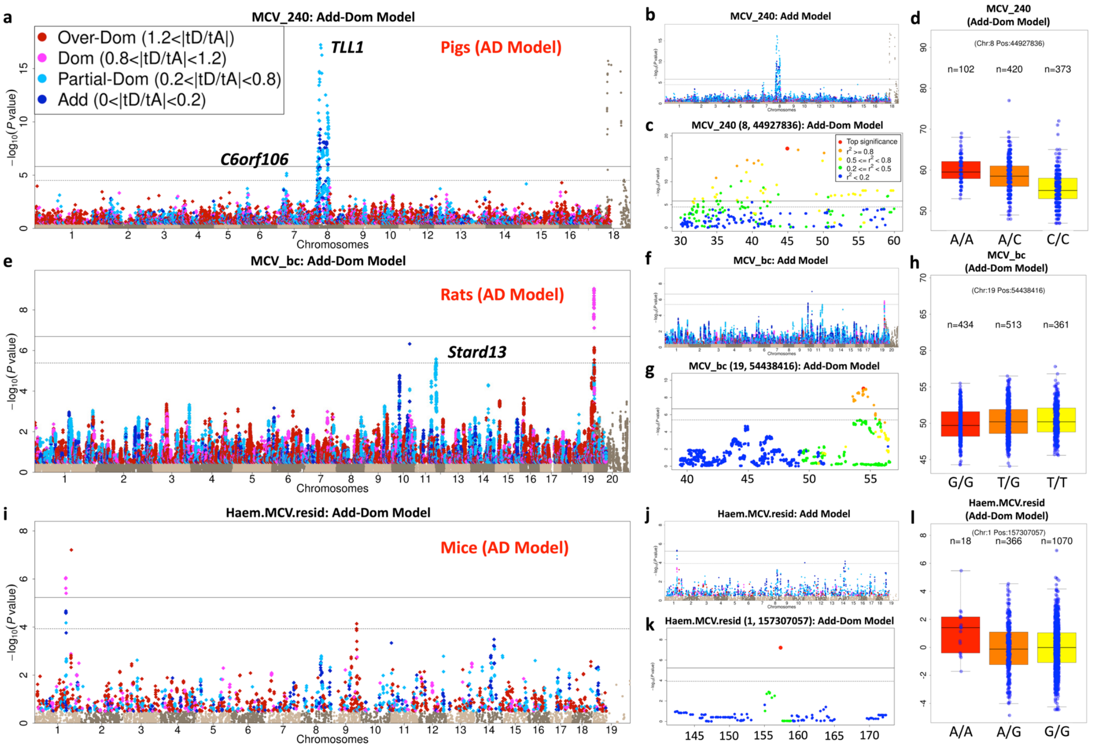

Fig. S4.1 Homologous QTLs for mean corpuscular volume (MCV). (a-d) Manhattan plots in F2 pigs based on (a) AD model and (b) A model (c) regional plot (d) phenotypic distribution boxplot of the peak SNP at chr8: 44,927,836 bp of AD model. (e-h) Homologous QTL for HS rats at the peak SNP chr19: 54,438,416 bp. (i-l) Homologous QTL in HS mice at peak SNP chr1: 157,307,057 bp. Within each Manhattan plot, the solid and dashed horizontal line represents the suggestive and whole-genome significance thresholds and all SNPs with -$\log_{10}(P)$ values > 0.5 are coloured according to their dominance classification.

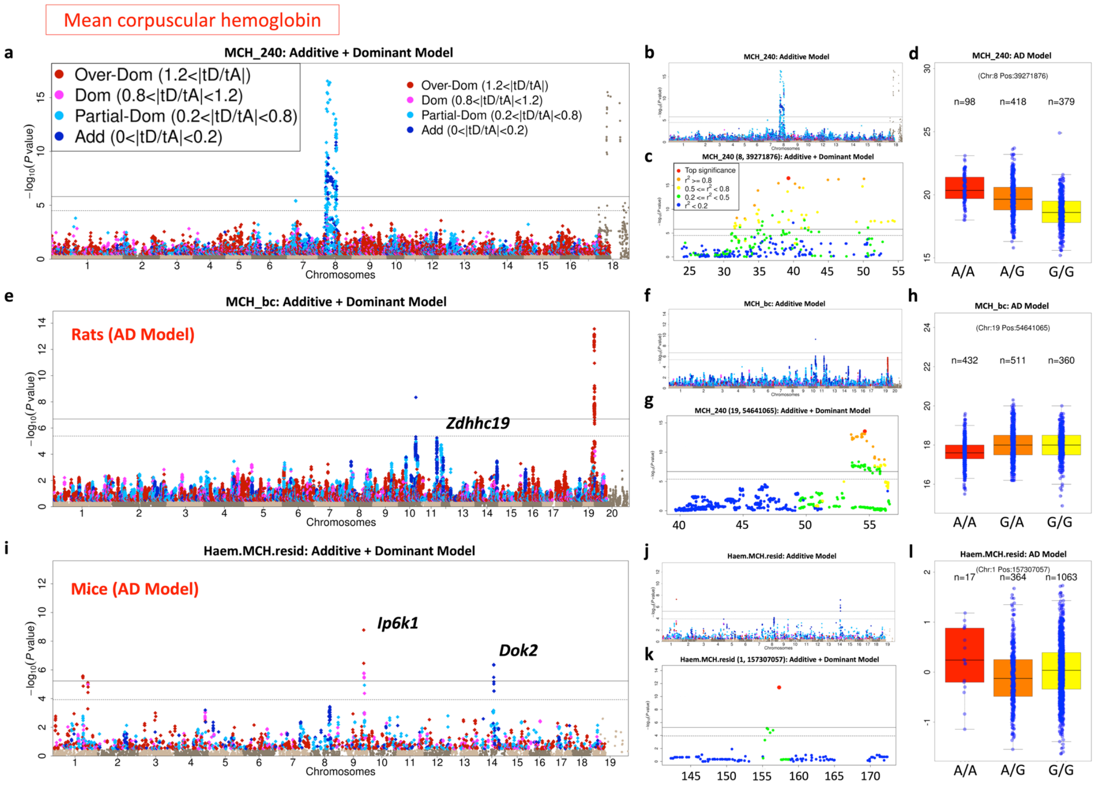

Fig. S4.2 Homologous QTLs for mean corpuscular volume (MCV). (a-d) Manhattan plots in F2 pigs based on (a) AD model and (b) A model (c) regional plot (d) phenotypic distribution boxplot of the peak SNP at chr8: 44,927,836 bp of AD model. (e-h) Homologous QTL for HS rats at the peak SNP chr19: 54,438,416 bp. (i-l) Homologous QTL in HS mice at peak SNP chr1: 157,307,057 bp. Within each Manhattan plot, the solid and dashed horizontal line represents the suggestive and whole-genome significance thresholds and all SNPs with -$\log_{10}(P)$ values > 0.5 are coloured according to their dominance classification.

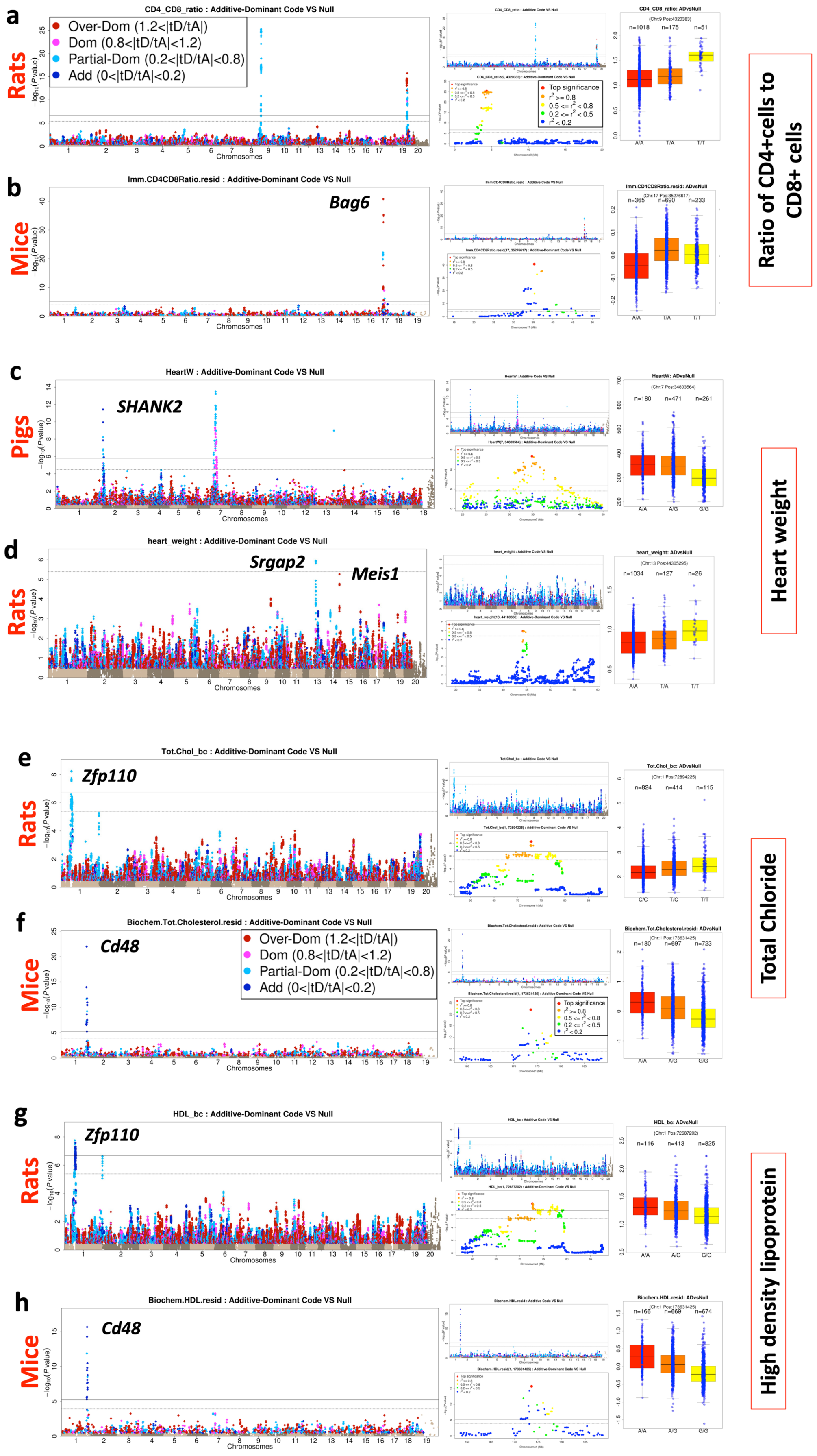
a
Rats
CD4_CD8_ratio : Additive-Dominant Code VS Null
Over-Dom (1.2<|tD/tA|)
Dom (0.8<|tD/tA|<1.2)
Partial-Dom (0.2<|tD/tA|<0.8)
Add (0<|tD/tA|<0.2)
Chromosomes
CD4_CD8_ratio: ADvsNull
n=1018
n=175
n=51
b
Mice
Imm.CD4CD8Ratio.resid : Additive-Dominant Code VS Null
Bag6
Chromosomes
Imm.CD4CD8Ratio.resid: ADvsNull
n=365
n=690
n=233
Ratio of CD4+cells to CD8+ cells
c
Pigs
HeartW : Additive-Dominant Code VS Null
SHANK2
Chromosomes
HeartW: ADvsNull
n=180
n=471
n=261
d
Rats
heart_weight : Additive-Dominant Code VS Null
Srgap2
Meis1
Chromosomes
heart_weight: ADvsNull
n=1034
n=127
n=26
Heart weight
e
Rats
Tot.Chol_bc : Additive-Dominant Code VS Null
Zfp110
Chromosomes
Tot.Chol_bc: ADvsNull
n=824
n=414
n=115
f
Mice
Biochem.Tot.Cholesterol.resid : Additive-Dominant Code VS Null
Cd48
Over-Dom (1.2<|tD/tA|)
Dom (0.8<|tD/tA|<1.2)
Partial-Dom (0.2<|tD/tA|<0.8)
Add (0<|tD/tA|<0.2)
Chromosomes
Biochem.Tot.Cholesterol.resid: ADvsNull
n=180
n=697
n=723
Total Chloride
g
Rats
HDL_bc : Additive-Dominant Code VS Null
Zfp110
Chromosomes
HDL_bc: ADvsNull
n=116
n=413
n=825
h
Mice
Biochem.HDL.resid : Additive-Dominant Code VS Null
Cd48
Chromosomes
Biochem.HDL.resid: ADvsNull
n=166
n=669
n=674
High density lipoprotein

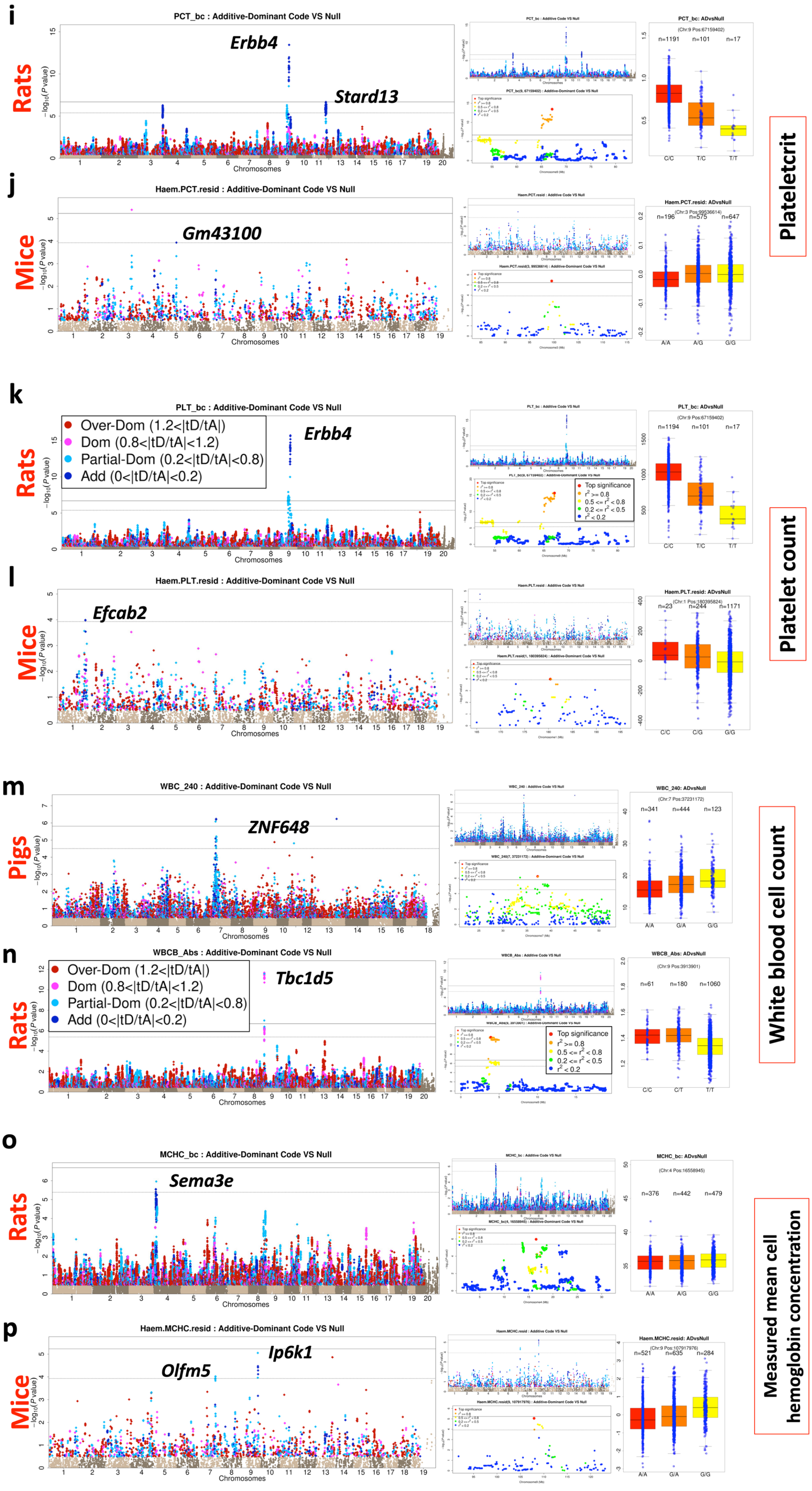
i
Rats
PCT_bc : Additive-Dominant Code VS Null
Erbb4
Stard13
PCT_bc: ADvsNull
n=1191
n=101
n=17
C/C
T/C
T/T
j
Mice
Haem.PCT.resid : Additive-Dominant Code VS Null
Gm43100
Haem.PCT.resid: ADvsNull
n=196
n=575
n=647
A/A
A/G
G/G
Plateletcrit
k
Rats
PLT_bc : Additive-Dominant Code VS Null
Over-Dom (1.2<|tD/tA|)
Dom (0.8<|tD/tA|<1.2)
Partial-Dom (0.2<|tD/tA|<0.8)
Add (0<|tD/tA|<0.2)
Erbb4
PLT_bc: ADvsNull
n=1194
n=101
n=17
C/C
T/C
T/T
l
Mice
Haem.PLT.resid : Additive-Dominant Code VS Null
Efcab2
Haem.PLT.resid: ADvsNull
n=23
n=244
n=1171
C/C
C/G
G/G
Platelet count
m
Pigs
WBC_240 : Additive-Dominant Code VS Null
ZNF648
WBC_240: ADvsNull
n=341
n=444
n=123
A/A
G/A
G/G
n
Rats
WBCB_Abs : Additive-Dominant Code VS Null
Over-Dom (1.2<|tD/tA|)
Dom (0.8<|tD/tA|<1.2)
Partial-Dom (0.2<|tD/tA|<0.8)
Add (0<|tD/tA|<0.2)
Tbc1d5
WBCB_Abs: ADvsNull
n=61
n=180
n=1060
C/C
C/T
T/T
White blood cell count
o
Rats
MCHC_bc : Additive-Dominant Code VS Null
Sema3e
MCHC_bc: ADvsNull
n=376
n=442
n=479
A/A
A/G
G/G
p
Mice
Haem.MCHC.resid : Additive-Dominant Code VS Null
Ip6k1
Olfm5
Haem.MCHC.resid: ADvsNull
n=521
n=635
n=284
A/A
G/A
G/G
Chromosomes
$-\log_{10}(P\,\text{value})$
Measured mean cell hemoglobin concentration

Fig. S4.3 Homologous QTLs. (a-b) Ratio of CD4+cells to CD8+ cells in rat and mouse. (c-d) Heart weight of pig and rat. (e-f) Total chloride in rat and mouse. (g-h) High density lipoprotein in rat and mouse. (i-j) Plateletcrit in rat and mouse. (k-l) Platelet count in rat and mouse. (m-n) White blood cell count in pig and rat. (o-p) mean cell hemoglobin concentration on rat and mouse. Within each panel are shown Manhattan plots based on AD model (left) and A model (middle upper) of the specified trait in the population, as well as the regional plot (middle lower) and phenotypic distribution boxplot (right) of the peak SNP in the AD model.

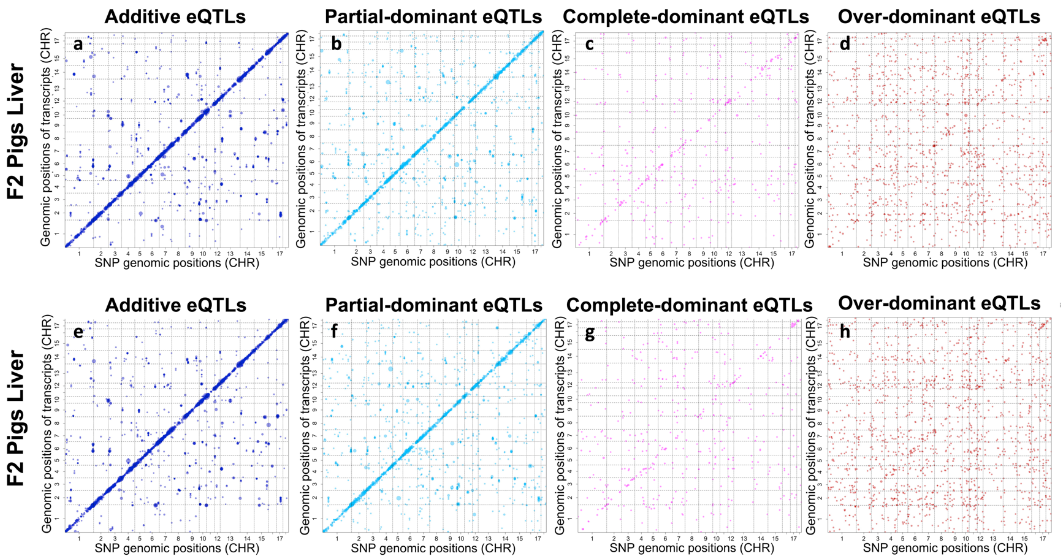

i

F2 Pigs	Liver		Muscle	
Suggestive Significant eQTLs	cis	trans	cis	trans
Add (A & PD)	1412	2678	1376	2735
Dom (CD & OD)	62	1813	51	2107
P-value of Fisher's Exact Test	3.7863e-187		2.9133e-219	
Whole-genome Significant eQTLs	cis	trans	cis	trans
Add (A & PD)	1216	1476	1153	1395
Dom (CD & OD)	37	153	23	140
P-value of Fisher's Exact Test	7.4627e-13		1.8378e-16	

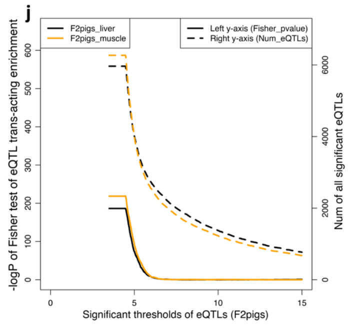

Fig. S5.1 Trans-acting enrichment among dominant eQTLs in liver and muscle in F2 pigs. (a-h) eQTL locations of transcript level eQTLs, filtered by dominance type. Each dot represents an eQTL significant at suggestive level (i.e. one false positive expected per transcript). x-axis: eQTL chromosome positions (bp), y-axis: physical gene location. First row (a-d): liver; Second row (e-h): muscle. The four columns represent dominance types (blue: additive A, sky blue: partial-dominant PD, purple: complete-dominant CD, red: over-dominant OD). (i) Fisher's exact test for the trans-acting enrichment among dominance eQTLs based on the suggestive significant threshold and the whole-genome significant threshold respectively. (j) Dominance trans-acting enrichment (left y-axis, solid lines) and the counts of significant eQTLs (right y-axis, dashed lines) under different -log10(P) eQTL significance thresholds (x-axis). Enrichment is quantified by the -log10(P) values of Fisher exact test between dominance (Add/Dom eQTLs) and regulation types (cis/trans-acting) of significant eQTLs within rat liver (black) and muscle (orange), respectively.

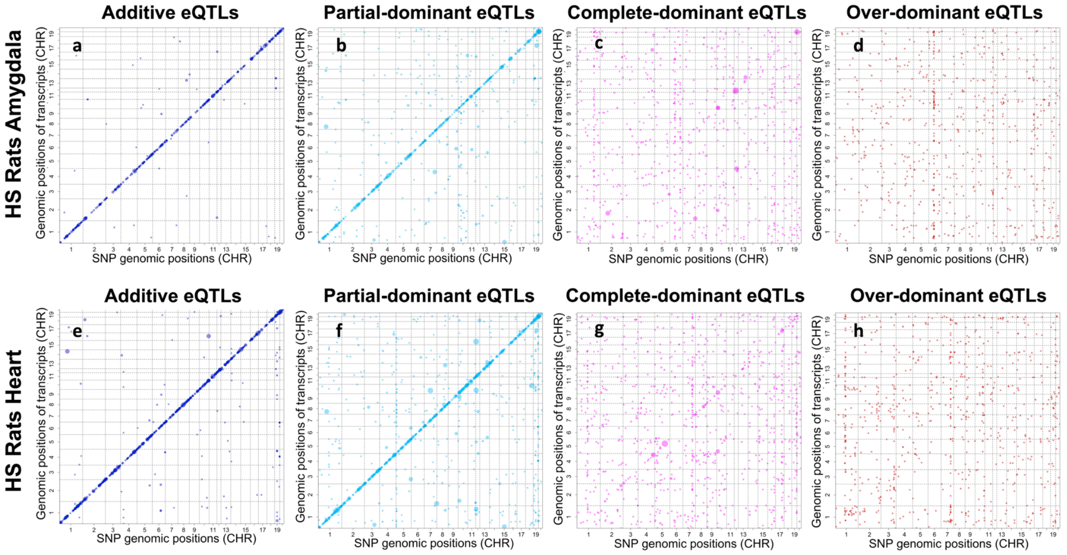

i

HS Rats (Gene)	Amygdala		Heart	
Suggestive Significant eQTLs	cis	trans	cis	trans
Add (A & PD)	782	410	1389	737
Dom (CD & OD)	27	1488	46	1766
P-value of Fisher's Exact Test	< 2.2e-324		< 2.2e-324	
Whole-genome Significant eQTLs	cis	trans	cis	trans
Add (A & PD)	572	134	1069	216
Dom (CD & OD)	15	260	26	302
P-value of Fisher's Exact Test	2.8913e-114		3.2124e-149	

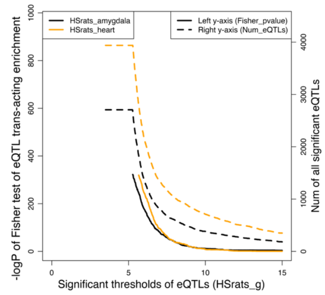

Fig. S5.2 Trans-acting enrichment among dominant eQTLs of amygdala and heart tissues in HS rats. (a-h) eQTL locations of gene level eQTLs, filtered by dominance type. Each dot represents an eQTL significant at suggestive level (i.e. one false positive expected per transcript). x-axis: eQTL chromosome positions (bp), y-axis: physical gene location. First row (a-d): amygdala; Second row (e-h): heart. The four columns represent dominance types (blue: additive A, sky blue: partial-dominant PD, purple: complete-dominant CD, red: over-dominant OD). (i) Fisher's exact test for the trans-acting enrichment among dominance eQTLs based on the suggestive significant threshold and the whole-genome significant threshold respectively. (j) Dominance trans-acting enrichment (left y-axis, solid lines) and the counts of significant eQTLs (right y-axis, dashed lines) under different -log10(P) eQTL significance thresholds (x-axis). Enrichment is quantified by the -log10(P) values of Fisher exact test between dominance (Add/Dom eQTLs) and regulation types (cis/trans-acting) of significant eQTLs within rat amygdala (black) and heart (orange), respectively.

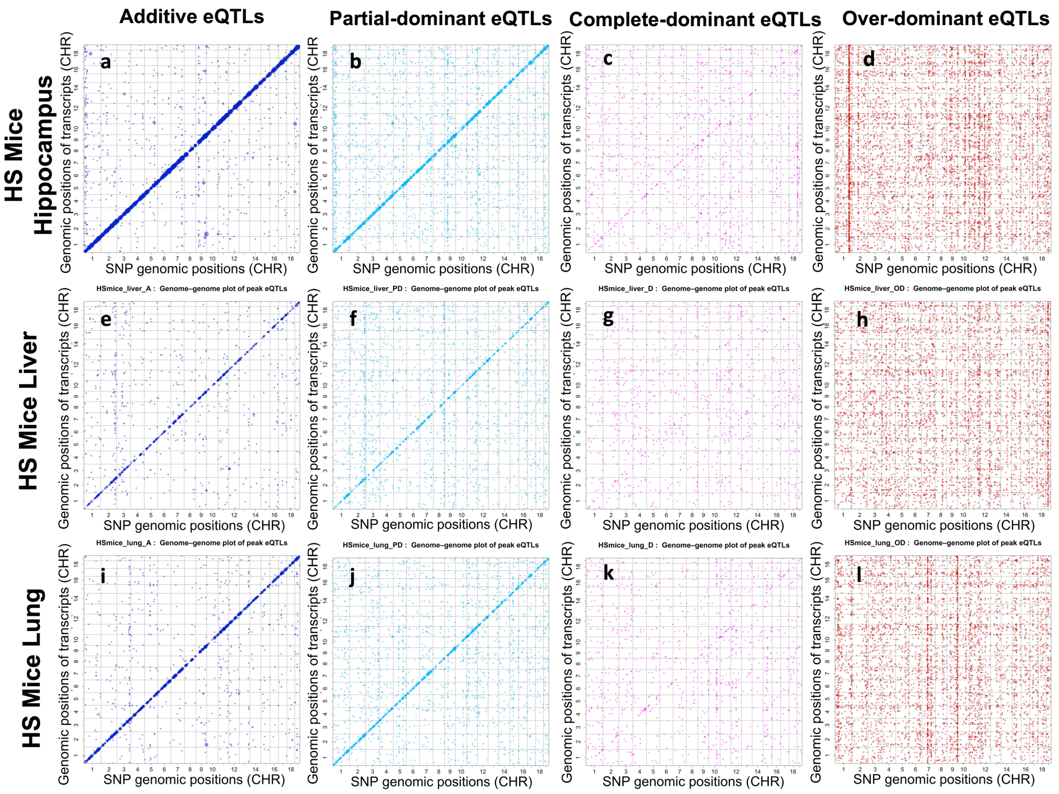

m

HS Rats (Transcript)	Hippocampus		Liver		Lung	
Suggestive Significant eQTLs	cis	trans	cis	trans	cis	trans
Add (A & PD)	2884	3261	839	2604	1496	2648
Dom (CD & OD)	113	8275	51	6592	78	6826
P-value of Fisher's Exact Test	< 2.2e-324		< 2.2e-324		< 2.2e-324	
Whole-genome Significant eQTLs	cis	trans	cis	trans	cis	trans
Add (A & PD)	2414	478	614	191	1167	275
Dom (CD & OD)	37	444	17	315	27	340
P-value of Fisher's Exact Test	2.8681e-241		3.5402e-120		2.8614e-158	

n

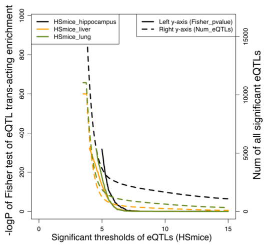

Fig. S5.3 Trans-acting enrichment among dominant eQTLs of hippocampus, liver and lung tissues in HS mice. (a-h) eQTL locations of transcript level eQTLs, filtered by dominance type. Each dot represents an eQTL significant at suggestive level (i.e. one false positive expected per transcript). x-axis: eQTL chromosome positions (bp), y-axis: physical gene location. First row (a-d): hippocampus; Second row (e-h): liver; Third row (i-l): lung. The four columns represent dominance types (blue: additive A, sky blue: partial-dominant PD, purple: complete-dominant CD, red: over-dominant OD). (m) Fisher's exact test for the trans-acting enrichment among dominance eQTLs based on the suggestive significant threshold and the whole-genome significant threshold respectively. (n) Dominance trans-acting enrichment (left y-axis, solid lines) and the counts of significant eQTLs (right y-axis, dashed lines) under different -log10(P) eQTL significance thresholds (x-axis). Enrichment is quantified by the -log10(P) values of Fisher exact test between dominance (Add/Dom eQTLs) and regulation types (cis/trans-acting) of significant eQTLs within mouse hippocampus (black), liver (orange) and heart (green), respectively.

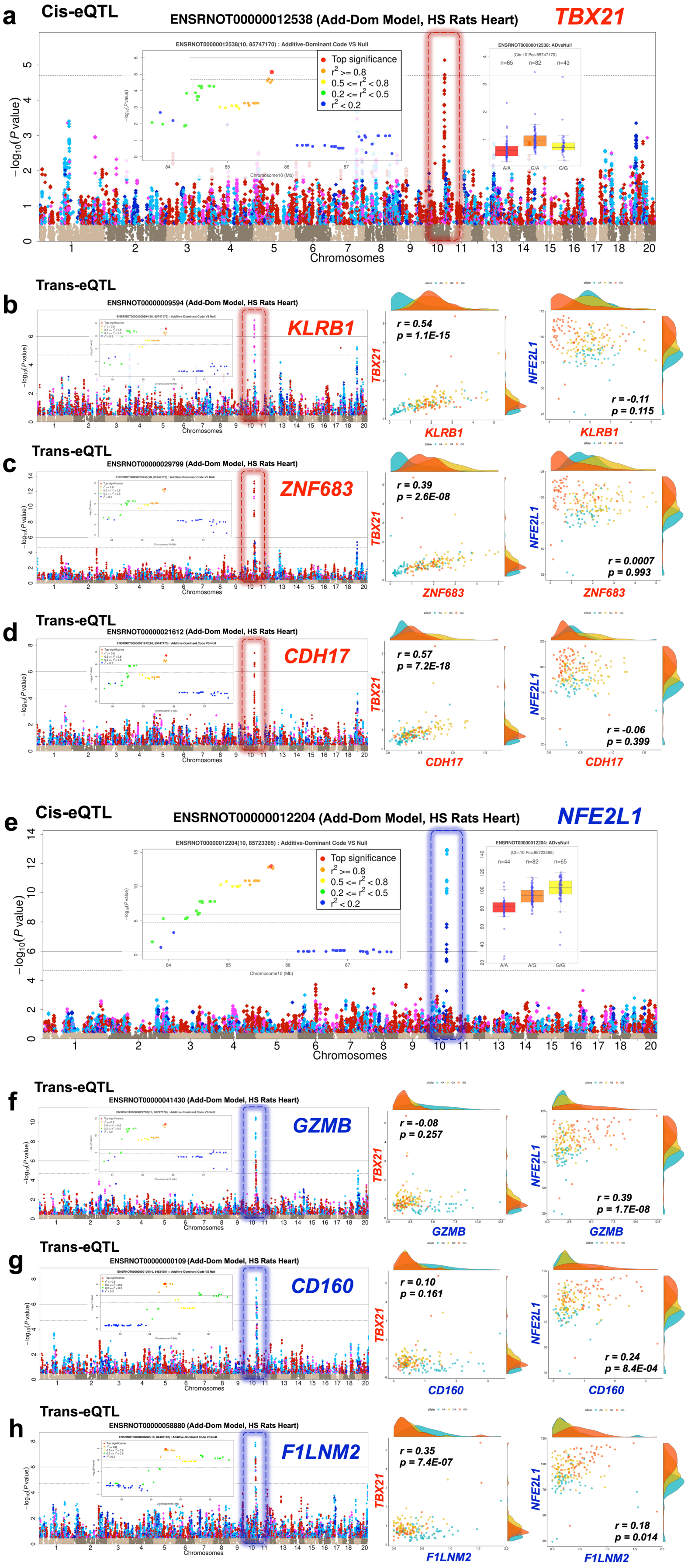
a
Cis-eQTL
ENSRNOT00000012538 (Add-Dom Model, HS Rats Heart)
TBX21
Chromosomes
b
Trans-eQTL
ENSRNOT00000009594 (Add-Dom Model, HS Rats Heart)
KLRB1
r = 0.54
p = 1.1E-15
r = -0.11
p = 0.115
c
Trans-eQTL
ENSRNOT00000029799 (Add-Dom Model, HS Rats Heart)
ZNF683
r = 0.39
p = 2.6E-08
r = 0.0007
p = 0.993
d
Trans-eQTL
ENSRNOT00000021612 (Add-Dom Model, HS Rats Heart)
CDH17
r = 0.57
p = 7.2E-18
r = -0.06
p = 0.399
e
Cis-eQTL
ENSRNOT00000012204 (Add-Dom Model, HS Rats Heart)
NFE2L1
Chromosomes
f
Trans-eQTL
ENSRNOT00000041430 (Add-Dom Model, HS Rats Heart)
GZMB
r = -0.08
p = 0.257
r = 0.39
p = 1.7E-08
g
Trans-eQTL
ENSRNOT00000000109 (Add-Dom Model, HS Rats Heart)
CD160
r = 0.10
p = 0.161
r = 0.24
p = 8.4E-04
h
Trans-eQTL
ENSRNOT00000058880 (Add-Dom Model, HS Rats Heart)
F1LNM2
r = 0.35
p = 7.4E-07
r = 0.18
p = 0.014

Fig. S6.1 Dominant cis and trans transcript eQTLs at the hotspot chr10:85Mb-86Mb in HS rat heart. (a, e) cis-eQTLs with different inheritance modes, (a) *Tbx21* (over-dominant) and (e) *Nf32l1* (partial-dominant). (b-d) Manhattan plots of over-dominant trans-eQTLs for genes *Klrb1*, *Znf683*, *Cdh17*. (f-h) Manhattan plots of partial-dominant trans-eQTLs for genes *Gzmb*, *Cd160*, *F1lnm2*. Within each Manhattan plot, the eQTL is marked by a dotted rectangular frame, with the same colour as the peak SNP dot (Blue - additive; Sky blue - partial-dominant; Purple - complete-dominant; Red - over-dominant), and all linked SNPs with -log10(P) > 0.5 are coloured the same. The regional Manhattan plots of the peak signal of each eQTL and the scatter plots of two cis-eQTLs are also shown as insets. The pairs of scatter plots to the right of each Manhattan plot compare the expression of each trans-eQTL with *Tbx21, Nf32l1,* together with their Pearson correlation coefficients and the p-value of test that the correlation is zero. Each dot represents one animal, colour-coded by the genotype of the peak SNP.

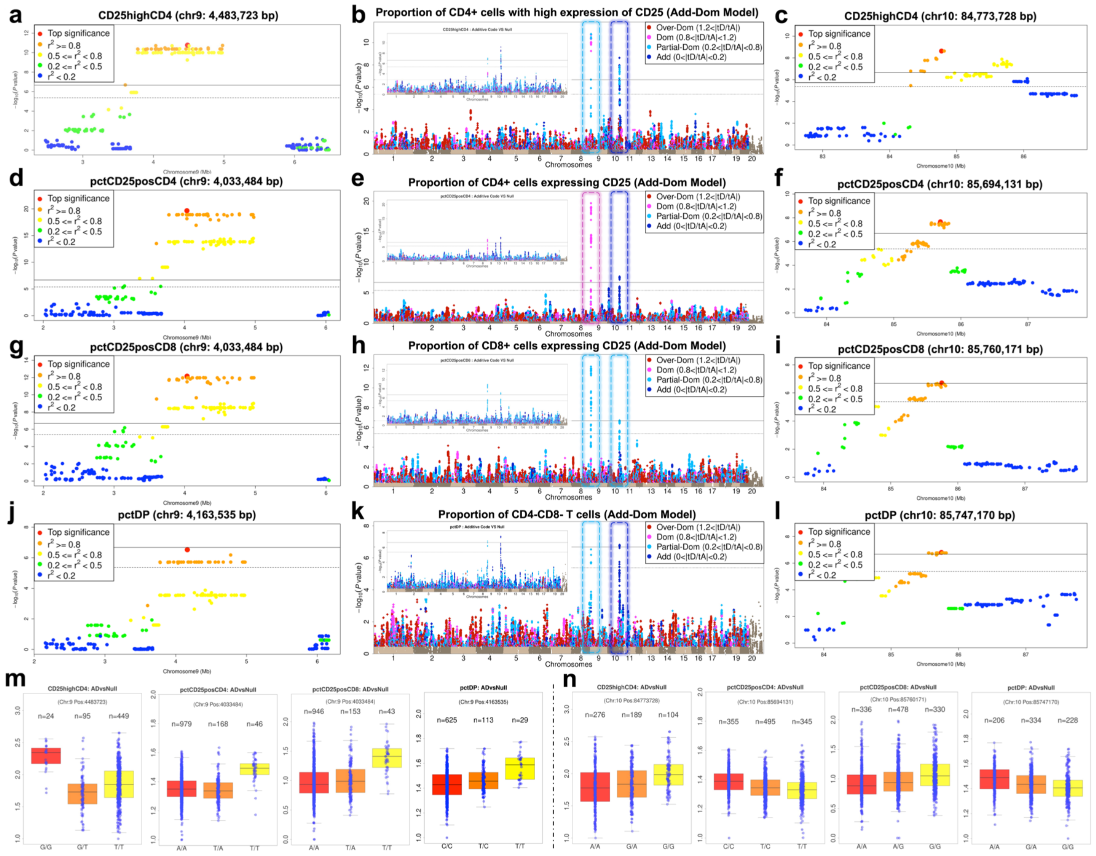

Fig. S6.2 Interplay of immunology-related QTLs in HS rats. A locus at chr10: 85Mb-86Mb (cf Fig. 5) is an additive QTL for four rat immunology traits, which are also associated with dominant QTLs at chr9: 4Mb-5Mb**.** Manhattan plots based on AD model for (i) the proportions of CD4+ cells with high expression of CD25 (a-c) (ii) the proportions of CD4+ cells expressing CD25 (d-f) (iii) the proportions of CD8+ cells expressing CD25 (g-i) (iv) the proportions of CD4+CD8- cells (j-l), as well as two corresponding regional Manhattan plots (the left column, the dominant chr9 QTL; the right column, the additive chr10 QTL). Within each Manhattan plot (b, e, h, k), the corresponding GWAS based on A model are shown in the left upper corner. The corresponding phenotype-genotype distribution plots at peak SNPs are also included (peak SNPs located at chromosome 9, m; peak SNPs located at chromosome 10, n).

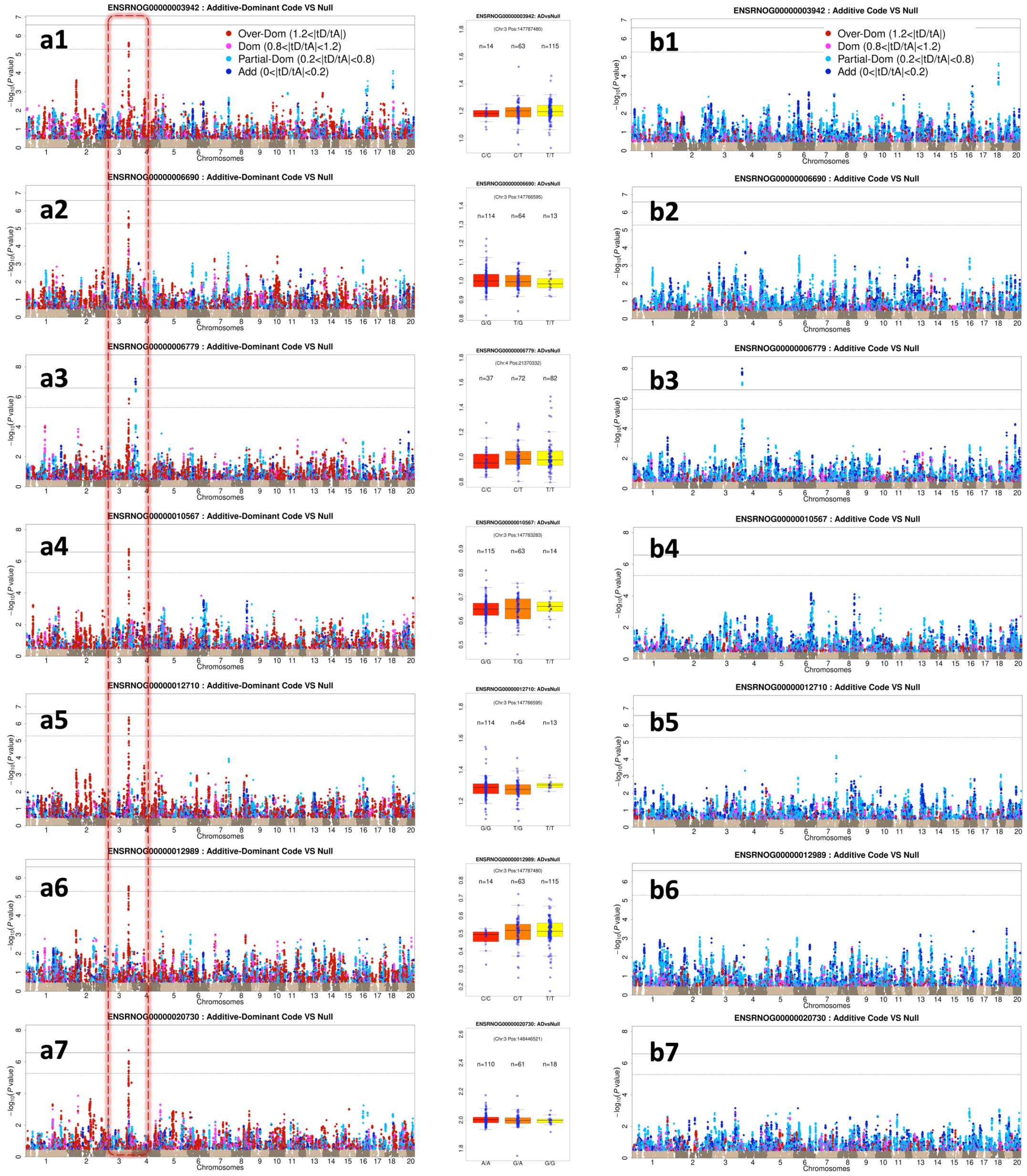
ENSRNOG00000003942 : Additive-Dominant Code VS Null
ENSRNOG00000003942 : Additive Code VS Null
a1
b1
Over-Dom (1.2<|tD/tA|)
Dom (0.8<|tD/tA|<1.2)
Partial-Dom (0.2<|tD/tA|<0.8)
Add (0<|tD/tA|<0.2)
ENSRNOG00000006690 : Additive-Dominant Code VS Null
ENSRNOG00000006690 : Additive Code VS Null
a2
b2
ENSRNOG00000006779 : Additive-Dominant Code VS Null
ENSRNOG00000006779 : Additive Code VS Null
a3
b3
ENSRNOG00000010567 : Additive-Dominant Code VS Null
ENSRNOG00000010567 : Additive Code VS Null
a4
b4
ENSRNOG00000012710 : Additive-Dominant Code VS Null
ENSRNOG00000012710 : Additive Code VS Null
a5
b5
ENSRNOG00000012989 : Additive-Dominant Code VS Null
ENSRNOG00000012989 : Additive Code VS Null
a6
b6
ENSRNOG00000020730 : Additive-Dominant Code VS Null
ENSRNOG00000020730 : Additive Code VS Null
a7
b7
Chromosomes

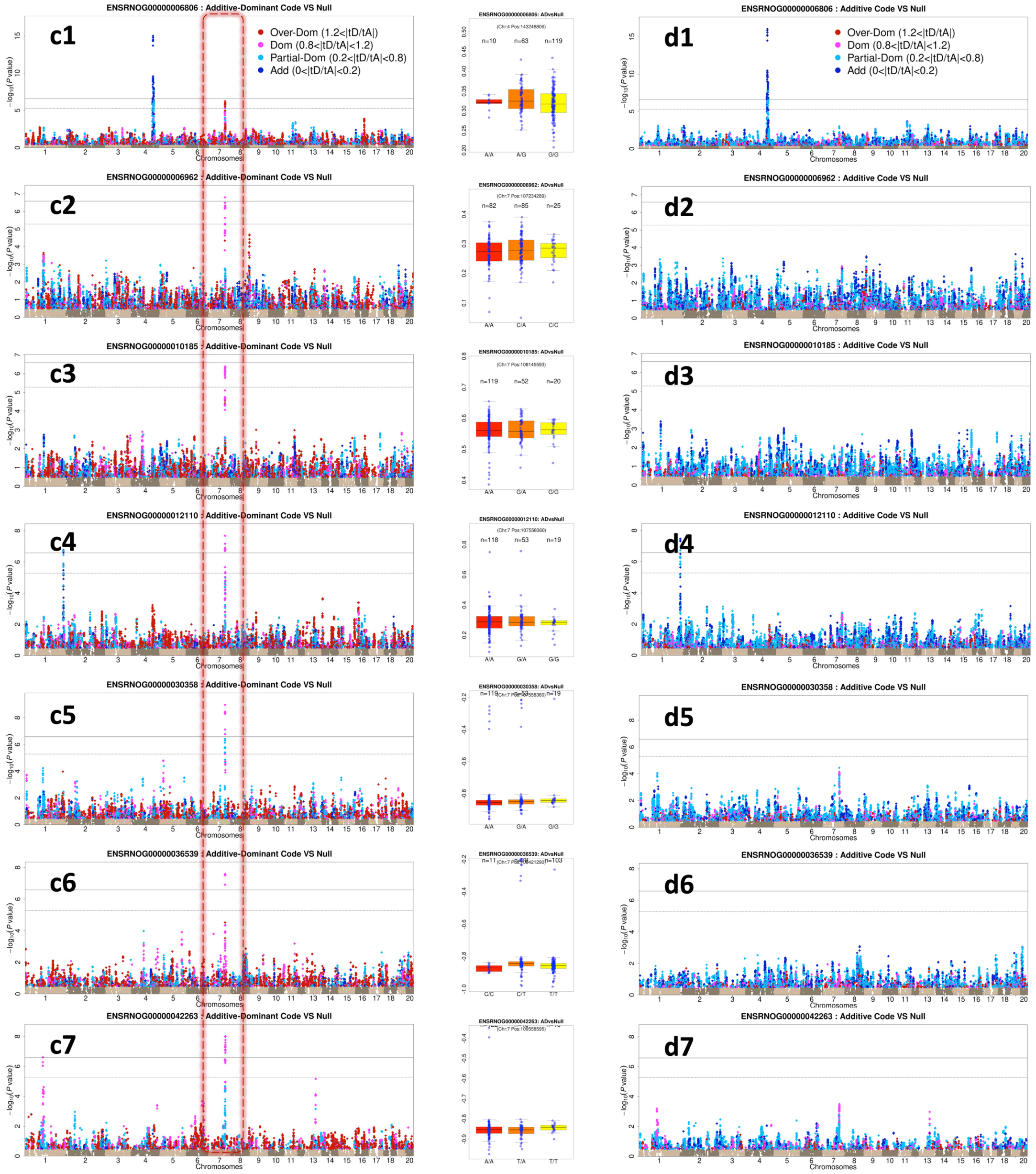

Fig. S6.3 Dominant eQTLs detected by AD model from two trans-acting hotspots (a1-a7, chr3: 147Mb-149Mb; c1-c7, chr7: 107Mb-108Mb) in HS rat heart. Left two columns containing the Manhattan plots based on AD model (a1-a7) and A model (b1-b7), (from the top down the genes *Asnsd1*, *Tmem168*, *Crot*, *Angel1*, *Ubac2*, *Serinc2* and *Nbr1*) that are trans-regulated by over-dominant eQTLs. Right two columns show Manhattan plots based on AD model (c1-c7) and A model (d1-d7), (from top down *Setmar*, *Stk32c*, *RGD1306941*, *Col17a1*, *Olr312*, *U6* and *D4A0M7*_RAT). Red dashed boxes indicate the hotspots.

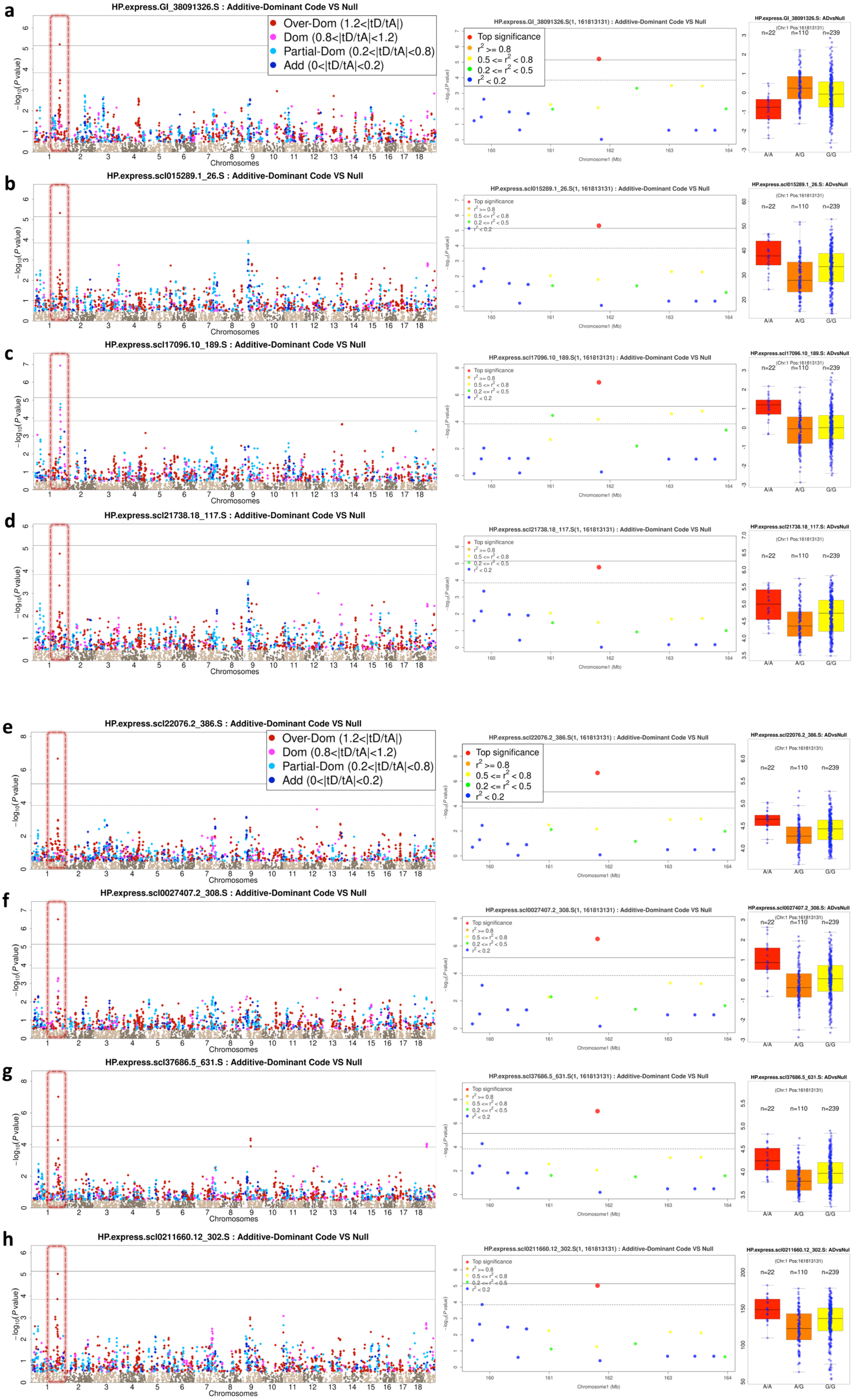
a
HP.express.GI_38091326.S : Additive-Dominant Code VS Null
Over-Dom (1.2<|tD/tA|)
Dom (0.8<|tD/tA|<1.2)
Partial-Dom (0.2<|tD/tA|<0.8)
Add (0<|tD/tA|<0.2)
Chromosomes
HP.express.GI_38091326.S(1, 161813131) : Additive-Dominant Code VS Null
Top significance
Chromosome1 (Mb)
b
HP.express.scl015289.1_26.S : Additive-Dominant Code VS Null
HP.express.scl015289.1_26.S(1, 161813131) : Additive-Dominant Code VS Null
c
HP.express.scl17096.10_189.S : Additive-Dominant Code VS Null
HP.express.scl17096.10_189.S(1, 161813131) : Additive-Dominant Code VS Null
d
HP.express.scl21738.18_117.S : Additive-Dominant Code VS Null
HP.express.scl21738.18_117.S(1, 161813131) : Additive-Dominant Code VS Null
e
HP.express.scl22076.2_386.S : Additive-Dominant Code VS Null
HP.express.scl22076.2_386.S(1, 161813131) : Additive-Dominant Code VS Null
f
HP.express.scl0027407.2_308.S : Additive-Dominant Code VS Null
HP.express.scl0027407.2_308.S(1, 161813131) : Additive-Dominant Code VS Null
g
HP.express.scl37686.5_631.S : Additive-Dominant Code VS Null
HP.express.scl37686.5_631.S(1, 161813131) : Additive-Dominant Code VS Null
h
HP.express.scl0211660.12_302.S : Additive-Dominant Code VS Null
HP.express.scl0211660.12_302.S(1, 161813131) : Additive-Dominant Code VS Null
n=22
n=110
n=239
A/A
A/G
G/G

Fig. S6.4 Dominant eQTLs from a trans-acting hotspot in HS mice hippocampus. Manhattan plots, regional plots and phenotypic distribution boxplots of the peak SNPs from GWAS across eight genes in HS mice hippocampus. Each of these genes is simultaneously trans-regulated by a dominant cis eQTL located in the hotspot (chr1: 161Mb-163Mb), highlighted by red dashed boxes.

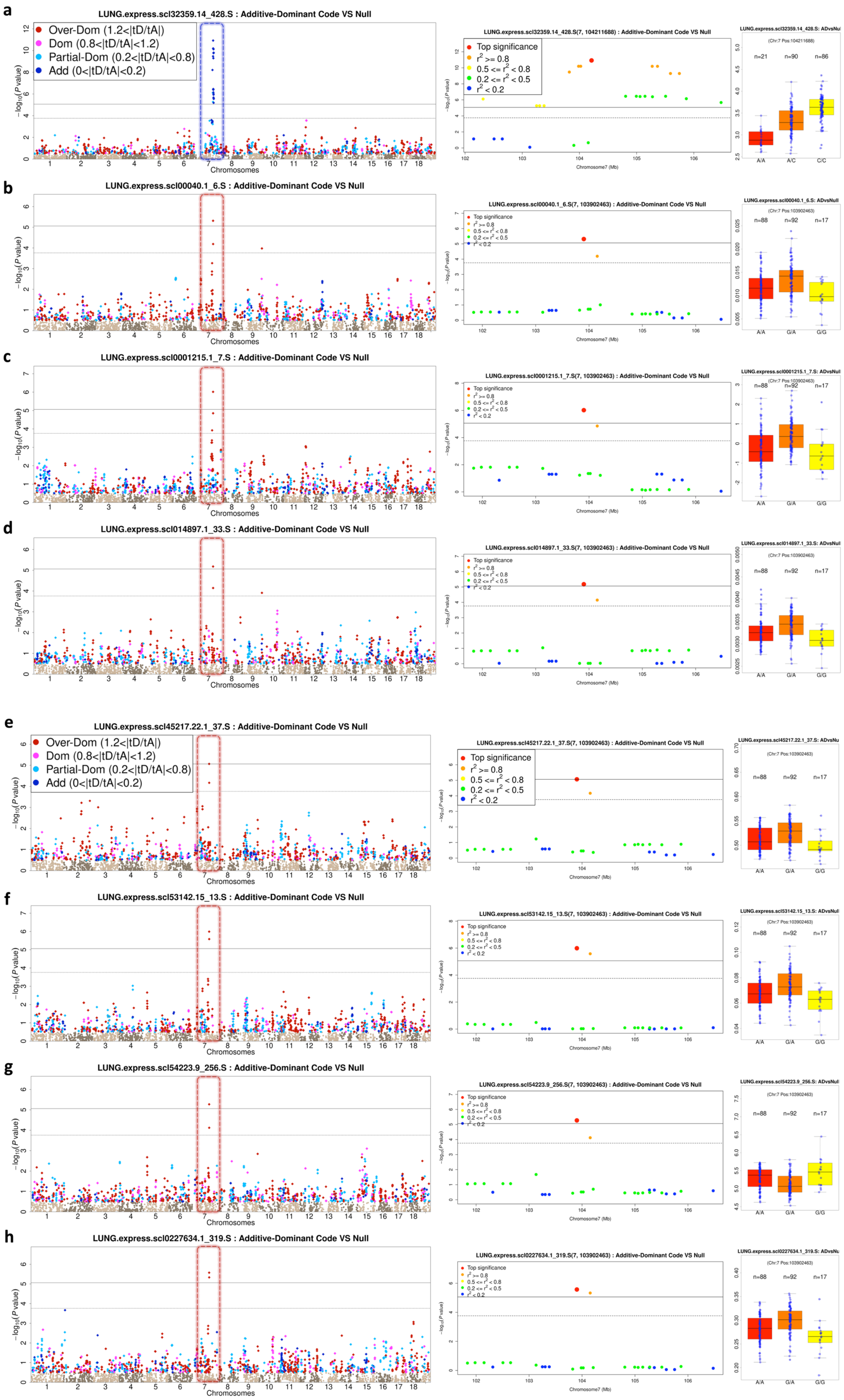
a
LUNG.express.scl32359.14_428.S : Additive-Dominant Code VS Null
Over-Dom (1.2<|tD/tA|)
Dom (0.8<|tD/tA|<1.2)
Partial-Dom (0.2<|tD/tA|<0.8)
Add (0<|tD/tA|<0.2)
−log10(P value)
Chromosomes
LUNG.express.scl32359.14_428.S(7, 104211688) : Additive-Dominant Code VS Null
Top significance
r2 >= 0.8
0.5 <= r2 < 0.8
0.2 <= r2 < 0.5
r2 < 0.2
Chromosome7 (Mb)
n=21
n=90
n=86
A/A
A/C
C/C
b
LUNG.express.scl00040.1_6.S : Additive-Dominant Code VS Null
LUNG.express.scl00040.1_6.S(7, 103902463) : Additive-Dominant Code VS Null
n=88
n=92
n=17
A/A
G/A
G/G
c
LUNG.express.scl0001215.1_7.S : Additive-Dominant Code VS Null
LUNG.express.scl0001215.1_7.S(7, 103902463) : Additive-Dominant Code VS Null
d
LUNG.express.scl014897.1_33.S : Additive-Dominant Code VS Null
LUNG.express.scl014897.1_33.S(7, 103902463) : Additive-Dominant Code VS Null
e
LUNG.express.scl45217.22.1_37.S : Additive-Dominant Code VS Null
LUNG.express.scl45217.22.1_37.S(7, 103902463) : Additive-Dominant Code VS Null
f
LUNG.express.scl53142.15_13.S : Additive-Dominant Code VS Null
LUNG.express.scl53142.15_13.S(7, 103902463) : Additive-Dominant Code VS Null
g
LUNG.express.scl54223.9_256.S : Additive-Dominant Code VS Null
LUNG.express.scl54223.9_256.S(7, 103902463) : Additive-Dominant Code VS Null
h
LUNG.express.scl0227634.1_319.S : Additive-Dominant Code VS Null
LUNG.express.scl0227634.1_319.S(7, 103902463) : Additive-Dominant Code VS Null

Fig. S6-5 Dominant eQTLs from a trans-acting hotspot in HS mice lung. Manhattan plots, regional plots and phenotypic distribution boxplots at the peak SNPs across eight genes in HS mice lung, showing seven genes *Mef2a* (b), *Rassf4* (c), *Trip12* (d), *Dis3* (e), *Entpd1* (f), *Rbmx* (g), *Camsap1* (h) that are simultaneously trans-regulated by an over-dominant cis eQTL for *Nars2* at chr7:103Mb-106Mb (a). Blue and red dashed boxes to highlight the hotspot.

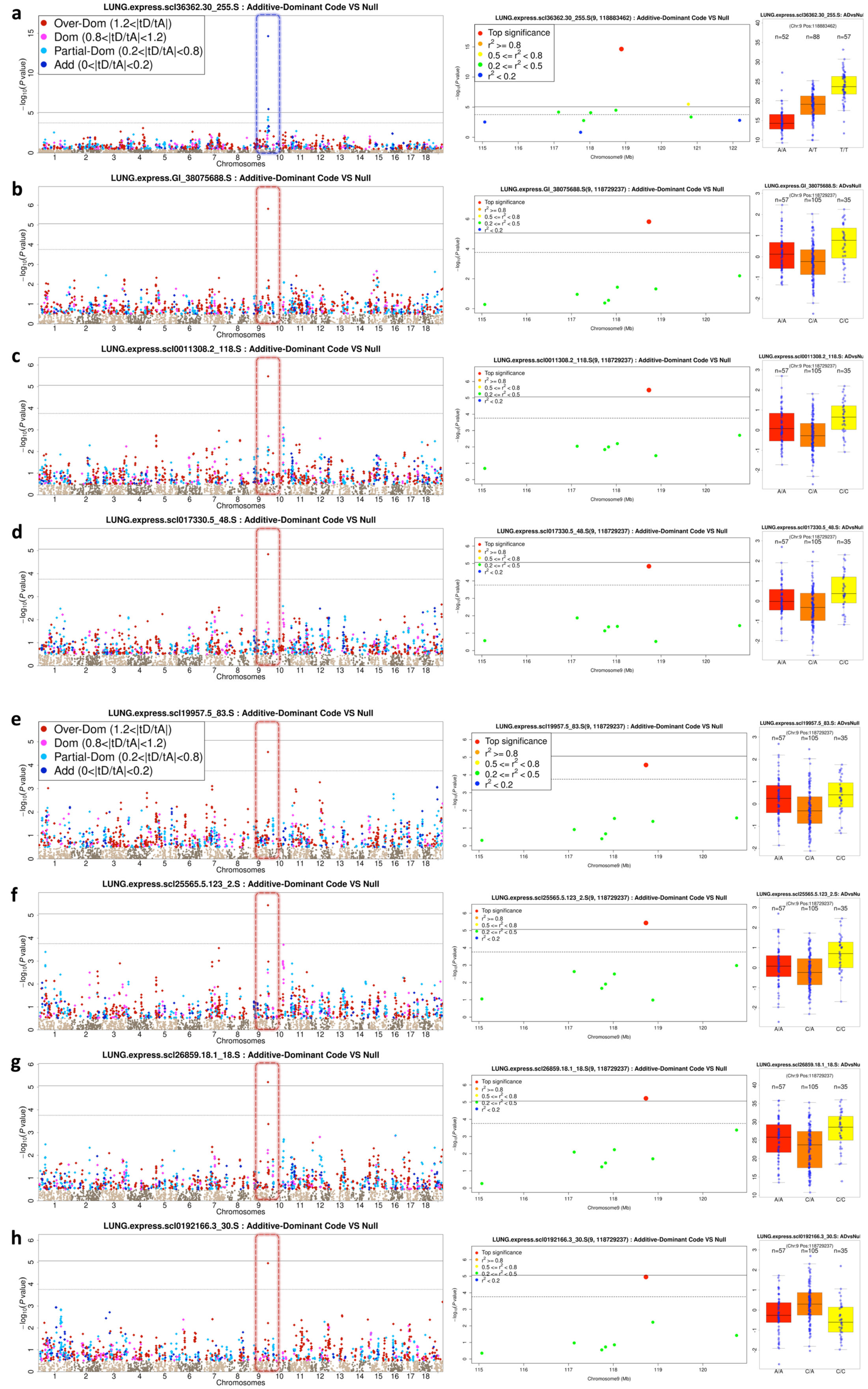

Fig. S6.6 Dominant eQTLs from a trans-acting hotspot in HS mice lung. Manhattan

plots, regional plots and phenotypic distribution boxplots at the peak SNPs across eight genes in HS mice lung, showing seven genes *AL845491.3* (b), *Abi1* (c), *Minpp1* (d), *Ttpal* (e), *Akirin2* (f), *Ptpn12* (g), *Sardh* (h) that are simultaneously trans-regulated by an over-dominant cis eQTL for *Itga9* at chr9:117Mb-121Mb (a). Blue and red dashed boxes highlight the hotspot.

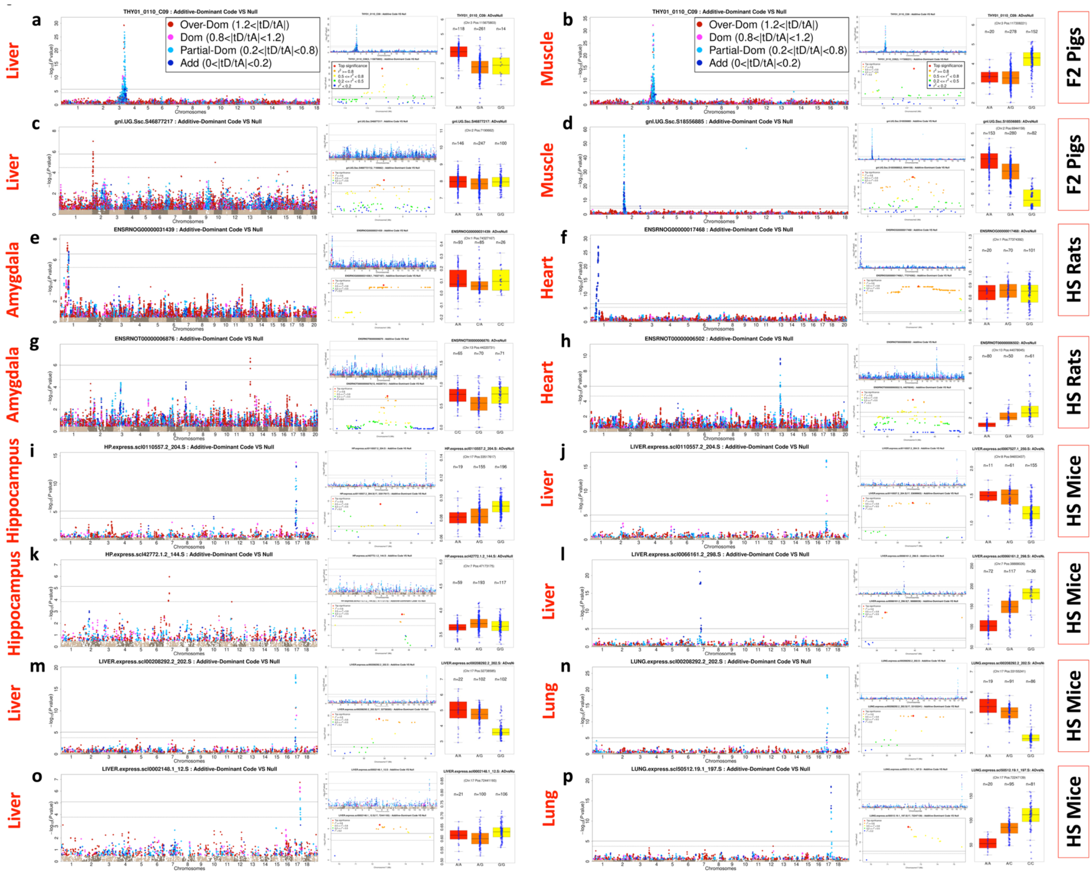

Fig. S6.7 Tissue-conserved and tissue-specific dominant eQTLs. Examples of tissue-conserved eQTLs of *GPN1* between liver (a) and muscle (b) in F2 pigs; *H2-Q1* between hippocampus (i) and liver (j) in HS mice; *9030612M13Rik* between liver (m) and lung (n) in HS mice. Examples of tissue-specific eQTLs shared between *Mgea5* of liver (c) and *Nudt22* of muscle (d) in F2 pigs; *ENSRNOG00000031439* of amygdala (e) and *Trappc6a* of heart (f) in HS rats; *Nut* of amygdala (g) and *Dyrk3* of heart (h) in HS rats; *Dio3* of hippocampus (h) and *Pop4* of liver (l) in HS mice; *St8sia5* of liver (o) and *Clip4* of lung (p) in HS mice. Within each panel are shown the Manhattan plots based on AD model (left) and A model (middle upper) as well as the regional plot (middle lower) and phenotypic distribution boxplot (right) of the peak SNP of AD model.

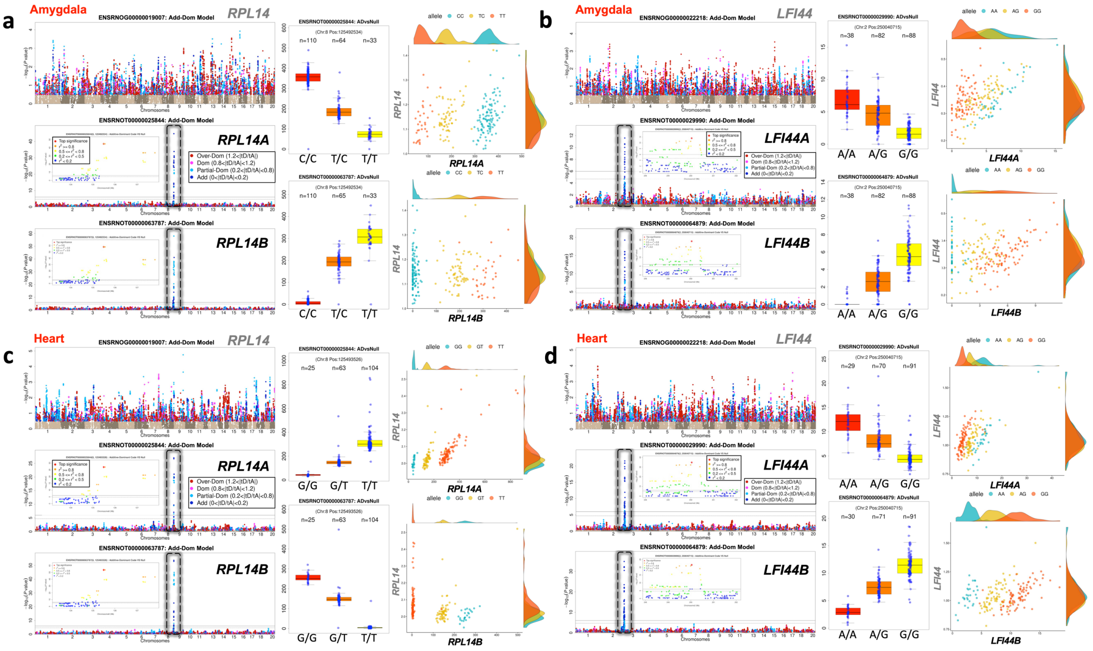

Fig. S7.1 Transcript-specific antagonistic dominant eQTLs of *RPL14* and *LFI44* in HS rat amygdala and heart. (a, c) Plots for *RPL14* (a, c) and *LFI44* (b, d) of HS rat amygdala (two upper panels) and heart (two lower panels). On the left part of each panel, the top Manhattan plot is based on their overall gene expression level, showing no genome-wide significant eQTLs, the middle and lower Manhattan plots are based on their two transcripts' expression levels, as well as their corresponding regional plots and phenotypic distribution boxplots of the peak SNPs. Within each Manhattan plot, all the SNPs with -$\log_{10}(P)$ values > 0.5 are coloured by dominance classification. On the right each panel are shown scatter plots of the correlations of expression levels between each transcript with the corresponding overall gene expression. Within each scatter plot, one dot represents one sample, the dot colors indicate the genotype of the peak SNP.

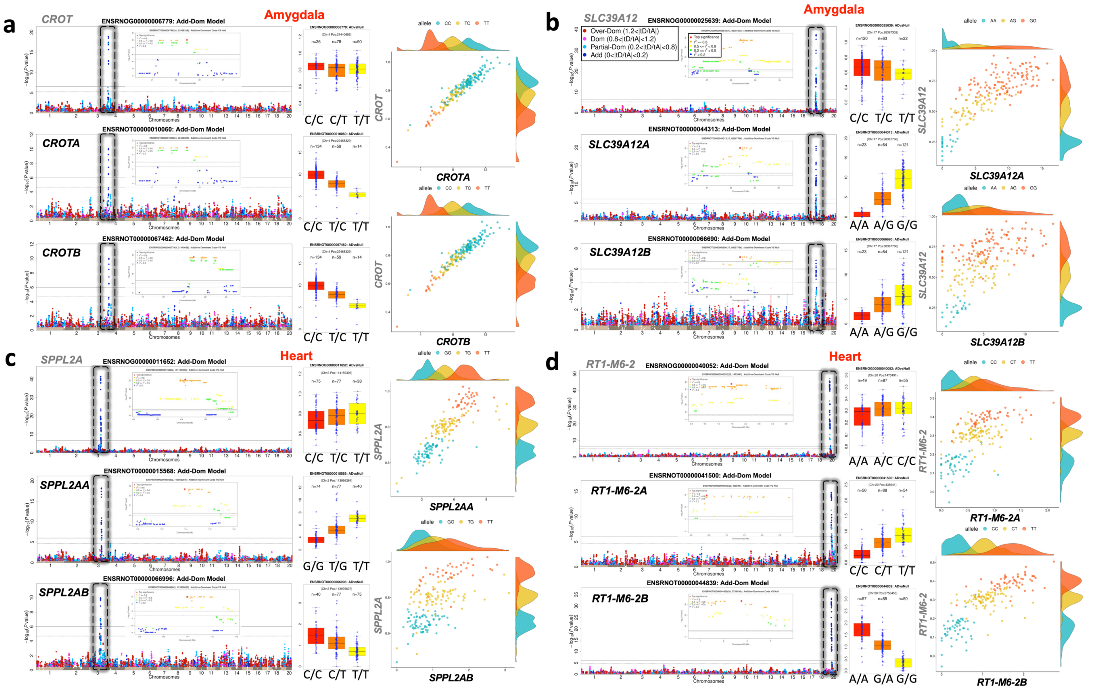

Fig. S7.2 Transcript-specific synergistic eQTLs in HS rats. (a-b) Plots of *CROT* (a) and *SLC39A12* (b) of HS rat amygdala. (c-d) Plots of *SPPL2A* (c) and *RT1-M6-2* (d) of HS rat heart. On the left part of each panel, the top Manhattan plot is based on their overall gene expression level, showing no genome-wide significant eQTLs, the middle and lower Manhattan plots are based on their two transcripts' expression levels, as well as their corresponding regional plots and phenotypic distribution boxplots of the peak SNPs. Within each Manhattan plot, all the SNPs with $-\log_{10}(P)$ values > 0.5 are coloured by dominance classification. On the right each panel are shown scatter plots of the correlations of expression levels between each transcript with the corresponding overall gene expression. Within each scatter plot, one dot represents one sample, the dot colors indicate the genotype of the peak SNP.

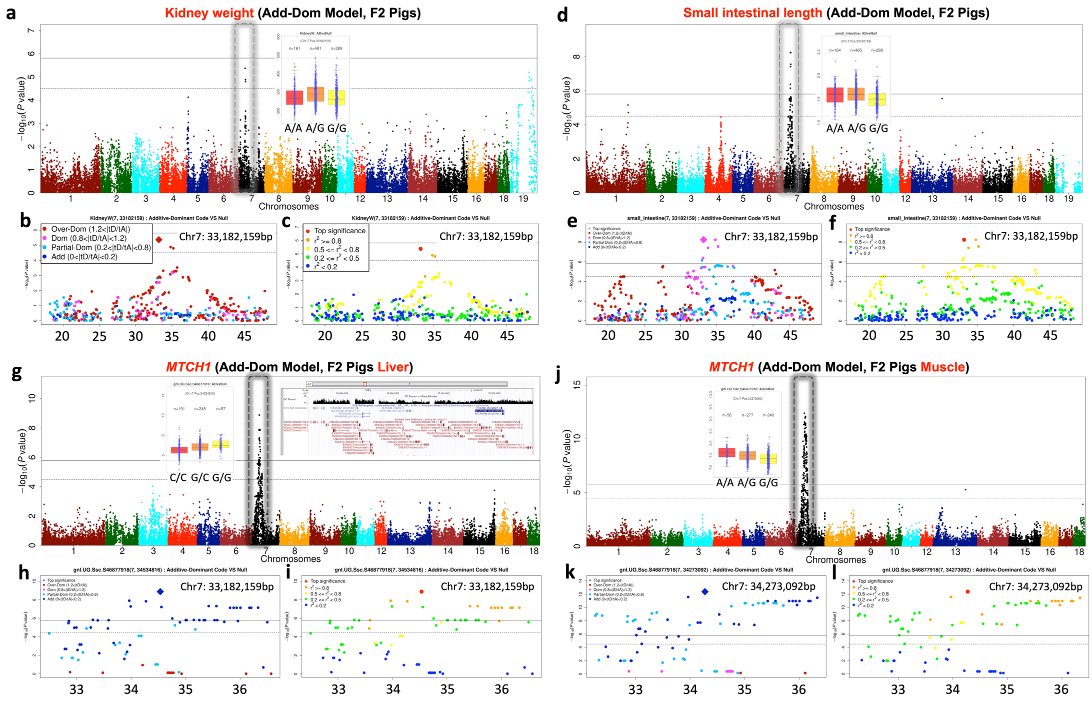

Fig. S8.1 A pleiotropic over-dominant pig QTL for kidney weight and small intestinal length colocalized with *MTCH1* eQTL in F2 pigs. Manhattan plots and regional Manhattan plots (chr7: 37Mb-38Mb) (left, blue/red colors represents the QTL dominance types; right, blue/red colors represents the LD (r^2) with the peak SNP) are shown based on AD model GWAS for (1) kidney weight GWAS using array SNPs (a-c), (2) small intestinal length using array SNPs (d-f), as well as the *MTCH1* expression level in F2 pigs liver (g-i) and muscle (j-l), respectively. The gene structure plot (g, right upper) from UCSC Genome Browser illustrating *MTCH1* as the nearest gene to the fine-mapped QTL regions of two physical traits (a, d) based on array SNPs.

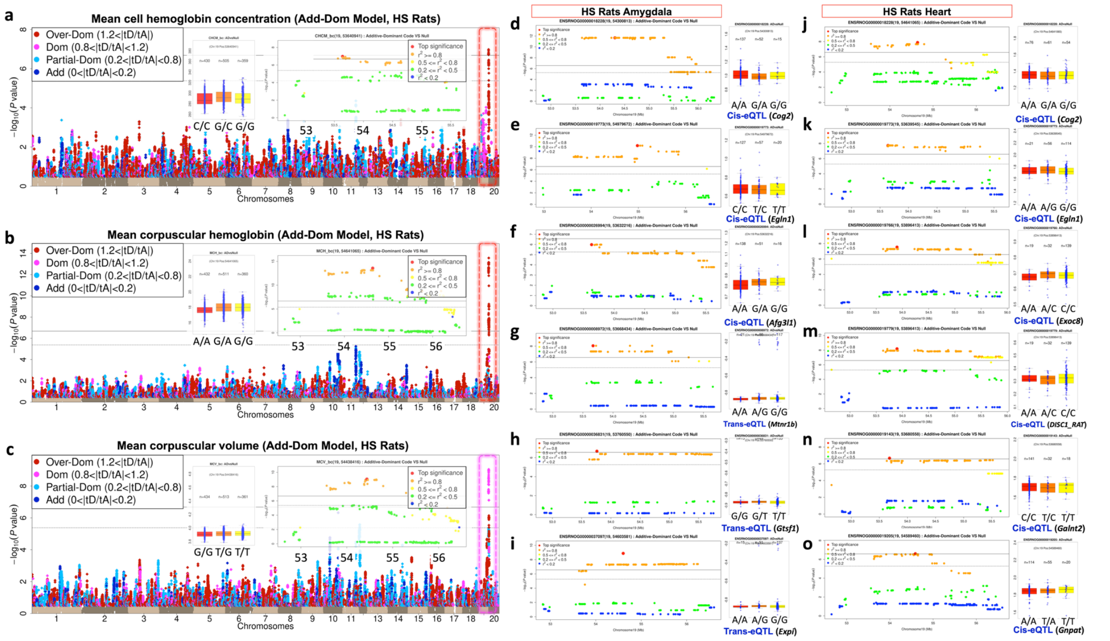

Fig. S8.2 Dominant QTLs for three rat hematology traits colocalized with cis/trans eQTLs in rat amygdala and heart (chr19: 53 Mb-55 Mb). (a-c) Manhattan plots, regional Manhattan plots and phenotypic distribution boxplots of the corresponding peak SNPs of three hematology traits in HS rats, (a) mean cell hemoglobin concentration (b) mean corpuscular hemoglobin (c) mean corpuscular volume. Dashed boxes are used to highlight the corresponding QTL regions. (d-i) Regional Manhattan plots and boxplots of colocalized eQTLs of six genes in rat amygdala, including (d-f) three cis-eQTLs of *Cog2* (CD-eQTL), *Egln1* (A-eQTL) and *Afg3l1* (PD-eQTL), and three trans-eQTLs of *Mtnr1b* (CD-eQTL), *Gtsf1* (CD-eQTL) and *Expi* (CD-eQTL). (j-o) Regional Manhattan plots and boxplots of colocalized six cis-eQTLs in rat heart, including *Cog2* (PD-eQTL), *Egln1* (PD-eQTL), *Exoc8* (PD-eQTL), *Disc1_Rat* (PD-eQTL), *Galnt2* (A-eQTL) and *Gnpat* (PD-eQTL). All GWAS based on AD model.

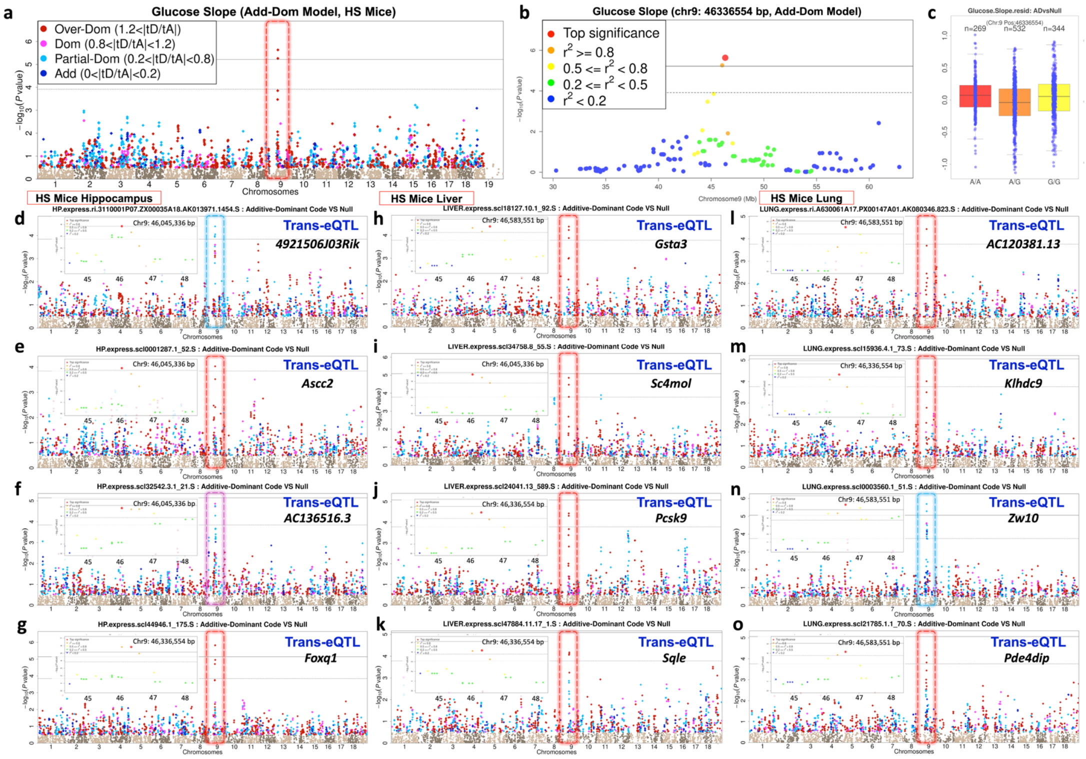

Fig. S8.3 An over-dominant QTL for mouse glucose slope colocalized with eQTLs in mouse amygdala, liver and lung. (a-c) Manhattan plots, regional Manhattan plots at (chr9: 45 Mb-47 Mb) and phenotypic distribution boxplots of the corresponding peak SNPs of mean glucose slope in HS mice. (d-g) Manhattan plots and regional plots of four colocalized trans-eQTLs in mouse hippocampus, including *4921506J03Rik* (OD-eQTL), *Ascc2* (OD-eQTL), *AC136516.3* (CD-eQTL) and *Foxq1* (OD-eQTL). (h-k) Manhattan plots and regional plots of four colocalized trans-eQTLs in mouse liver, including *Gsta3* (OD-eQTL), *Sc4mol* (OD-eQTL), *Pcsk9* (OD-eQTL) and *Sqle* (OD-eQTL). (l-o) Manhattan plots and regional plots of four colocalized trans-eQTLs in mouse lung, including *AC120381.13* (OD-eQTL), *Klhdc9* (OD-eQTL), *Zw10* (CD-eQTL) and *Pde4dip* (OD-eQTL). All QTLs based on AD model.
