# Supplementary material for: Dominance is common in mammals and is associated with trans-acting gene expression and alternative splicing
Source: Genome Biol. 2023 Sep 29;24:215. doi: 10.1186/s13059-023-03060-2 (PMC10540365; doi:10.1186/s13059-023-03060-2)
Supplement: Supplementary file 15 — Additional file 15. Review history. [file 13059_2023_3060_MOESM15_ESM.docx]

Review History

**First round of review**

**Reviewer 1**

**Were you able to assess all statistics in the manuscript, including the appropriateness of statistical tests used?**

Yes, and I have assessed the statistics in my report.

**Comments to author:**

The authors present a quantitative analysis of the prevalence of dominance in genetic effects on traits in pigs, rats, and mice. These traits include gene expression, allowing for dominance effects to be measured genome- and transcriptome-wide. Overall I found this study to be insightful and the results should be useful, particularly for animal genetics. My concerns and other comments are below.

1. Much of the text in the figures is extremely small. A lot of it would need to be scaled up by maybe a factor of ten just to be readable. This may require major reworking of the figures.

2. Classifications of QTLs into additive, partial-dominant, complete-dominant, and over-dominant are based on ranges of log-ratios of t_Dom / t_Add. This is described in the Methods on page 21, but I think it would be helpful to briefly explain why these thresholds correspond to those four categories. In particular, why the case of t_Dom = t_Add signifies complete dominance. On first glance, one might think that those t-stats being equal means that the dominant and additive effects are equal, signifying partial dominance. But perhaps they are weighted equally in case of CD because the mean of the two genotype encodings, 0/1/2 and 0/1/0, is 0/1/1, corresponding to CD. Whatever the explanation, it is worth stating explicitly since these dominance categories are compared extensively.

3. What is the interpration of observing multiple types of associations (additive, dominant) for different SNPs at a single QTL locus, as often seen in the Manhattan plot figures? Is the assumption that there is one mechanism of effect, even a single causal SNP, but noise and uncertainty lead to conflicting labels? Or can there be something real about seeing SNPs with different degrees of dominance at the same locus for a single trait? This would be good to discuss.

4. In the Abstract and in the Results on page 15, the results are said to suggest a mechanism for dominance in gene expression, based on the pattern of dominance effects on isoform-level expression. I don't think these results can be sufficiently understood at the level of mechanisms for dominance, especially since the sequence-level or exon-level differences among isoforms were not considered, only that they were different forms of a gene.

4a. This seems related to the statement on page 17 that "selecting on dominance allows us to exclude, for example, additive eQTLs underlying dominance physiological QTLs, which are less likely to be causal." If that has been demonstrated in previous studies it should be cited, but I don't see any particular reason why an eQTL that causes a dominance physiological QTL would be expected to also be dominance. Between mRNA expression and the manifestation of the physiological trait, there are many processes affected in a causal chain, some of which may not preserve linearity, and additive variation could lead to non-additive variation at some stage in that process.

5. I recommend changing terminology from "transcript" to "isoform", e.g. isoform-level expression. Transcript really just refers to the RNA copies of a gene. While "transcript" is often used as shorthand for "unique transcript", i.e. isoform, it leads to confusion. For example, transcripts per million (TPM) is a standard unit used to quantify expression, regardless of whether it is quantified at the gene, isoform, or any other level. So phrases like "multiple transcripts" or "more transcripts" sound like they are referring to the level of expression rather than diversity of isoforms.

6. "Alternative" vs. "alternate" transcripts seem to be used interchangeably. I believe only "alternative" should be used.

7. Figure 6a-h provide a very nice visual comparison of additive/dominant cis/trans-eQTLs. For 6i-j though, Venn diagrams do not seem to be the right visualization for this data. Venn diagrams should represent items that could fall in any combination of the categories, so at first I was puzzled about what the empty regions signified. Perhaps two-by-two contingency tables would be more easily understood.

8. Page 17 line 11: "we cannot not prove causality" -> "we cannot prove causality"

**Reviewer 2**

**Were you able to assess all statistics in the manuscript, including the appropriateness of statistical tests used?**

Yes, and I have assessed the statistics in my report.

**Comments to author:**

In this paper, the authors examine dominance in three mammalian genetic mapping populations (an F2 cross, and rat and mouse heterogeneous stock populations). Using a series of state-of-the-art mixed model analyses for genetic mapping, they quantify variance components for organism-level and gene expression traits and proceed to map (e)QTLs using models ranging from additive inheritance to various degrees of dominance.

The authors find that, on average, dominance accounts for an average of 25% of heritable variance, with relatively high dominance contributions for blood and immune traits. There were dozens of QTLs only detected using dominance models. Cis eQTLs tended to be mostly additive, with more dominance at trans-acting loci.

Overall, this is a competently done and interesting paper that serves as an important reminder to consider models beyond simple additivity, at least when working with experimental populations (as the authors point out, outbred human GWAS data remains less than ideal for the detection of dominance due to often low minor allele frequencies).

The extension to gene expression is particularly nice, even though these sections became fairly descriptive in some parts, without really doing much with the results beyond simply enumerating them. For example, there is a missed opportunity to do more with the combination of eQTLs and physiological QTLs to see if, say, dominance eQTLs tend to occur at dominance physiological QTLs more often than expected, perhaps because these eQTLs are the source of the physiological QTLs. It is somewhat disappointing to relegate this area to a passing mention to supplementary materials in the Discussion (page 17 line 8).

Major comments:

1. The eQTL section is extremely descriptive and simply keeps on enumerating examples without much interpretation or biological context. For example, the text starting page 12 line 26, which might just as well have been a table. What are we supposed to take away from these results?

2. Similar comment for Figure 5 - what, if anything, should we see here, beyond examples of Manhattan plots? Is there something interesting about these genes or this locus? As it currently stands, this Figure and the text describing it seem like extraneous detail without providing much insight.

3. I couldn't follow the numbers describing the hotspots. E.g. in pigs, how can there be several hundreds of hotspots that then only affect <2000 genes (in total? per hotspot?)? Does each of these genes have dozens of trans eQTLs? How does this compare to the number of detected eQTLs shown in Figure 4 where there are maybe 11000 total eQTLs summed across all classes? These numbers don't seem to add up. Please clarify.

4. The font sizes in the figures are much too small. Figures 1h and 2a,b,c are particularly egregious examples, but the font size for essentially all axis labels in all figures should be increased substantially.

Minor comments:

5. Page 3 line 16: I'm not sure what is meant by understanding dominance "in the round". Is this a typo? If not, maybe rephrase this?

6. Figure 3a: make the two zoomed-in insets have the same y-scale

7. Figure 3: I am somewhat confused why QTLs found with the additive model still are assigned to the various dominance classes. E.g. in Figure 7a: the title says "Additive Model", yet the highlighted hit is mostly "dom" or "partial dom". Is this because the model indicated in the panel title was only used in the scan, but the assignment into dominance classes was then done the same way irrespective of which model was used in the scan? It would be good to explain this more.

8. Page 11 line 10: "Although the standard errors of the estimated variance components are large…". Please add these standard errors to the barplots in Figure 4A.

9. Figure 5e,f,g,h: the gene name Nf32l1 has an "E" where the "3" should be, according to the text (maybe it is the text that gives the wrong name though).

10. Figure 5 and/or the text describing it: what is the correlation (scatterplot) between the expression levels of the two cis genes TBX21 and NF32l1?

**Authors Response**

**Point-by-point responses to the reviewers’ comments:**

Reviewer #1: The authors present a quantitative analysis of the prevalence of dominance in genetic effects on traits in pigs, rats, and mice. These traits include gene expression, allowing for dominance effects to be measured genome- and transcriptome-wide. Overall I found this study to be insightful and the results should be useful, particularly for animal genetics. My concerns and other comments are below.

1. Much of the text in the figures is extremely small. A lot of it would need to be scaled up by maybe a factor of ten just to be readable. This may require major reworking of the figures.

*Response: We have remade all the figures and increased the font sizes. We have also restructured Figures 6,7 to make them larger and therefore easier to read and added two new figures (Fig S1.1 and Fig S3.4) to support the manuscript.*

*Specifically, the figures have been changed as follows:*

*Fig 1: (1) Increased the sizes and axis labels of all upper-right inset bar plots (a-f), remove their tiny y-lab texts and semi-transparent them for a better visualization of their background scatter plot; (2) Increased the font sizes of trait categories tabulated in the left insets (d-f); (3) Reorganized the whole plot structure and increase the sizes and axis labels of all bar plots (g-i).*

*Fig 2: (1) Increased the font sizes of trait categories, font sizes of the dots represent the significance level; (2) Increased the y-axis labels of three scatter plots (a-c); (3) Increased the axis labels and font sizes of three bar plots (d-f); (4) Increased the font sizes of three Venn plots (h-j).*

*Fig 3: (1) Remade the two zoomed-in insets to have the same y-scale (a); (1) Increased the sizes, the titles and the x-axis and y-axis labels of each Manhattan plot and their corresponding regional QTL plots and phenotype-genotype distribution plots at peak SNPs (a-f); (2) Removed the titles of all insets and added the peak SNP information in the titles of the main plots; (3) Added the species classification titles on the left side.*

*Fig 4: Increased the font sizes, number sizes and axis labels of bar plots (a-e).*

*Fig 5: (1) Replaced the Venn plot with a detailed two-by-two contingency table; (2) Increased all the font sizes and axis labels of eight scatter plots (a-h); (3) Increased the font sizes of trans-acting enrichment summary table (i); (4) Increased the axis labels and legends of line plot (j).*

*Fig 6: (1) Restructured the figure to make the two main parts are above each other so that it takes up a full A4 page, i.e. a-d above e-h; (2) Increased the sizes, the titles and the x-axis and y-axis labels of all Manhattan plots and the corresponding regional QTL plots at peak SNPs (a-h); (3) Increased the legend sizes of all inserted scatter plots (a-h).*

*Fig 7: (1) Restructured the figure so that it takes up a full A4 page, i.e. a-f above g-l; (2) Increased the sizes, the titles as well as the x-axis and y-axis labels of all 6 Manhattan plots and the corresponding 4 regional QTL plots and 4 phenotype-genotype distribution plots at peak SNPs (a-c, g-i); (3) Increased the legend sizes of all inserted scatter plots (d-f, j-l).*

*Fig S1.1: Added the sample graph of different dominance classifications and different coordinate systems for QTL category comparison (Figure R1 in response).*

*Fig S1.2: (1) Increased the size of the legend and y-axis labels in three bar plots (a-c); (2) We add the note (?2>h2) for bar plots framed in red dotted boxes.*

*Fig S2.1: Increased the sizes of titles, x-axis and y-axis labels of scatter plots (a-f), and created a bigger legend on the top.*

*Fig S2.2: (1) Increased text sizes and numbers of Venn diagrams (a-c); (2) Increased the x-axis and y-axis labels of all violin plots, and added the labels of each model comparison (d-l).*

*Fig S3.1 (1) Increased the sizes of trait names and potential casual gene names of 4 GWAS results (a, e, i, m); (2) Increased the legend sizes of each regional Manhattan plots (c, g, k, o); (3) Increased the axis labels and sizes of annotation texts of each peak SNP position in 4 boxplots (d, h, l, p).*

*Fig S3.2 (1) Increased the legend sizes of 6 regional Manhattan plots (b-c, f-g, j-k); (2) Increased the sizes of annotation text of peak SNP position and genotype in 3 boxplots (d, h, l).*

*Fig S3.3 (1) Increased the titles of all Manhattan and regional Manhattan plots (a-h); (2) Increased the legend sizes as well as the x-axis and y-axis labels of 4 regional Manhattan plots (b, d, f, h).*

*Fig S3.4: Added the new response Fig R2 (see below).*

*Fig S4.1 & Fig S4.2 (1) Increased the sizes of the titles of all Manhattan plots and boxplots (a-l); (2) Added a legend of Manhattan plot (a) and increased the legend of regional Manhattan plot (c); (3) Increased the axis labels of 3 regional Manhattan plots; (4) Increased the sizes of genotype texts of 3 boxplots.*

*Fig S4.3 (1) Increased the legend sizes of Manhattan plot and regional Manhattan plot (a); (2) Increased the traits names on the right of the plot; (3) Restructured this figure to make them lager and therefore easier to read.*

*Fig S5.1 to Fig S5.3 (1) Replaced the Venn plot with a detailed two-by-two contingency table; (2) Increased all the font sizes and axis labels of 8 scatter plots (a-h); (3) Increased the font sizes of trans-acting enrichment summary table (i); (4) Increased the axis labels and legends of line plot (j).*

*Fig S6.1 (1) Increased the legend sizes of Manhattan plots (a, e); (2) Increased the gene names and texts of 6 scatter plots; (3) Restructured this figure to make them lager and therefore easier to read.*

*Fig S6.2 (1) Increased the legend sizes of 4 Manhattan plots and 8 regional Manhattan plots (a-l); (2) Increased the axis labels of 8 regional Manhattan plots (a, c, d, f, g, i, j, l); (3) Restructured this figure to increase the sizes of boxplots (m, n) and increased the annotation sizes of their genotypes.*

*Fig S6.3 (1) Restructured the figure to make the first two columns are separated from the last two columns and further extracted the inserted boxplots to be one independent column so that it takes up two full A4 page to make them larger; (2) Added the legend of Manhattan plots (b1, d1).*

*Fig S6.4 to Fig S6.6 (1) Restructured the figure to extract the inserted regional Manhattan plots and boxplots to be separated columns to make them larger; (2) Added the legend of the Manhattan plots and regional Manhattan plots.*

*Fig S6.7 Added the legend of the Manhattan plots and regional Manhattan plots.*

*Fig S7.1 (1) Increased the sizes of the legend of 8 scatter plots; (2) Increase the sizes of texts and axis labels of 8 boxplots; (3) Increased the sizes of the legends of the Manhattan plots and regional Manhattan plots.*

*Fig S7.2 (1) Increased the sizes of the legend of 8 scatter plots; (2) Increase the sizes of texts and axis labels of 12 boxplots; (3) Increased the sizes of the legends of the Manhattan plots and regional Manhattan plots.*

*Fig S8.1 (1) Increased the axis labels of 8 regional Manhattan plots; (2) Increased the sizes of the legends of the Manhattan plots and regional Manhattan plots.*

*Fig S8.2 (1) Increased the legends and axis labels of inserted regional Manhattan plots (a-c); (2) Added the legend of the Manhattan plots (a-c); (3) Increase the sizes of texts of 15 boxplots.*

*Fig S8.3 (1) Increased the legend sizes of plots (a, b); (2) Increased the axis labels of inserted regional Manhattan plots.*

2. Classifications of QTLs into additive, partial-dominant, complete-dominant, and over-dominant are based on ranges of log-ratios of t_Dom / t_Add. This is described in the Methods on page 21, but I think it would be helpful to briefly explain why these thresholds correspond to those four categories. In particular, why the case of t_Dom = t_Add signifies complete dominance. On first glance, one might think that those t-stats being equal means that the dominant and additive effects are equal, signifying partial dominance. But perhaps they are weighted equally in case of CD because the mean of the two genotype encodings, 0/1/2 and 0/1/0, is 0/1/1, corresponding to CD. Whatever the explanation, it is worth stating explicitly since these dominance categories are compared extensively.

*Response: The QTL classification method we used adapted a long-standing measurement (the degree of dominance) from previous studies (Stuber, et al., 1987 Crop Science; Lanzhi, et al., 2008 Genetics), which was based on the ratio of additive (beta_Add) and dominance (beta_Dom) effect to classify QTL, and the thresholds we used (additive: 0-0.2; partial-dominance: 0.2-0.8; complete-dominance: 0.8-1.2; over-dominance: >1.2) are derived from those studies. In the MS we modified the definition to use the ratio of the t-statistics |t_Dom/t_Add | to account for uncertainty in the parameter estimates. We agree that any qualitative classification based a continuous statistic involves some arbitrariness in setting thresholds. We have noted this in the text. The case t_Add=t_Dom implies the additive effect of allele A equals the interaction effect between allele A and B (dominance effect), implying that the heterozygotes with genotype AB display the same phenotype as the homozygotes with genotype BB (2a = a + d), i.e. complete dominance (see Supplementary Figure S1.1). The log transformation log_2(|t_Dom/t_Add | (Figure 2a-c in the revised manuscript) does not alter the classification but presents a clearer visualization because it spreads out the QTLs compared to the original radial visualization (Figure S2.1 a-f in the revised supplementary). We have added this explanation for the QTL classification thresholds together with the references mentioned above in the revised manuscript (Page 8, Comment #1).*

3. What is the interpretation of observing multiple types of associations (additive, dominant) for different SNPs at a single QTL locus, as often seen in the Manhattan plot figures? Is the assumption that there is one mechanism of effect, even a single causal SNP, but noise and uncertainty lead to conflicting labels? Or can there be something real about seeing SNPs with different degrees of dominance at the same locus for a single trait? This would be good to discuss.

*Response: As we mentioned above, the dominance classification of each SNP within a QTL locus was determined by the value of the t-ratio |t_Dom/t_Add |, based on the additive (beta_Add) and dominance (beta_Dom) effects of the SNP in question. Where different SNPs under a QTL have different linkage disequilibrium patterns with the peak SNP, their beta values (beta_Add and beta_Dom) their dominance classification might also be different, especially if there are multiple independent causal variants under a QTL. In other words, the SNP dominance classifications are closely related to their LD patterns; SNPs in strong LD tend to have same or similar classifications. Whilst we agree with the reviewer on the potential for noise and uncertainty to affect dominance classification, we have not observed it in our data.*

*For the Manhattan plots in Figures 3, 6 and 7, we colour the SNPs that indicate their degree of dominance Blue - additive; Sky blue - partial-dominant; Purple - complete-dominant; Red - over-dominant). We also include inset plots showing the R2 LD values between each SNP and the peak SNP, but now coloured by the extent of LD. Comparison of the Manhattan and LD plots confirms that SNPs in LD have similar dominance classifications.*

*We show here a representative example of an HS mice immunological trait (the proportion of CD4+ cells in CD3+ cells, Figure R2 in this response letter), in which two independent QTLs with different association types (one over-dominant QTL in Figure R2d and one partial-dominant QTL in Figure R2e in this response letter) were detected by the conditional GWAS (by regressing the peak SNPs iteratively). It is also worth noting that the former QTL (chr17: 35,276,617 bp) closer to an immune function related gene Bat3 (HLA-B associated transcript 3) and the latter QTL (chr17: 33,744,539 bp) closer to a cell adhesion and motility function related gene Myo1f (Myosin IF). We have added the above explanation in the discussion section of the revised manuscript and a new figure of a conditional GWAS establising there are independent genetic effects at the locushas been included in the supplement as Fig S3.4 (Page 9 lines 28-30 & Page 10 lines 1-7, Comments #2, #3, #3a).*

4. In the Abstract and in the Results on page 15, the results are said to suggest a mechanism for dominance in gene expression, based on the pattern of dominance effects on isoform-level expression. I don't think these results can be sufficiently understood at the level of mechanisms for dominance, especially since the sequence-level or exon-level differences among isoforms were not considered, only that they were different forms of a gene.

4a. This seems related to the statement on page 17 that "selecting on dominance allows us to exclude, for example, additive eQTLs underlying dominance physiological QTLs, which are less likely to be causal." If that has been demonstrated in previous studies it should be cited, but I don't see any particular reason why an eQTL that causes a dominance physiological QTL would be expected to also be dominance. Between mRNA expression and the manifestation of the physiological trait, there are many processes affected in a causal chain, some of which may not preserve linearity, and additive variation could lead to non-additive variation at some stage in that process.

*Response: Thank you for the comment. If a physiological phenotype depends on an underlying gene expression trait then it is simplest to assume a linear relationship between the two quantities. Consequently if the gene expression depends on a genotype additively then we would expect the phenotype to also do so. Equally, if the expression level depends on a genotype in a dominant fashion then so would the phenotype. Whilst there may well be situations where phenotype depends on expression in a non-linear fashion, and which could potentially convert an additive genetic effect on expression to a non-linear effect on phenotype, it is harder to imagine a non-linear effect on expression being converted into a linear effect on phenotype. For example, in the case of complete dominance when the heterozygote effect on expression is equal to that of one of the homozygotes, at the phenotype level there is no genotype information available to deduce which individuals are heterozygous. This loss of information makes it difficult to undo the effects of dominance. We have added some text to the discussion making this point (P17, Comment #4).*

5. I recommend changing terminology from "transcript" to "isoform", e.g. isoform-level expression. Transcript really just refers to the RNA copies of a gene. While "transcript" is often used as shorthand for "unique transcript", i.e. isoform, it leads to confusion. For example, transcripts per million (TPM) is a standard unit used to quantify expression, regardless of whether it is quantified at the gene, isoform, or any other level. So phrases like "multiple transcripts" or "more transcripts" sound like they are referring to the level of expression rather than diversity of isoforms.

*Response: We have changed the "transcript" to "isoform" throughout.*

6. "Alternative" vs. "alternate" transcripts seem to be used interchangeably. I believe only "alternative" should be used.

*Response: We have replaced ÒalternateÓ with ÒalternativeÓ.*

7. Figure 6a-h provide a very nice visual comparison of additive/dominant cis/trans-eQTLs. For 6i-j though, Venn diagrams do not seem to be the right visualization for this data. Venn diagrams should represent items that could fall in any combination of the categories, so at first I was puzzled about what the empty regions signified. Perhaps two-by-two contingency tables would be more easily understood.

*Response: We have replaced the Venn diagrams with a two-by-two contingency table in Figure 6 and Figure S6-1 to S6-3.*

8. Page 17 line 11: "we cannot not prove causality" -> "we cannot prove causality"

*Response: Thanks for spotting the typo, we have corrected it.*

Reviewer #2: In this paper, the authors examine dominance in three mammalian genetic mapping populations (an F2 cross, and rat and mouse heterogeneous stock populations). Using a series of state-of-the-art mixed model analyses for genetic mapping, they quantify variance components for organism-level and gene expression traits and proceed to map (e)QTLs using models ranging from additive inheritance to various degrees of dominance.

The authors find that, on average, dominance accounts for an average of 25% of heritable variance, with relatively high dominance contributions for blood and immune traits. There were dozens of QTLs only detected using dominance models. Cis eQTLs tended to be mostly additive, with more dominance at trans-acting loci.

Overall, this is a competently done and interesting paper that serves as an important reminder to consider models beyond simple additivity, at least when working with experimental populations (as the authors point out, outbred human GWAS data remains less than ideal for the detection of dominance due to often low minor allele frequencies).

The extension to gene expression is particularly nice, even though these sections became fairly descriptive in some parts, without really doing much with the results beyond simply enumerating them. For example, there is a missed opportunity to do more with the combination of eQTLs and physiological QTLs to see if, say, dominance eQTLs tend to occur at dominance physiological QTLs more often than expected, perhaps because these eQTLs are the source of the physiological QTLs. It is somewhat disappointing to relegate this area to a passing mention to supplementary materials in the Discussion (page 17 line 8).

Major comments:

1. The eQTL section is extremely descriptive and simply keeps on enumerating examples without much interpretation or biological context. For example, the text starting page 12 line 26, which might just as well have been a table. What are we supposed to take away from these results?

*Response: We have substantially rewritten and shortened the eQTL section and made a table (Supplementary Table S12-7) to summarise many of the results mentioned on page 12 line 26. The main purpose that we listed the tissue-consistent eQTLs (tc-eQTLs) and tissue-specific eQTLs (ts-eQTLs) was to comprehensively summarise the eQTL results and also to analysis the consistency and specificity of dominance eQTLs across different tissues within one species.*

2. Similar comment for Figure 5 - what, if anything, should we see here, beyond examples of Manhattan plots? Is there something interesting about these genes or this locus? As it currently stands, this Figure and the text describing it seem like extraneous detail without providing much insight.

*Response: Figure 5 (we have renamed it as Figure 6 in the revised manuscript) shows a representative dominance gene-level hotspot at chr10: 85Mb-86Mb in HS rat heart. This hotspot is complex: it has two cis-eQTLs for the transcription factors (a) Tbx21 (over-dominant) and (e) Nf32l1 (additive), that link to six trans-eQTLs. The point we wished to emphasize, in addition to the general conclusions about the importance of dominance, is that many loci exhibit very complex behaviors. We have added explanatory text on page 13/14 (Comment #5).*

3. I couldn't follow the numbers describing the hotspots. E.g. in pigs, how can there be several hundreds of hotspots that then only affect <2000 genes (in total? per hotspot?)? Does each of these genes have dozens of trans eQTLs? How does this compare to the number of detected eQTLs shown in Figure 4 where there are maybe 11000 total eQTLs summed across all classes? These numbers don't seem to add up. Please clarify.

*Response: We apologise, our description of hotspots in the manuscript was misleading. We initially identified hotspots using suggestive significance thresholds. For example, among suggestive significant pig eQTLs, there are 11,596 and 12,157 overlapping eQTLs that localize to just 594 and 606 separate regions in liver and muscle respectively (overlaps between eQTLs employed the 2-LOD drop method to define eQTL confidence intervals). In order report the data we then raised the stringency to (-log10(P)>10) which left 2,333 liver and 2,088 muscle exemplar eQTLs from 537 and 479 separate regions (Supplementary Table S11-1 and S11-2). Thus these high-stringency eQTLs tag most of the hotspots. We have modified the text on page 13 in the revision accordingly (comment 6).*

4. The font sizes in the figures are much too small. Figures 1h and 2a,b,c are particularly egregious examples, but the font size for essentially all axis labels in all figures should be increased substantially.

*Response: Thank you for the advice, we have increased the font sizes of all the figures, especially for Figure 1 and Figure 2. We have also restructured Figures 6 and 7 to make them easier to read. Please see detailed response above.*

Minor comments:

5. Page 3 line 16: I'm not sure what is meant by understanding dominance "in the round". Is this a typo? If not, maybe rephrase this?

*Response: We have deleted the phrase.*

6. Figure 3a: make the two zoomed-in insets have the same y-scale

*Response: We have remade those two zoomed-in insets with the same y-scale.*

7. Figure 3: I am somewhat confused why QTLs found with the additive model still are assigned to the various dominance classes. E.g. in Figure 7a: the title says "Additive Model", yet the highlighted hit is mostly "dom" or "partial dom". Is this because the model indicated in the panel title was only used in the scan, but the assignment into dominance classes was then done the same way irrespective of which model was used in the scan? It would be good to explain this more.

*Response: The colors of SNPs in Manhattan plots (e.g. Figure 3a in the revised manuscript) represent dominance classifications as determined by the values of |t_Dom/t_Add | of each locus, and which were always calculated by the Add-Dom model irrespective of which model was used to calculate the P-values.*

8. Page 11 line 10: "Although the standard errors of the estimated variance components are large". Please add these standard errors to the barplots in Figure 4A.

*Response: The "standard error" we mentioned on page 11 line 10 refers to the standard error of variance components of each single gene reported by the REML algorithm using GCTA (Supplementary Table S7 in the revised manuscript). This is calculated from the inverse of the matrix of second derivatives (the Hessian) in the maximum likelihood estimation of the additive and dominance variance components, and is quite distinct from the empirical standard errors of the means of these components across all the of genes. That is, we calculated the average variance components V_a and V_d and the average standard errors (SE_a and SE_d per species and tissue under different gene filter conditions. We also calculated the population-level standard errors of both additive and dominance variance components (SE((V_a ) ) and SE((V_d ) )) within each tissue based on different gene sets (Table R1 in this response letter). These population-level standard errors are generally tiny (little more than the width of the lines in the barplots) We do not think they are needed in the Figure 4A.*

*Table R1 | Comparison of the average additive and dominance variance components of each tissue across three populations, along with their corresponding average standard errors (**) and population-level standard errors*

9. Figure 5e,f,g,h: the gene name Nf32l1 has an "E" where the "3" should be, according to the text (maybe it is the text that gives the wrong name though).

*Response: We have corrected the typing error in text, Nfe2l1 is the right gene name.*

10. Figure 5 and/or the text describing it: what is the correlation (scatterplot) between the expression levels of the two cis genes TBX21 and NF32l1?

*Response: The correlations between Tbx21 and Nfe2l1 expression levels are -0.11 and -0.16 at the gene level and the isoform level, respectivelyxw. We also added this result in the revised manuscript (Page 13 lines 21-23). (Comment #5)*

**Second round of review**

**Reviewer 1**

The authors have sufficiently addressed most of my concerns. Further response for two of the comments:

1. The readability of the figures has been much improved. Some of the text is still extremely small, e.g. the trait labels in Figure 2a-c, but in that case the labels serve as auxiliary information, so the reader can zoom in if desired. And much of the text in Figure 6 would still be extremely small if the figure is scaled to page height, but that may or may not be an issue for this journal.

4. On the question of whether additive eQTLs leading to dominance physiological phenotypes could be ruled out, I found the response and associated manuscript edit unconvincing. Quoting and responding point-by-point:

>"If a physiological phenotype depends on an underlying gene expression trait then it is simplest to assume a linear relationship between the two quantities."

That may be simplest, but simplest does not mean correct. This idea of assuming a linear relationship seems especially out of place in a study whose purpose is to demonstrate what is missed by assuming additive effects.

>"Consequently if the gene expression depends on a genotype additively then we would expect the phenotype to also do so. Equally, if the expression level depends on a genotype in a dominant fashion then so would the phenotype."

These follow only from the above assumption that has not yet been supported, either by referring back to specific results or by citing the literature. It would surprise me if this were true. To put it in other words, under this theory it is plausible to have a non-linear relationship between genotype and gene expression (i.e. dominance eQTL), but implausible to have a non-linear relationship between gene expression and trait, despite the many more complex steps and factors leading from mRNA to the trait.

>"Whilst there may well be situations where phenotype depends on expression in a non-linear fashion, and which could potentially convert an additive genetic effect on expression to a non-linear effect on phenotype,"

This was my point, and it seems to conflict with the statement in the Discussion that my original comment questioned: "Whilst we cannot prove causality in these examples, prioritizing genes on dominance allows us to exclude, for example, additive eQTLs underlying dominance physiological QTLs, which are less likely to be causal."

>"it is harder to imagine a non-linear effect on expression being converted into a linear effect on phenotype. For example, in the case of complete dominance when the heterozygote effect on expression is equal to that of one of the homozygotes, at the phenotype level there is no genotype information available to deduce which individuals are heterozygous. This loss of information makes it difficult to undo the effects of dominance."

While I also agree with this, I am confused why this scenario is now being explained in detail in the text, when the claim that my comment questioned was that the opposite scenario, "additive eQTLs underlying dominance physiological QTLs", could be ruled out.

**Reviewer 2**

Thanks to the authors for a (mostly) thorough job on the revision. My only remaining comment is that the font sizes in the figures are still too small, especially in Figure 1 (which I had previously commented on). This figure is not acceptable as is. Text in figures needs to be legible comfortably at normal print sizes.

Other examples that must be fixed: the inset labels above the barplots in Figure 3, the “top significance” values in figure 6 and other panels with the same format. The authors need to carefully check all figures and make sure they can be read at normal viewing sizes.

**Authors Response**

**Point-by-point responses to the reviewers’ comments:**

Reviewer #1: The authors have sufficiently addressed most of my concerns. Further response for two of the comments:

1. The readability of the figures has been much improved. Some of the text is still extremely small, e.g. the trait labels in Figure 2a-c, but in that case the labels serve as auxiliary information, so the reader can zoom in if desired. And much of the text in Figure 6 would still be extremely small if the figure is scaled to page height, but that may or may not be an issue for this journal.

*Response: We have remade all the figures and increased their font sizes. We have especially restructured Figures 1, 6 to increase their inset texts and added a new column (named "Figure2_Plot_Order") in supplementary table S4 to provide clear corresponding information for the trait labels in Figure 2. Specifically, the figures have been changed as follows:*

*Fig 1: (1) Reorganized the inset trait-classification legends of scatter plots (a-f) and moved them to the bottom of each panel to increase their sizes; (2) Increased the sizes of all axis labels and figure titles of six scatter plots (a-f), and removed the histogram distribution plots on sides of the horizontal and vertical axes to make the scatter plots bigger; (3) Increased the sizes of all trait names of three barplots (g-i).*

*Fig 2: (1) Moved the inset labels above three barplots (d-f) to the bottom of Fig 2 and increased their sizes; (2) Renamed the figure titles of all barplots (d-f) and histograms (h-j) and increased their sizes; (3) Added a new column (named "Figure2_Plot_Order") in supplementary table S4 to provide clear corresponding information of each trait labels and their statistics in three scatter plots (a-c); (4) Moved the legend of barplots (d-f) to the bottom of Fig 2 for a better visualization. Note we have retained the very small phenotype labels in this figure. We can remove them if the editor requests it but on balance we thought it was best to retain them.*

*Fig 3: (1) Renamed the figure titles of all Manhattan plots and increased their size (a-f); (2) Added a new label on the right of the figure to indicate the GWAS model (Additive model or Add-Dom model) of each Manhattan plot; (3) Moved the legends of Manhattan plot and regional Manhattan plot to the bottom of Fig 3 for a better visualization and removed the reduplicate legends of other Manhattan plots; (4) Increased the label sizes of nine inset boxplots and removed the superfluous sample numbers of each boxplots (a-f).*

*Fig 6: (1) Split the whole figure into two to increase the scale, (Fig 6 and Fig 7 in new submission) so (a-d) in old Fig 6 are in new Fig 6 and (e-h) in old Fig6 are now (a-d) in new Fig 7; (2) Moved the legends of Manhattan plot and regional Manhattan plot to the bottom of Fig 6 for a better visualization and removed the reduplicate legends of other Manhattan plots; (3) Increased the sizes of all inset texts and labels of each panel; (4) Increased the size of allele genotype label above each scatter plot.*

4. On the question of whether additive eQTLs leading to dominance physiological phenotypes could be ruled out, I found the response and associated manuscript edit unconvincing. Quoting and responding point-by-point:

>"If a physiological phenotype depends on an underlying gene expression trait then it is simplest to assume a linear relationship between the two quantities."

That may be simplest, but simplest does not mean correct. This idea of assuming a linear relationship seems especially out of place in a study whose purpose is to demonstrate what is missed by assuming additive effects.

>"Consequently if the gene expression depends on a genotype additively then we would expect the phenotype to also do so. Equally, if the expression level depends on a genotype in a dominant fashion then so would the phenotype."

These follow only from the above assumption that has not yet been supported, either by referring back to specific results or by citing the literature. It would surprise me if this were true. To put it in other words, under this theory it is plausible to have a non-linear relationship between genotype and gene expression (i.e. dominance eQTL), but implausible to have a non-linear relationship between gene expression and trait, despite the many more complex steps and factors leading from mRNA to the trait.

>"Whilst there may well be situations where phenotype depends on expression in a non-linear fashion, and which could potentially convert an additive genetic effect on expression to a non-linear effect on phenotype,"

This was my point, and it seems to conflict with the statement in the Discussion that my original comment questioned: "Whilst we cannot prove causality in these examples, prioritizing genes on dominance allows us to exclude, for example, additive eQTLs underlying dominance physiological QTLs, which are less likely to be

causal."

>"it is harder to imagine a non-linear effect on expression being converted into a linear effect on phenotype. For example, in the case of complete dominance when the heterozygote effect on expression is equal to that of one of the homozygotes, at the phenotype level there is no genotype information available to deduce which individuals are heterozygous. This loss of information makes it difficult to undo the effects of dominance."

While I also agree with this, I am confused why this scenario is now being explained in detail in the text, when the claim that my comment questioned was that the opposite scenario, "additive eQTLs underlying dominance physiological QTLs", could be ruled out.

*Response: We agree with the referee and have completely rewritten this section of the Discussion. It now reads:*

*More generally, what mechanisms convert genotypes into additive or dominant phenotypes? Assuming a phenotype only depends on a given genotype via expression of a gene - i.e. it does not "see" the underlying genotype - must additive/dominance gene expression necessarily cause additive/dominance phenotypes?*

*Considering first additive gene expression, the resulting phenotype could be either additive - if that component of the phenotype variation attributable to the expression of the gene in question is proportional to that gene's expression level - or potentially complete dominant if the relationship is non-linear, for example modelling a saturation or thresholding effect. On the other hand, if gene expression is dominant in the broad sense considered here, then there is a loss of information which makes it hard or impossible to invert the non-linear dominance relationship between genotype and expression to recover an additive dependence of phenotype on genotype. For example, if the gene expression exhibits complete dominance, then there is no longer any distinction between the heterozygote and one of the homozygotes.*

*Thus, we argue that additive gene expression eQTLs could produce either additive or dominant physiological QTLs but that dominance eQTLs should only produce dominance physiological QTLs. This seems to be an important distinction, and moreover should to all types of dominance expression, including isoform. Whilst we cannot prove causality in the examples presented in this study, prioritizing genes by dominance allows us to exclude, for example, dominance eQTLs underlying additive physiological QTLs, which are less likely to be causal.*

Reviewer #2: Thanks to the authors for a (mostly) thorough job on the revision. My only remaining comment is that the font sizes in the figures are still too small, especially in Figure 1 (which I had previously commented on). This figure is not acceptable as is. Text in figures needs to be legible comfortably at normal print sizes.

Other examples that must be fixed: the inset labels above the barplots in Figure 3, the "top significance" values in figure 6 and other panels with the same format. The authors need to carefully check all figures and make sure they can be read at normal viewing sizes.

*Response: We have carefully remade all the figures and increased the font sizes to make them easier to read, especially for the Figure 1, Figure 2 and Figure 6 (now split into new figures 6 and 7) suggested by reviewer 2, as well as a new column (named "Figure2_Plot_Order") in supplementary table S4 to provide corresponding trait labels (please find details from our response to the first question from reviewer 1).*
